# Evolution and divergence of the mammalian *SAMD9*/*SAMD9L* gene family

**DOI:** 10.1186/1471-2148-13-121

**Published:** 2013-06-12

**Authors:** Ana Lemos de Matos, Jia Liu, Grant McFadden, Pedro J Esteves

**Affiliations:** 1 CIBIO - Centro de Investigação em Biodiversidade e Recursos Genéticos/InBio Laboratório Associado, Universidade do Porto, 4485-661 Vairão, Portugal; 2 Departamento de Biologia, Faculdade de Ciências, Universidade do Porto, 4169-007 Porto, Portugal; 3 Department of Molecular Genetics and Microbiology, University of Florida College of Medicine, Gainesville, Florida 32610, USA; 4 Centro de Investigação em Tecnologias da Saúde, IPSN, CESPU, 4585-116 Gandra, Portugal

**Keywords:** SAMD9, SAMD9-like, Mammals, Evolutionary history, Positive selection

## Abstract

**Background:**

The physiological functions of the human Sterile Alpha Motif Domain-containing 9 (*SAMD9*) gene and its chromosomally adjacent paralogue, *SAMD9*-like (*SAMD9L*), currently remain unknown. However, the direct links between the deleterious mutations or deletions in these two genes and several human disorders, such as inherited inflammatory calcified tumors and acute myeloid leukemia, suggest their biological importance. SAMD9 and SAMD9L have also recently been shown to play key roles in the innate immune responses to stimuli such as viral infection. We were particularly interested in understanding the mammalian evolutionary history of these two genes. The phylogeny of *SAMD9* and *SAMD9L* genes was reconstructed using the Maximum Likelihood method. Furthermore, six different methods were applied to detect SAMD9 and SAMD9L codons under selective pressure: the site-specific model M8 implemented in the codeml program in PAML software and five methods available on the Datamonkey web server, including the Single Likelihood Ancestor Counting method, the Fixed Effect Likelihood method, the Random Effect Likelihood method, the Mixed Effects Model of Evolution method and the Fast Unbiased Bayesian AppRoximation method. Additionally, the house mouse (*Mus musculus*) genome has lost the *SAMD9* gene*,* while keeping *SAMD9L* intact, prompting us to investigate whether this loss is a unique event during evolution.

**Results:**

Our evolutionary analyses suggest that *SAMD9* and *SAMD9L* arose through an ancestral gene duplication event after the divergence of Marsupialia from Placentalia. Additionally, selection analyses demonstrated that both genes have been subjected to positive evolutionary selection. The absence of either *SAMD9* or *SAMD9L* genes from some mammalian species supports a partial functional redundancy between the two genes.

**Conclusions:**

To the best of our knowledge, this work is the first study on the evolutionary history of mammalian *SAMD9* and *SAMD9L* genes. We conclude that evolutionary selective pressure has acted on both of these two genes since their divergence, suggesting their importance in multiple cellular processes, such as the immune responses to viral pathogens.

## Background

The Sterile Alpha Motif Domain-containing 9 (*SAMD9*) gene is located in chromosome 7q21.2 of the human genome, and is adjacent to its close paralogue, *SAMD9*-like (*SAMD9L*), in a head-to-tail position [1,2] and separated by approximately 12 kb. The physiological functions of both *SAMD9* and *SAMD9L* currently remain poorly understood, but the importance of human *SAMD9* has been recently emphasized during the discovery of the genetic cause of a rare life-threatening human disease, normophosphatemic familiar tumoral calcinosis (NFTC) [3,4]. Patients with NFTC exhibited normal calcium and phosphate metabolism while developing calcified tumorous nodules at their extremities, accompanied by severe gingivitis. Two independent founder genetic events leading to the deleterious mutations in *SAMD9* are responsible for the autosomal recessive disease of NFTC [3,4]. Interestingly, these patients and their kindred are from a culturally isolated ethical group, namely Jewish-Yemenite, suggesting a potential selection pressure associated with this population [3,4]. In addition to NFTC, misregulated human *SAMD9* expression was also shown to be associated with aggressive fibromatosis, breast, and colon cancers [1].

Human *SAMD9* expression can be upregulated by tumor necrosis factor (*TNF*) [4] or by type I [5] and type II interferons (*IFN*s) [6], and it is classified as an interferon-stimulated gene (ISG). Recently, an interferon regulatory factor (*IRF-1*) binding element was identified in the promoter region of the *SAMD9* gene in humans [6], and overexpression of *IRF-1* can lead to elevated *SAMD9* gene expression [7]. All these observations suggest a key role of *SAMD9* as a signalling hub in response to innate immune stimulations. Most importantly, human *SAMD9* also has very recently been shown to possess anti-viral properties in cultured cells [8,9] emphasizing its crucial role in host defence against viral pathogens.

On the other hand, the human *SAMD9L* gene was shown to exhibit lower expression levels in breast cancer tissue than in normal breast tissue from the same patient [1]. It was also identified to be an inducible gene for type I *IFN*s (*IFNα* and *β*), and in activated human T cells the function of *SAMD9L* is correlated with its *IFN*-induced inhibitory effects on cell migration [10]. The murine *SAMD9L* gene expression was also found to be upregulated by calcitonin [11], suggesting a potential involvement in calcium homeostasis as well.

Lastly, the human *SAMD9* and *SAMD9L* genes were both classified as myeloid tumor suppressors, as they are localized within a microdeletion cluster associated with myeloid disorders, such as juvenile myelomonocytic leukemia (JMML), acute myeloid leukemia (AML), and myelodysplastic syndrome (MDS) [2]. In another study investigating altered immune responses in patients with metastatic melanoma, both *SAMD9* and *SAMD9L* expression were shown to be significantly reduced in T and B cell populations when compared with those from healthy control individuals [12]. It has been suggested that since these two proteins exhibit considerable sequence similarity, they may function redundantly or in related pathways, but it should be noted that patients with NFTC possess mutations only in *SAMD9* and thus it is likely that the two proteins perform non-identical tasks in humans.

Evolutionarily, the orthologous genes for both *SAMD9* and *SAMD9L* are highly conserved in many mammalian genomes, such as rat, primates and rabbit, but not in chicken, frog and fish species, or insects [1]. This suggests that the origin of these two related genes, possibly from an ancestral duplication event, occurred at some point after branching of the mammalian species. In addition, one intriguing fact is that the house mouse genome (*Mus musculus*, Mumu) has lost the *SAMD9* gene while maintaining *SAMD9L,* after an evolutionary chromosome breakage event [1].

The absence of *SAMD9* from the house mouse (Mumu) genome led us to question if it was a unique event restricted to this taxon and stimulated the study of *SAMD9* and *SAMD9L* evolution and divergence in different mammalian genomes. We have examined the evolutionary history and phylogeny of *SAMD9* and *SAMD9L,* using all the available and complete mammalian genomic sequences of both genes in NCBI and Ensembl databases, in order to obtain a broader understanding of the origin of these two genes. Our deduced phylogenetic tree suggests that *SAMD9* and *SAMD9L* indeed resulted from an ancestral gene duplication event that occurred after the divergence of Marsupialia from Placentalia. At the same time, we applied six different Maximum Likelihood (ML) methods to test for potential positive selective pressures exerted at the gene level, and we also looked for evidence of positive selection at the deduced protein level. The analyses revealed that SAMD9 and SAMD9L, at both the genome and deduced protein sequence levels, were under the effects of what appears to be sustained positive selective pressures. Our results suggest that these two proteins have been selected by long term environmental pressures, such as those exerted by pathogen responses that are under the control of innate immune regulators like the type I interferons.

## Results

### *SAMD9* and *SAMD9L* genes prevalence in mammals

All the available and complete mammalian *SAMD9* and *SAMD9L* genes coding sequences in the NCBI and Ensembl databases were collected, resulting in a total of fifteen *SAMD9* and nineteen *SAMD9L* genomic sequences of different species indicated in Table 1. The species collected for *SAMD9* genes fit into seven Eutheria orders, commonly designated as placental mammals, while the taxa collected for *SAMD9L* genes fit into eight placental orders. The grey short-tailed opossum, a representative of the order Didelphimorphia traditionally included in Marsupialia (pouch mammals), was the only marsupial genome to possess a complete *SAMD9L* sequence.

**Table 1:** **Mammalian *SAMD9 *and *SAMD9L *genes accession numbers from species used in phylogenetic and selection analyses**

***SAMD9***				
**Mammalian order**	**Common name**	**Species name**	**Database ID**	**Abbreviation**
Artiodactyla	Cow	*Bos taurus*	Chromosome 4: 10,302,667-10,307,412^a^	SAMD9_Bota
	Pig	*Sus scrofa*	Chromosome 9: 79,679,836-79,684,587^a^	SAMD9_Susc
Chiroptera	Little brown myotis	*Myotis lucifugus*	Scaffold AAPE02063303: 7,766-12,520^a^	SAMD9_Mylu
Lagomorpha	European rabbit	*Oryctolagus cuniculus*	Chromosome 10: 35,728,133-35,732,926^a^	SAMD9_Orcu
Perissodactyla	Horse	*Equus caballus*	Chromosome 4: 36,749,161-36,753,927 ^a^	SAMD9_Eqca
Primates	Common chimpanzee	*Pan troglodytes*	Chromosome 7: 92,731,148-92,735,917 ^a^	SAMD9_Patr
	Human	*Homo sapiens*	Chromosome 7: 92,728,829-92,747,336 ^a^	SAMD9_Hosa
	Northern white-cheeked gibbon	*Nomascus leucogenys*	SuperContig GL397261.1: 24,263,901-24,268,665 ^a^	SAMD9_Nole
	Rhesus monkey	*Macaca mulatta*	Chromosome 3: 124,130,532-124,147,894 ^a^	SAMD9_Mamu
	Sumatran orangutan	*Pongo abelii*	Chromosome 7: 83,034,053-83,038,819 ^a^	SAMD9_Poab
	Western gorilla	*Gorilla gorilla*	Chromosome 7: 90,353,240-90,358,009 ^a^	SAMD9_Gogo
Rodentia	Brown rat	*Rattus norvegicus*	XM_575365.2 ^b^	SAMD9_Rano
	Chinese hamster	*Cricetulus griseus*	AFTD01024384.1 ^b^	SAMD9_Crgr
	Domestic Guinea pig	*Cavia porcellus*	AAKN02016823.1 ^b^	SAMD9_Capo
Soricomorpha	Common shrew	*Sorex araneus*	Scaffold_257382: 52,686-57,449 ^a^	SAMD9_Soar
***SAMD9L***				
**Mammalian order**	**Common name**	**Species name**	**Database ID**	**Abbreviation**
Carnivora	Domestic dog	*Canis lupus familiaris*	XM_539422.3^b^	SAMD9L_Calu
	Giant panda	*Ailuropoda melanoleuca*	Scaffold GL192585.1: 1,477,672-1,482,429^a^	SAMD9L_Aime
Didelphimorphia(Marsupialia)	Grey short-tailed opossum	*Monodelphis domestica*	XM_001378475.1^b^	SAMD9L_Modo
Erinaceomorpha	West European hedgehog	*Erinaceus europaeus*	GeneScaffold_8766: 48,007-52,945 ^a^	SAMD9L_Ereu
Lagomorpha	European rabbit	*Oryctolagus cuniculus*	Chromosome 10: 35,699,236-35,703,990 ^a^	SAMD9L_Orcu
Perissodactyla	Horse	*Equus caballus*	Chromosome 4: 36,788,011-36,792,765 ^a^	SAMD9L_Eqca
Primates	Common chimpanzee	*Pan troglodytes*	Chromosome 7: 92,759,911-92,778,202 ^a^	SAMD9L_Patr
	Common marmoset	*Callithrix jacchus*	Chromosome 8: 54,405,622-54,420,907 ^a^	SAMD9L_Caja
	Human	*Homo sapiens*	Chromosome 7: 92,759,368-92,777,682 ^a^	SAMD9L_Hosa
	Northern white-cheeked gibbon	*Nomascus leucogenys*	SuperContig GL397261.1: 24,263,209-24,320,238 ^a^	SAMD9L_Nole
	Rhesus monkey	*Macaca mulatta*	Chromosome 3: 124,099,607-124,117,554 ^a^	SAMD9L_Mamu
	Sumatran orangutan	*Pongo abelii*	Chromosome 7: 83,003,315-83,008,287 ^a^	SAMD9L_Poab
	Western gorilla	*Gorilla gorilla*	Chromosome 7: 90,382,062-90,397,829 ^a^	SAMD9L_Gogo
Proboscidea	African bush elephant	*Loxodonta africana*	XM_003407146.1^b^	SAMD9L_Loaf
Rodentia	Brown rat	*Rattus norvegicus*	Chromosome 4: 28,180,812-28,185,536 ^a^	SAMD9L_Rano
	Chinese hamster	*Cricetulus griseus*	XM_003496952.1 ^b^	SAMD9L_Crgr
	Domestic Guinea pig	*Cavia porcellus*	scaffold_11: 24,689,192-24,742,963 ^a^	SAMD9L_Capo
	House mouse	*Mus musculus*	Chromosome 6: 3,322,257-3,349,571^a^	SAMD9L_Mumu
Soricomorpha	Common shrew	*Sorex araneus*	scaffold 194773: 6,206-10,964 ^a^	SAMD9L_Soar

Database ID: ^a^Ensembl; ^b^NCBI GenBank.

**Figure 1:**
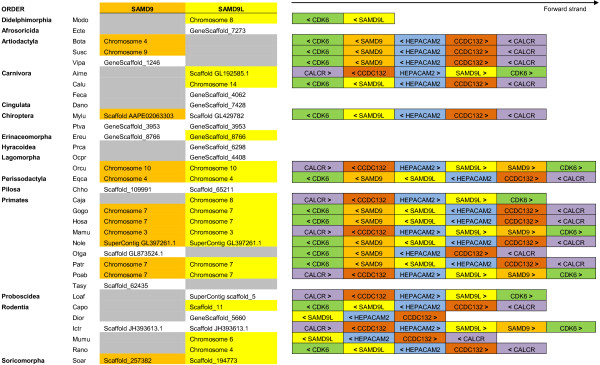
**Ensembl annotation of mammalian *SAMD9 *and *SAMD9L *genes, and neighboring genes.** Ensembl annotation of the available mammalian *SAMD9* and *SAMD9L* genes, both complete and incomplete coding region sequences, are represented. Complete sequences for both genes are highlighted (dark yellow for *SAMD9* and light yellow for *SAMD9L*). Other *SAMD9* and *SAMD9L* genes are already annotated in Ensembl, but at the time of these analyses were still incomplete, corresponding to the non-highlighted locations in the figure and were excluded from posterior analyses. Ensembl species by order of appearance: Modo - Opossum; Ecte - Lesser hedgehog tenrec; Bota - Cow; Susc - Pig; Vipa - Alpaca; Aime - Panda; Calu - Dog; Feca - Cat; Dano - Armadillo; Mylu - Microbat; Ptva - Megabat; Ereu - Hedgehog; Prca - Hyrax; Ocpr - Pika; Orcu - Rabbit; Eqca - Horse; Chho - Sloth; Caja - Marmoset; Gogo - Gorilla*;* Hosa - Human; Mamu - Macaque; Nole - Gibbon; Otga - Bushbaby; Patr - Chimpanzee; Poab - Orangutan; Tasy - Tarsier; Loaf - Elephant; Capo - Guinea pig; Dior - Kangaroo rat; Ictr - Squirrel; Mumu - Mouse; Rano - Rat; Soar - Shrew. To access the complete species name, the list of abbreviations should be consulted. Based on human chromosome 7 mapping, *SAMD9* and *SAMD9L* neighboring genes were identified and represented (*CDK6*, *HEPACAM2*, *CCDC132* and *CALCR*). Using Ensembl database, the same search was performed for the remaining species and the identified genes are represented under the forward strain arrow. The represented genes are, in most cases, the immediate neighboring genes, while for a reduced number of species some other genes are located in the same region, but were excluded for this purpose. “<” symbol: gene located on the reverse strand; “>” symbol: gene located on the forward strand.

Besides the complete *SAMD9* and *SAMD9L* coding sequences, several other non-complete *SAMD9* and *SAMD9L* mammalian genes, including full length mRNA-derived transcripts with many still-undetermined nucleotides (for example, the large flying fox or the west European hedgehog *SAMD9* coding sequences, or the American pika *SAMD9L* sequence) or partial gene sequences (for example, the Ord’s kangaroo rat *SAMD9L* or the Hoffmann’s two-toed sloth *SAMD9* genes), have been already identified and annotated in Ensembl database. However, these incomplete sequences were not used in the phylogenetic and selection analyses performed in this study. Both the complete and the non-complete *SAMD9* and *SAMD9L* genes annotated in Ensembl are represented in Figure 1, allowing a broader view into this gene family distribution within the mammalian context.

Special reference has to be made to two particular complete sequences that were included in our evolutionary analyses: the northern white-cheeked gibbon (Nole) *SAMD9* and the domestic dog (Calu) *SAMD9L*. The northern white-cheeked gibbon has no *SAMD9* gene currently annotated in Ensembl. However, by comparing *SAMD9* sequences of other primates to the gibbon genome in Ensembl using BLAST analysis, we obtained a perfect match with a neighboring designated pseudogene of *SAMD9L*. Despite this biotype classification, we could not exclude this SAMD9 sequence from being considered as a *bona fide* gibbon *SAMD9* gene. Regarding the domestic dog *SAMD9L*, this gene is present in NCBI and is annotated in Ensembl, but in this latter database the sequence was missing seventy-four nucleotides when compared to the sequence in NCBI. Thus, for the subsequent analyses we used only the sequence from NCBI. It should also be noted that, despite not being annotated in Ensembl, an incomplete *SAMD9* sequence for the domestic dog is available in NCBI. However, when the NCBI sequence (XM_003639470.1) was analyzed by BLAST, it possessed 99 to 100% identity with a non-annotated region of chromosome 14. Since it is a non-complete nucleotide sequence, it was not used further for the study reported here.

When *SAMD9* and *SAMD9L* were mapped in human chromosome 7, orthologous counterparts of both genes were identified in the chimpanzee (Patr), dog (Calu) and rat (Rano), but in the house mouse (Mumu) genome there was only a single genetic correspondence to the *SAMD9L* open reading frame in chromosome 6 [1]. From what is currently available in Ensembl database, the absence of *SAMD9* for the house mouse (Mumu) is confirmed. We checked the other available rodents to confirm the presence or absence of *SAMD9* in this specific lineage. In Ensembl there is a single *SAMD9* annotation for the thirteen-lined ground squirrel (Ictr). In addition, what appear to be intact *SAMD9* genes have been deposited in NCBI database for the brown rat (Rano), the Chinese hamster (Crgr) and the domestic Guinea pig (Capo). On the other hand, like the house mouse (Mumu), the Ord’s kangaroo rat (Dior) does not have *SAMD9* gene annotated in Ensembl database.

### Complete mammalian *SAMD9* and *SAMD9L* gene sequences: recombination and phylogenetic analyses

The complete nucleotide coding sequences from *SAMD9* and *SAMD9L* were aligned together (*SAMD9* + *SAMD9L*) and translated into deduced protein sequences (Additional file 1: Figure S1). Before further phylogenetic analyses, we used the software GARD [13,14] to look for any evidence of recombination in the alignment. Three breakpoints were identified, but only one was strongly supported by the Kishino-Hasegawa (KH) test (Additional file 2: Table S1), which should result in the estimation of a phylogenetic tree for each segment. However, since the breakpoint was located on nucleotide 4755, the genomic segment to the right of the breakpoint was only composed of 150 nucleotides.

**Figure 2:**
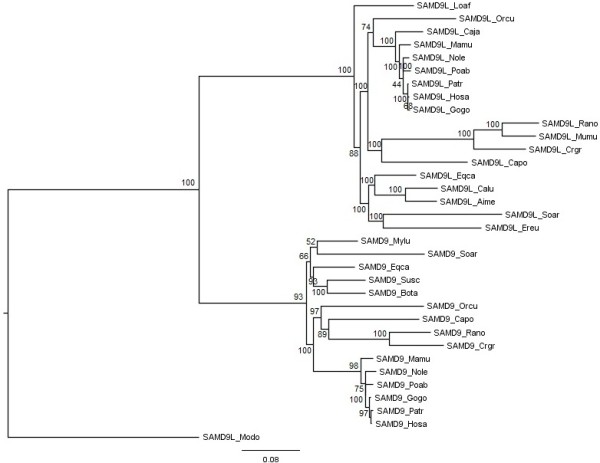
**Mammalian *SAMD9 *and *SAMD9L *genes estimated Maximum Likelihood tree.** For the mammalian *SAMD9* and *SAMD9L* genes alignment and after GARD analysis [13,14], a significant recombination breakpoint was detected (nucleotide position 4755) defining a left and a right segment. A phylogenetic tree was estimated for each segment using the Maximum Likelihood (ML) method. However, the resulting tree from the right segment presented weakly supported nodes and was discarded. On the other hand, the left segment with 4755 nucleotides was used to reconstruct a ML phylogenetic tree under the GTR+I+G nucleotide substitution model. The analyses were performed with 1,000,000 generations and 1,000 bootstrap searches. The bootstrap values are indicated on the branches. The abbreviations correspond to the following species common names: Aime - Giant panda; Bota - Cow; Caja - Common marmoset; Calu - Domestic dog; Capo - Domestic Guinea pig; Crgr - Chinese hamster; Eqca - Horse; Ereu - West European hedgehog; Gogo - Western gorilla; Hosa - Human; Loaf - African bush elephant; Mamu - Rhesus monkey; Modo - Grey short-tailed opossum; Mumu - House mouse; Mylu - Little brown myotis; Nole - Northern white-cheeked gibbon; Orcu - European rabbit; Patr - Common chimpanzee; Poab - Sumatran orangutan; Rano - Brown rat; Soar - Common shrew ; Susc - Pig. To access the species scientific names, the list of abbreviations should be consulted.

A Maximum Likelihood (ML) tree was estimated for the smallest genetic segment (not shown), but the nodes were weakly supported by low bootstrap values. Therefore, only the large segment with 4755 nucleotides was used to reconstruct a ML phylogenetic tree under the GTR+I+G nucleotide substitution model. The resulting tree is represented in Figure 2. Another ML phylogenetic tree was estimated, but without testing recombination, to compare differences in the tree topologies. The model used was again the GTR+I+G and resulted in a tree (Additional file 3: Figure S2) with a similar overall topology to the gene segment containing 4755 nucleotides.

In the estimated ML phylogenetic tree (Figure 2), *SAMD9* and *SAMD9L* formed two well defined monophyletic groups, and within each clade we observed a concordant topology with the accepted evolutionary relationships of eutherian mammals [15] (Additional file 4: Figure S3). Interestingly, the marsupial grey short-tailed opossum (Modo) *SAMD9L* represented a highly divergent outgroup, even from the remaining *SAMD9L* species.

### A gene duplication event after the split of marsupial and placental mammals originated *SAMD9*/*SAMD9L* gene family

It has been previously suggested that *SAMD9* and its paralogous *SAMD9L* may have originated from a common ancestor by a gene duplication event [1]. In our study, the ML tree (Figure 2) topology supports this view. However, the opossum (Modo) gene annotated as *SAMD9L* in NCBI database (XM_001378475.1) does not cluster in the placental mammal *SAMD9L* group. In fact, the opossum sequence can be recognized as being in a basal position. Two highly supported eutherian monophyletic clades in the ML tree, one corresponding to all *SAMD9* genes and the other one to all *SAMD9L* genes, were observed. The most likely evolutionary scenario can be described as following: an ancestral gene is present before the separation of marsupial from placental mammals in the common ancestor that originated the extant *SAMD9L* gene in the marsupial opossum (Modo) and the ancestral gene of placental *SAMD9*/*SAMD9L* gene family. Later, in placental mammals, this ancestral gene suffered an event of gene duplication resulting in the contemporary *SAMD9* and *SAMD9L* genes.

The conservation of similar arrangement of genes in the same relative locations on the chromosomes of different species, denominated as shared synteny, can indicate the existence of a common ancestor. In Ensembl, among the mammalian species where the presence of *SAMD9* and/or *SAMD9L* has been annotated, shared synteny can be readily observed in chromosomes and ‘gene-scaffolds’. The consistent presence of the same common flanking genes (*CALCR*, *CCDC132*, *CDK6* and *HEPACAM2*) in different species supports the idea that *SAMD9* and *SAMD9L* are located in highly conserved regions throughout placental mammals’ divergence and diversification (Figure 1).

### Inference of positive selection at *SAMD9* and *SAMD9L* genes level

Placental SAMD9 and SAMD9L deduced protein sequences were aligned independently (Additional file 5: Figure S4; Additional file 6: Figure S5) and ML trees were estimated for each gene (Additional file 7: Figure S6; Additional file 8: Figure S7). Afterwards, we determined whether the *SAMD9* and *SAMD9L* genes might have been subject to positive selection pressures by comparing PAML codon-based nested models with and without positive selection using likelihood ratio tests (LRTs) [16,17]. Both comparisons of M1 (nearly neutral) versus M2 (positive selection) and M7 (beta) versus M8 (beta and ω > 1) resulted in the rejection of the null hypothesis, strongly supporting the finding of positive selection for both *SAMD9* and *SAMD9L* (<0.001; Table 2). We also used the PARRIS [18] method to detect if a proportion of sites in each gene alignment evolved under positive selection after accounting for the potentially confounding effects of recombination and synonymous site variation. Interestingly, only *SAMD9L* was found to be under selection when using this method (<0.05; Additional file 9: Table S2).

**Table 2:** ***SAMD9 *and *SAMD9L *likelihood ratio test (LRT) for four site models from PAML software**

**Hypothesis**		**LRT**		
Null Hypothesis	Alternative Hypothesis	−2ΔlnL	df	*p-*Value
Site Models				
***SAMD9***				
M1: nearly neutral	M2: positive selection	25.55	2	< 0.001***
M7: beta	M8: beta and ω > 1	77.76	2	< 0.001***
***SAMD9L***				
M1: nearly neutral	M2: positive selection	51.10	2	< 0.001***
M7: beta	M8: beta and ω > 1	97.44	2	< 0.001***

***, highly significant.

**Figure 3:**
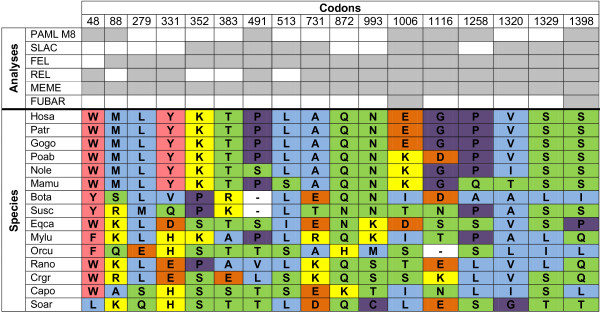
**Positively-selected SAMD9 codons and respective physicochemical properties for each mammalian species.** SAMD9 sites under positive selection identified by at least three of the six used Maximum Likelihood methods. Codons are numbered according to the SAMD9 deduced proteins alignment (Additional file 5: Figure S4). The abbreviations correspond to the following species common names: Hosa - Human; Patr - Common chimpanzee; Gogo - Western gorilla; Poab - Sumatran orangutan; Nole - Northern white-cheeked gibbon; Mamu - Rhesus monkey; Bota - Cow; Susc - Pig; Eqca - Horse; Mylu - Little brown myotis; Orcu - European rabbit; Rano - Brown rat; Crgr - Chinese hamster; Capo - Domestic Guinea pig; Soar - Common shrew. To access the species scientific names, the list of abbreviations should be consulted. The background colors represent amino acid properties: polar positive (yellow), polar negative (orange), polar neutral (green), non-polar neutral (purple), non-polar aliphatic (blue) and non-polar aromatic (pink).

**Figure 4:**
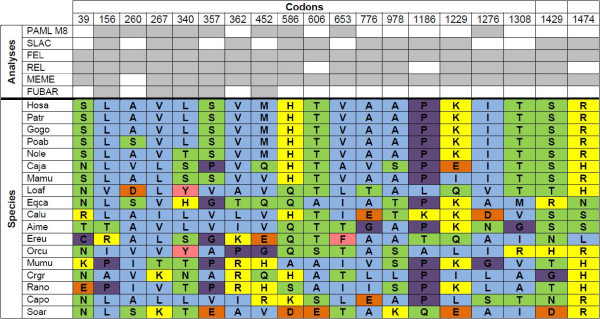
**Positively-selected SAMD9L codons and respective physicochemical properties for each mammalian species.** SAMD9L sites under positive selection identified by at least three of the six used Maximum Likelihood methods. Codons are numbered according to the SAMD9L deduced proteins alignment (Additional file 6: Figure S5). The abbreviations correspond to the following species common names: Hosa - Human; Patr - Common chimpanzee; Gogo - Western gorilla; Poab - Sumatran orangutan; Nole - Northern white-cheeked gibbon; Caja - Common marmoset; Mamu - Rhesus monkey; Loaf - African bush elephant; Eqca - Horse; Calu - Domestic dog; Aime - Giant panda; Ereu - West European hedgehog; Orcu - European rabbit; Mumu - House mouse; Crgr - Chinese hamster; Rano - Brown rat; Capo - Domestic Guinea pig; Soar - Common shrew. To access the species scientific names, the list of abbreviations should be consulted. The background colors represent amino acid properties: polar positive (yellow), polar negative (orange), polar neutral (green), non-polar neutral (purple), non-polar aliphatic (blue) and non-polar aromatic (pink).

Six different methods were used to detect sites under selection for *SAMD9* and *SAMD9L* (Additional file 10: Table S3). For PAML software, we used M8 model to detect sites under selection for *SAMD9* and *SAMD9L* phylogenetic trees, and the BEB approach was used to identify codons with a posterior probability >90%. The other five applied methods to detect sites under positive selection are available in the Datamonkey web server. In this study, we only considered a codon with evidence of selection when it was identified by at least three of the six used methods [19,20] (Additional file 10: Table S3). Seventeen sites for SAMD9 and nineteen sites for SAMD9L were identified as candidates for sites under positive selection (Figure 3 and 4; Additional file 10: Table S3).

Amino acid substitutions can be either conservative or radical, depending on whether they lead to a change in a certain physicochemical property [21]. For the codons identified as being under selection, we investigated the alterations of charge and polarity between mammalian taxa. For SAMD9 all the detected codons (Figure 3) exhibited at least one physicochemical alteration across species and a maximum of five different combinations of properties were identified for codon 331. Primate species SAMD9 amino acid changes were quite conservative, since eleven codons exhibited the same amino acid. Despite the low number of species available for Artiodactyla and Rodentia, we verified in each order a great number of amino acid physicochemical alterations *per* codon in the SAMD9 genes. In addition, all SAMD9L codons under presumptive selection (Figure 4) exhibited physicochemical alterations across taxa and at least three properties were represented in each codon. A maximum of five different physicochemical properties were identified for codon position 452. In Primates, amino acid substitutions in SAMD9L were once again quite conservative, given that thirteen positions kept the same physicochemical properties even when amino acid substitutions happened. On the contrary, among the four Rodentia species, only three positions in SAMD9L presented the same physicochemical properties, but just one was in fact the same amino acid.

To detect whether some sites along particular *SAMD9* and *SAMD9L* lineages were under positive selection, we employed branch-site Model A (Table 3). On the *SAMD9* phylogenetic tree we identified six branches (foreground branches) with *ω* ratio greater than 1, but only the common shrew (Soar) branch had a statistical significant LRT (<0.01). *SAMD9L* branch-site analysis revealed a total of twelve branches with *ω* ratio greater than 1, yet only four of those branches presented a statistical significant LRT. Both the Sumatran orangutan (Poab) and the domestic Guinea pig (Capo) branches had statistical significance <0.05, while the west European hedgehog (Ereu) and the common shrew (Soar) branches had statistical significance <0.01.

**Table 3:** ***SAMD9 *and *SAMD9L *parameter estimates and likelihood ratio test (LRT) for branch-site model A (PAML)**

**Branch-site Model A**		**LRT**			
**Foreground branches **^**a**^	**Parameter estimates**	**−2ΔlnL**^**b**^	**df **^**c**^	***p*-Value**	**Positively selected sites **^**d**^
***SAMD9***					
Gogo	*p*_*0*_ = 0.693 *p*_*1*_ = 0.291 *p*_*2a*_ = 0.011 *p*_*2b*_ = 0.005 *ω*_*0*_ = 0.096 *ω*_*1*_ = 1.000 *ω*_*2*_ = **6.192**	0.20	1	n.s.	none
Poab	*p*_*0*_ = 0.701 *p*_*1*_ = 0.293 *p*_*2a*_ = 0.004 *p*_*2b*_ = 0.002 *ω*_*0*_ = 0.096 *ω*_*1*_ = 1.000 *ω*_*2*_ = **21.339**	3.65	1	n.s.	none
Nole	*p*_*0*_ = 0.692 *p*_*1*_ = 0.291 *p*_*2a*_ = 0.012 *p*_*2b*_ = 0.005 *ω*_*0*_ = 0.095 *ω*_*1*_ = 1.000 *ω*_*2*_ = **2.555**	0.12	1	n.s.	none
Orcu	*p*_*0*_ = 0.696 *p*_*1*_ = 0.290 *p*_*2a*_ = 0.010 *p*_*2b*_ = 0.004 *ω*_*0*_ = 0.094 *ω*_*1*_ = 1.000 *ω*_*2*_ = **4.818**	3.30	1	n.s.	none
Capo	*p*_*0*_ = 0.702 *p*_*1*_ = 0.292 *p*_*2a*_ = 0.005 *p*_*2b*_ = 0.002 *ω*_*0*_ = 0.096 *ω*_*1*_ = 1.000 *ω*_*2*_ = **5.728**	1.25	1	n.s.	none
Soar	*p*_*0*_ = 0.696 *p*_*1*_ = 0.286 *p*_*2a*_ = 0.013 *p*_*2b*_ = 0.005 *ω*_*0*_ = 0.094 *ω*_*1*_ = 1.000 *ω*_*2*_ = **8.165**	**9.56**	1	**< 0.01**	288, 572
***SAMD9L***					
Poab	*p*_*0*_ = 0.729 *p*_*1*_ = 0.270 *p*_*2a*_ = 0.001 *p*_*2b*_ = 0.000 *ω*_*0*_ = 0.139 *ω*_*1*_ = 1.000 *ω*_*2*_ = **409.279**	**6.59**	1	**< 0.05**	888
Caja	*p*_*0*_ = 0.714 *p*_*1*_ = 0.263 *p*_*2a*_ = 0.016 *p*_*2b*_ = 0.006 *ω*_*0*_ = 0.138 *ω*_*1*_ = 1.000 *ω*_*2*_ = **3.169**	1.12	1	n.s.	none
Mamu	*p*_*0*_ = 0.727 *p*_*1*_ = 0.268 *p*_*2a*_ = 0.004 *p*_*2b*_ = 0.001 *ω*_*0*_ = 0.140 *ω*_*1*_ = 1.000 *ω*_*2*_ = **11.372**	1.22	1	n.s.	none
Loaf	*p*_*0*_ = 0.717 *p*_*1*_ = 0.262 *p*_*2a*_ = 0.015 *p*_*2b*_ = 0.006 *ω*_*0*_ = 0.139 *ω*_*1*_ = 1.000 *ω*_*2*_ = **2.244**	0.80	1	n.s.	none
Calu	*p*_*0*_ = 0.730 *p*_*1*_ = 0.269 *p*_*2a*_ = 0.013 *p*_*2b*_ = 0.000 *ω*_*0*_ = 0.140 *ω*_*1*_ = 1.000 *ω*_*2*_ = **20.273**	0.52	1	n.s.	none
Aime	*p*_*0*_ = 0.728 *p*_*1*_ = 0.269 *p*_*2a*_ = 0.003 *p*_*2b*_ = 0.001 *ω*_*0*_ = 0.139 *ω*_*1*_ = 1.000 *ω*_*2*_ = **16.318**	2.51	1	n.s.	none
Ereu	*p*_*0*_ = 0.725 *p*_*1*_ = 0.266 *p*_*2a*_ = 0.006 *p*_*2b*_ = 0.002 *ω*_*0*_ = 0.139 *ω*_*1*_ = 1.000 *ω*_*2*_ = **998.998**	**7.45**	1	**< 0.01**	none
Mumu	*p*_*0*_ = 0.716 *p*_*1*_ = 0.264 *p*_*2a*_ = 0.015 *p*_*2b*_ = 0.005 *ω*_*0*_ = 0.138 *ω*_*1*_ = 1.000 *ω*_*2*_ = **3.755**	0.34	1	n.s.	none
Crgr	*p*_*0*_ = 0.728 *p*_*1*_ = 0.268 *p*_*2a*_ = 0.003 *p*_*2b*_ = 0.001 *ω*_*0*_ = 0.139 *ω*_*1*_ = 1.000 *ω*_*2*_ = **38.672**	2.62	1	n.s.	none
Rano	*p*_*0*_ = 0.727 *p*_*1*_ = 0.268 *p*_*2a*_ = 0.004 *p*_*2b*_ = 0.001 *ω*_*0*_ = 0.139 *ω*_*1*_ = 1.000 *ω*_*2*_ = **11.843**	2.14	1	n.s.	none
Capo	*p*_*0*_ = 0.722 *p*_*1*_ = 0.261 *p*_*2a*_ = 0.012 *p*_*2b*_ = 0.004 *ω*_*0*_ = 0.139 *ω*_*1*_ = 1.000 *ω*_*2*_ = **6.984**	**6.36**	1	**< 0.05**	861
Soar	*p*_*0*_ = 0.717 *p*_*1*_ = 0.264 *p*_*2a*_ = 0.014 *p*_*2b*_ = 0.005 *ω*_*0*_ = 0.137 *ω*_*1*_ = 1.000 *ω*_*2*_ = **7.759**	**10.49**	1	**< 0.01**	84, 1338, 1346

^a^ Species names on the foreground branches by order of appearance: Gogo - Western gorilla; Poab - Sumatran orangutan; Nole - Northern white-cheeked gibbon; Orcu - European rabbit; Capo - Domestic Guinea pig; Soar - Common shrew; Caja - Common marmoset; Mamu - Rhesus monkey; Loaf - African bush elephant; Calu - Domestic dog; Aime - Giant panda; Ereu - West European hedgehog; Mumu - House mouse; Crgr - Chinese hamster; Rano - Brown rat.

^b^ -2ΔlnL: likelihood ratio test (LRT) to detect positive selection.

^c^ df: degrees of freedom.

^d^ Positively selected sites: posterior probabilities > 90% in the BEB (Bayes Empirical Bayes) analyses.

### Inference of positive selection at SAMD9 and SAMD9L deduced proteins level

The evaluation of destabilizing radical changes that may occur in specific regions of proteins should complement the information obtained from positive selection analyses at the gene level. Using TreeSAAP software, it is possible to estimate, from a phylogenetic tree, the amino acid properties under selection from the thirty-one available in the software [22] (see Methods section for full list of the thirty-one properties).

**Figure 5:**
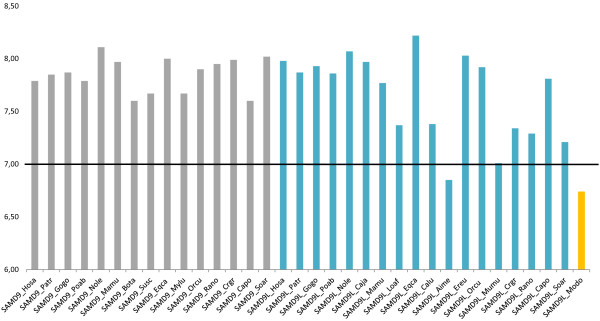
**Mammalian SAMD9 and SAMD9L deduced proteins isoelectric points (pI).** The grey bars correspond to the SAMD9 deduced proteins pI, the blue bars to the SAMD9L deduced proteins pI and the yellow bar to the opossum (Modo) SAMD9L deduced protein pI. The abbreviations correspond to the following species common names: Hosa - Human; Patr - Common chimpanzee; Gogo - Western gorilla; Poab - Sumatran orangutan; Nole - Northern white-cheeked gibbon; Mamu - Rhesus monkey; Bota - Cow; Susc - Pig; Eqca - Horse; Mylu - Little brown myotis; Orcu - European rabbit; Rano - Brown rat; Crgr - Chinese hamster; Capo - Domestic Guinea pig; Soar - Common shrew; Caja - Common marmoset; Loaf - African bush elephant; Calu - Domestic dog; Aime - Giant panda; Ereu - West European hedgehog; Mumu - House mouse; Modo - Grey short-tailed opossum. To access the species scientific names, the list of abbreviations should be consulted.

For both SAMD9 and SAMD9L phylogenetic trees, the two amino acid properties with the most radical value (category 8) denoting positive destabilizing selection were the isoelectric point (pI) and the equilibrium constant (ionization of COOH) (Additional file 11: Table S4). When comparing the pI values among species for each protein, we observed a high variability across them, especially for SAMD9L taxa (Figure 5). For SAMD9 proteins, both the cow (Bota) and the domestic Guinea pig (Capo) exhibited the lowest pI (7.60), while a pI of 8.11 for the northern white-cheeked gibbon was the highest observed in SAMD9 proteins. SAMD9L proteins from placental mammals exhibited a larger range for the pI values with the giant panda (Aime) presenting the lowest pI (6.85) and the horse (Eqca) exhibiting the highest pI (8.22). Interestingly, the marsupial grey short-tailed opossum SAMD9L deduced protein presented the lowest pI (6.74) of all. The differences in the pI, and especially in SAMD9L proteins, may cause dramatic effects on proteins folding, since those changes are caused by significant differences in the polarity of the amino acids that compose the proteins. Besides the pI and equilibrium constant, SAMD9 presented two other properties under strong positive destabilizing selection, while five more properties were identified as being under positive destabilizing selection for the SAMD9L alignment (Additional file 11: Table S4).

**Figure 6:**
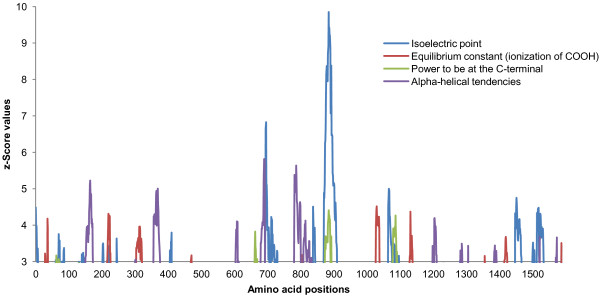
**Sliding window for SAMD9 amino acid properties under positive selection.** SAMD9 amino acid properties under destabilizing selection with significant z-Score values (>3.09).

**Figure 7:**
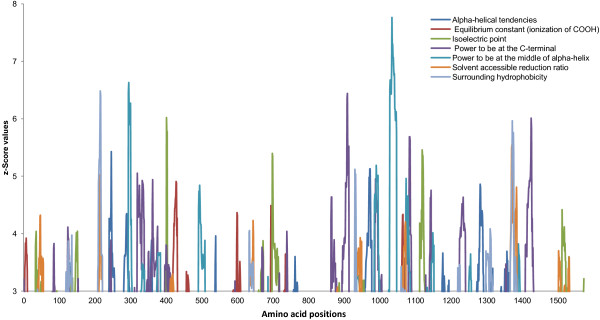
**Sliding window for SAMD9L amino acid properties under positive selection.** SAMD9L amino acid properties under destabilizing selection with significant z-Score values (>3.09).

Regarding the SAMD9 sliding window, the four amino acid properties with significant z-Score values (>3.09) were evenly distributed along the SAMD9 proteins alignment (Figure 6). However, a superior concentration of higher z-Score values was observed in the region between amino acid 660 and 910, specifically for the pI. The SAMD9L sliding window showed a dense pattern for the seven amino acid properties under destabilizing selection (Figure 7). Yet, two regions of SAMD9L proteins alignment presented an even larger density of properties and the highest z-Score values for some of those properties: amino acid range of 208–431 and the range of 863–1430.

## Discussion

From a previous study, *SAMD9* and its paralogue *SAMD9L* have been identified in a variety of species, namely in human, chimpanzee, dog and rat. However, in the house mouse (*Mus musculus*, Mumu) genome, *SAMD9* was uniquely lost [1]. The same study indicated the absence of both genes in chicken, frog and all currently sequenced fish species, suggesting that the *SAMD9*/*SAMD9L* genes originating event had occurred after the mammalian radiation. One of our goals was to intensify the identification of *SAMD9* and *SAMD9L* within different mammalian genomes and also verify whether the loss of mouse *SAMD9* was a unique event restricted to this taxon.

Despite the great number of morphological, molecular and phylogenetic studies for the order Rodentia, controversies relating to the divergence times between its major suborders still persist [23]. In a recent study on rodent evolution [24] some internal rodent branches have been resolved, where three main groups in the phylogenetic tree were supported: the Mouse-related clade, Ctenohystrica clade and the Squirrel-related clade. A scenario has been proposed where the pre-Squirrel-related clade diverged early from the common ancestor followed by a later separation of the pre-Mouse-related and pre-Ctenohystrica clade [24]. We gathered sequences for one or both *SAMD9* and *SAMD9L* genes for species representative of the three clades. The two genes were present in the thirteen-lined ground squirrel (Squirrel-related clade), the domestic Guinea pig (Ctenohystrica clade), the Chinese hamster and the brown rat (Mouse-related clade). Together with the absence of *SAMD9* in the house mouse genome, the Ord’s kangaroo rat (Mouse-related clade) also did not have this gene annotated in Ensembl. With the apparent region synteny for the Ord’s kangaroo rat when compared to the other mammals, this absence might just be the case of a genome still to be completely annotated, leaving the house mouse as the only rodent taxon that has lost *SAMD9*, at least from the currently available genomic sequence database.

A great number of the available mammalian genomes are still not completely annotated. Therefore, we made no assumptions regarding *SAMD9* and *SAMD9L* for those species. Nevertheless, we observed that the fairly well annotated cow and pig genomes (Order Artiodactyla) had no matches or annotations for *SAMD9L*. This information together with the absence of *SAMD9* in the house mouse and the already suggested origin of both genes from a common ancestor by ancient gene duplication [1] led us to the following hypothesis: in some lineages the presence of both genes might be costly for the genome, resulting in the loss of one of the genes that functionally would be overcome by the remaining paralogue. Although these observations support the potential existence of certain gene redundancy between *SAMD9* and *SAMD9L*, we also note the almost nonexistent recombination between them, despite the proximity in the location of these two genes in the genomes of all the annotated mammalian species. This genetic isolation of the two paralogues does not support the existence of functional redundancy between *SAMD9* and *SAMD9L*. These apparent contradictory hypotheses have to be confirmed with the conduction of functional studies in different species.

With all the available mammalian sequences collected for both *SAMD9* and *SAMD9L* genes, the performed phylogenetic study resulted in a tree with a well-defined monophyletic group *per* gene gathering solely placental mammals and a single outgroup, the marsupial grey short-tailed opossum. This supported the speculative hypothesis of *SAMD9* and *SAMD9L* resulting from a gene duplication event, more precisely, after the divergence of Marsupialia from Placentalia 147.7 Mya [25]. Despite the common ancestor, when testing for the occurrence of potential positive selection acting at the gene and protein levels, we concluded that SAMD9L is under stronger selection than SAMD9. This is supported by the fact that a higher number of sites at the gene level and of specific lineages were positively selected in SAMD9L than SAMD9. Besides, a greater number of amino acid properties were under selection at the deduced protein level of SAMD9L than SAMD9.

When we examined the amino acid substitutions and changes on physicochemical properties for sites under selection, it was clear, for both proteins, that members of the Rodentia order presented the highest number of divergent alterations for the same codons compared to other mammalian orders. Since it is known that in many proteins the amino acid substitutions caused by positive selection are not random [21,26], for instances the Primate APOBEC3G residues involved in HIV-1 Vif interaction [27], we hypothesize that any occurring alteration in rodents or even in other lineages may be the result of consistent arms race between the host and a pathogen stressor. This could be a significant observation, given that anti-viral properties have been already assigned to human SAMD9 in cultured human cells. Specifically, a unique viral gene product, M062 of myxoma virus, was found to antagonize the anti-viral properties of SAMD9 protein in order to permit the replication of this virus in cultured human cells [8].

Considering the mammalian species included in this study, selection analyses performed on *SAMD9* and/or *SAMD9L* genes for each species individually one may have different results from the obtained in our work, since recombination rates and effective population sizes are expected to differ among species. These species and population specific selection analyses should result in the identification of sites under selection in *SAMD9* and/or *SAMD9L* genes that can be used in genetic population studies by determining parameters like allele and genotype frequencies, and F_ST_ and nucleotide diversity values. This contributes to the definition of genotypes that might be favorable or not, for example, to the defence against certain pathogens.

Human SAMD9 and SAMD9L have solely one defined domain, the Sterile Alpha Motif (SAM), a module of about 70 amino acid residues long [28], specifically 65 amino acids and 66 in SAMD9 and SAMD9L, respectively. SAM domains, one of the most common protein domains found in eukaryotic cells, are protein-protein interaction modules that perform a large number of different functions [29,30] and are not easily categorized. Indeed, different SAM domains can self-associate, bind to other SAM domains and/or to non-SAM proteins, and even interact with RNA, DNA or lipids [30]. Because of the great variety of known functions, the presence of a SAM domain does not necessarily involve a specific function or pathway, but an array of possible functions. For both human SAMD9 and SAMD9L, no function has yet been assigned to their SAM domains, but for SAMD9 the ability to form SAM polymers has been suggested [31]. From our evolutionary study on both proteins, none of the identified sites or amino acid properties under positive selection overlapped with the deduced SAM domains, demonstrating a high level of conservation among the mammalian species.

## Conclusions

Since the origin and evolution of the *SAMD9* and *SAMD9L* genes were first reported, a great number of mammalian genomes have been sequenced, allowing now a more detailed view into the evolutionary history of both genes. Our study supports the previously suggested origin of *SAMD9* and *SAMD9L* from a mammalian ancestral duplication event. Specifically, according to the results from our study, this event occurred after the divergence of Marsupialia from Placentalia. When considering the mostly complete mammalian genomes collected for this study, the apparent loss of *SAMD9* or *SAMD9L* in some species led us to propose that some overlapping functional redundancy exists between the two proteins, despite the almost nonexistent recombination between the two closely located genes from other species. From the positive selection analyses performed, both at gene and protein levels, we demonstrate that SAMD9 and SAMD9L continue to be under long term selective pressure, with even stronger evidence for positive selection in SAMD9L.

Both *SAMD9* and *SAMD9L* genes are upregulated by type I interferon, a classic feature associated with many innate pathogen-response genes called interferon-stimulated genes (ISGs). Indeed, human SAMD9 has already been shown to be a functional inhibitor for at least one viral pathogen, a poxvirus called myxoma virus, that expresses a specific viral inhibitor (M062) that counteracts the anti-viral properties of SAMD9 [8]. Our results suggest that at least the SAMD9 genes may have been under sustained selection pressure exerted by viral pathogens.

Our work is the first complete study to investigate the evolutionary history of mammalian SAMD9 and SAMD9L.

## Methods

### SAMD9 and SAMD9L nucleotide and protein sequences

All the available mammalian *SAMD9* and *SAMD9L* genes coding sequences used in the phylogenetic and positive selection analyses were retrieved from NCBI (http://www.ncbi.nlm.nih.gov) and Ensembl (http://www.ensembl.org/index.html) databases. Next, sequences were aligned with ClustalW [32] implemented in BioEdit v7.0.9 [33], followed by visual inspection. Nucleotide sequences translation into protein sequences was performed using also BioEdit.

*SAMD9* and *SAMD9L* genes coding sequences were collected for fifteen and nineteen species, respectively. Based on the Mammal Species of the World database classification (http://www.bucknell.edu/msw3/), representative species of mammalian infraclasses Metatheria (Order Didelphimorphia) and Eutheria (Order Artiodactyla, Carnivora, Chiroptera, Erinaceomorpha, Lagomorpha, Perissodactyla, Primates, Proboscidea, Rodentia and Soricomorpha) were included in this study. Table 1 summarizes the species collected for each gene and their respective accession numbers.

The isoeletric point (pI) of SAMD9 and SAMD9L deduced proteins for different species was estimated using DAMBE (Data Analysis and Molecular Biology and Evolution) [34].

### Recombination and phylogenetic analyses

Recombination can mislead phylogenetic and positive selection analyses [35], and particularly for *SAMD9* and *SAMD9L*, the genes close location (~12 kb in human genome, for example) might increase the probability of recombination to occur. Therefore, we first performed recombination testing on placental *SAMD9* and *SAMD9L* nucleotide sequences alignments, and also on the alignment of both genes together (*SAMD9* + *SAMD9L*). The software GARD (Genetic Algorithm for Recombination Detection) [13,14], implemented in the Datamonkey web server [36], was used to detect possible recombination breakpoints.

For *SAMD9* and *SAMD9L* genes alignments no significant breakpoints were detected while using GARD, thus the complete alignments were used to establish each gene phylogeny. As indicated by the Akaike Information Criterion (AIC) implemented in jModelTest v0.1.1 [37], the nucleotide substitution model TVM+G was used for *SAMD9* tree estimation, while the GTR+G model was the consensus model selected for *SAMD9L* phylogenetic tree construction. On the other hand, a significant breakpoint was detected when running GARD for the *SAMD9*+*SAMD9L* alignment and a phylogenetic tree was estimated for each segment. For the left segment, the AIC in jModelTest indicated GTR+I+G as the best-fit nucleotide substitution model, whereas for the right segment the TPM2uf+G model was indicated as the best for the tree estimation. Also, for the *SAMD9*+*SAMD9L* alignment, a phylogenetic tree was estimated without testing recombination. In this case, the jModelTest AIC estimated GTR+I+G model as the best-fit nucleotide substitution model.

To establish mammalian phylogeny for *SAMD9*, *SAMD9L* and *SAMD9*+*SAMD9L*, based on nucleotide sequences, the Maximum Likelihood (ML) method implemented on GARLI v2.0 (Genetic Algorithm for Rapid Likelihood Inference) was used [38]. The analyses were performed with 1,000,000 generations and 1,000 bootstrap searches. ML trees were displayed using FigTree v1.3.1 (http://tree.bio.ed.ac.uk/).

### Codon-based analyses of positive selection

A useful measurement for identifying adaptive protein evolution is the nonsynonymous (*d*_*N*_)/synonymous substitution (*d*_*S*_) rate (*ω* = *d*_*N*_/*d*_*S*_), where values of *ω* = 1, < 1, and > 1 indicate neutral selection, negative selection, and positive selection, respectively [39,40]. Naturally, and due to protein structural and functional constraints, *ω* is expected to be close to 0 and full protein analysis rarely detects positive selection [41]. As a result, several methods, based on models of codon substitution, have been developed to detect adaptive evolution (positive selection) at individual sites in a background of negative selection [42,43]. We employed six different methods to detect sites under selection, and based on the methodology adopted by several authors [19,20] only codons identified by at least three of the six used methods were considered to be under positive selection.

To detect selection based on the ratio *ω* and at the gene-level, for both *SAMD9* and *SAMD9L*, PAML v4.4 (Phylogenetic Analysis by Maximum Likelihood) [16,17] was used and the codon frequency model F3x4 was fitted to both alignments. In the site-specific models that allow the ratio *ω* to vary among codons, we performed Likelihood Ratio Tests (LRTs) with 2 degrees of freedom to compare the following models (*NS sites*): M1 (nearly neutral) with M2 (selection) and M7 (neutral, *β* distribution of *ω* < 1) with M8 (selection, *β* distribution of *ω* > 1). A significant LRT demonstrates that the selection model fits better than the neutral model [42,43]. For model M8, a Bayes empirical Bayes (BEB) approach was employed to detect codons with a posterior probability >90% of being under selection [44]. Also the branch-site model A was performed for testing positive selection on individual sites along a specific lineage, called foreground branch, where the other lineages are background branches. In branch-site model A, three *ω* ratios are assumed for foreground (0 < *ω*_*0*_ *<* 1, *ω*_*1*_ = 1, *ω*_*2*_ > 1) and two *ω* ratios for background (0 < *ω*_*0*_ *<* 1, *ω*_*1*_ = 1). The null model is the same as model A, but *ω*_*2*_ = 1 is fixed. We also used BEB approach to calculate the posterior probability of a specific codon site and to identify those most likely to be under positive selection (posterior probability >90%) [44].

Both *SAMD9* and *SAMD9L* genes were also analyzed using HyPhy software implemented in the Datamonkey web server [36]. Datamonkey includes three classic ML methods to detect sites under selection: the Single Likelihood Ancestor Counting (SLAC) model, the Fixed Effect Likelihood (FEL) model and the Random Effect Likelihood (REL) model [45]. Besides these three methods, two other recently developed and implemented in the Datamonkey web server were applied to our dataset: the Mixed Effects Model of Evolution (MEME) that allows the distribution of *ω* to vary from site to site and also from branch to branch at a site, being capable of identifying both episodic and pervasive positive selection [46], and the Fast Unbiased Bayesian AppRoximation (FUBAR) method that can detect positive selection under a model faster than the existing fixed effects likelihood models through the introduction of an ultra-fast Markov chain Monte Carlo (MCMC) routine and that allows to visualize Bayesian inference for each site [47]. All these methods were run using the best model chosen by AIC on a defined Neighbor-Joining (NJ) phylogenetic tree after running GARD to detect recombination. To avoid a high false-positive rate, due to the reduced number of sequences [45], sites with *p-*values <0.1 for SLAC, FEL and MEME models, Bayes Factor >50 for REL model and a posterior probability >0.90 for FUBAR were accepted as candidates for selection.

From the HyPhy software available on the Datamonkey web server, we also run the PARRIS method used to detect if a proportion of sites in the alignment evolve with *d*_*N*_*/d*_*S*_ > 1 and that accounts for synonymous rate variation and recombination [18].

### Amino acid-based analyses of positive selection

By using TreeSAAP v3.2 (Selection of Amino Acid Properties based on Phylogenetic Trees) [22] it was possible to detect selection signatures at the amino acid level, more specifically, positively selected amino acid properties that result in radical structural and functional changes in local regions of the protein (destabilization). Properties that fell into categories 6 through 8 (the most radical values denoting positive destabilizing selection), presented z-score values of 3.09 and higher, and with a probability value of 0.001 were plotted in a sliding window (length = 20).

Thirty-one amino acid properties were evaluated across SAMD9 and SAMD9L phylogenetic trees to identify protein regions that presented evidence of positive destabilization for each property. The thirty-one amino acid properties are the following: alpha-helical tendencies, average number of surrounding residues, beta-structure tendencies, bulkiness, buriedness, chromatographic index, coil tendencies, composition, compressibility, equilibrium constant (ionization of COOH), helical contact area, hydropathy, isoelectric point, long-range non-bonded energy, mean r.m.s. fluctuation displacement, molecular volume, molecular weight, normalized consensus hydrophobicity, partial specific volume, polar requirement, polarity, power to be at the C-terminal, power to be at the middle of alpha-helix, power to be at the N-terminal, refractive index, short and medium range non-bonded energy, solvent accessible reduction ratio, surrounding hydrophobicity, thermodynamic transfer hydrophobicity, total non-bonded energy and turn tendencies.

## Abbreviations

Aime: Giant panda - *Ailuropoda melanoleuca*; Bota: Cow - *Bos taurus*; Caja: Common marmoset - *Callithrix jacchus*; Calu: Domestic dog - *Canis lupus familiaris*; Capo: Domestic Guinea pig - *Cavia porcellus*; Chho: Hoffmann’s two-toed sloth - *Choloepus hoffmanni*; Crgr: Chinese hamster - *Cricetulus griseus*; Dano: Nine-banded armadillo - *Dasypus novemcinctus*; Dior: Ord’s kangaroo rat - *Dipodomys ordii*; Ecte: Lesser hedgehog tenrec - *Echinops telfairi*; Eqca: Horse - *Equus caballus*; Ereu: West European hedgehog - *Erinaceus europaeus*; Feca: Domestic cat - *Felis catus*; Gogo: Western gorilla - *Gorilla gorilla*; Hosa: Human - *Homo sapiens*; Ictr: Thirteen-lined ground squirrel - *Ictidomys tridecemlineatus*; Loaf: African bush elephant - *Loxodonta africana*; Mamu: Rhesus monkey - *Macaca mulatta*; Modo: Grey short-tailed opossum - *Monodelphis domestica*; Mumu: House mouse - *Mus musculus*; Mylu: Little brown myotis - *Myotis lucifugus*; Nole: Northern white-cheeked gibbon - *Nomascus leucogenys*; Ocpr: American pika - *Ochotona princeps*; Orcu: European rabbit - *Oryctolagus cuniculus*; Otga: Northern greater galago - *Otolemur garnettii*; Patr: Common chimpanzee - *Pan troglodytes*; Poab: Sumatran orangutan - *Pongo abelii*; Prca: Rock hyrax - *Procavia capensis*; Ptva: Large flying fox - *Pteropus vampyrus*; Rano: Brown rat - *Rattus norvegicus*; Soar: Common shrew - *Sorex araneus*; Susc: Pig - *Sus scrofa*; Tasy: Philippine tarsier - *Tarsius syrichta*; Vipa: Alpaca - *Vicugna pacos.*

## Competing interests

The authors declare that they have no competing interests.

## Authors’ contributions

ALM participated in the design of the research, performed the data analyses and drafted the manuscript. JL, GM and PJE conceived the study, designed the research and drafted the manuscript. All authors read and approved the final manuscript.

## Supplementary Material

Additional file 1: Figure S1 Mammalian SAMD9 and SAMD9L deduced protein sequences alignment. *SAMD9* and *SAMD9L* genes coding sequences were collected for fifteen and nineteen species, respectively. Sequences were aligned with ClustalW implemented in BioEdit. The abbreviations correspond to the following species common names: Hosa - Human; Patr - Common chimpanzee; Gogo - Western gorilla; Poab - Sumatran orangutan; Nole - Northern white-cheeked gibbon; Mamu - Rhesus monkey; Bota - Cow; Susc - Pig; Eqca - Horse; Mylu - Little brown myotis; Orcu - European rabbit; Rano - Brown rat; Crgr - Chinese hamster; Capo - Domestic Guinea pig; Soar - Common shrew; Caja - Common marmoset; Loaf - African bush elephant; Calu - Domestic dog; Aime - Giant panda; Ereu - West European hedgehog; Mumu - House mouse; Modo - Grey short-tailed opossum. To access the species scientific names, the list of abbreviations should be consulted. Codons are numbered according to human SAMD9 protein. “?” represents undetermined codons; “.” represents identity with the reference sequence of human SAMD9 protein. Click here for file

Additional file 2: Table S1 Detection of recombination breakpoints from *SAMD9* and *SAMD9L* genes alignment using GARD analysis. *SAMD9* and *SAMD9L* complete coding sequences were aligned together and the software GARD was used to look for any evidence of recombination. Three breakpoints were identified, but only one was strongly supported by the Kishino-Hasegawa (KH) test. Click here for file

Additional file 3: Figure S2 Mammalian *SAMD9* and *SAMD9L* genes estimated Maximum Likelihood tree without testing recombination. A phylogenetic tree was estimated for the mammalian *SAMD9* and *SAMD9L* genes alignment using the Maximum Likelihood (ML) method and under the GTR+I+G nucleotide substitution model. The analyses were performed with 1,000,000 generations and 1,000 bootstrap searches. The bootstrap values are indicated on the branches. The abbreviations correspond to the following species common names: Aime - Giant panda; Bota - Cow; Caja - Common marmoset; Calu - Domestic dog; Capo - Domestic Guinea pig; Crgr - Chinese hamster; Eqca - Horse; Ereu - West European hedgehog; Gogo - Western gorilla; Hosa - Human; Loaf - African bush elephant; Mamu - Rhesus monkey; Modo - Grey short-tailed opossum; Mumu - House mouse; Mylu - Little brown myotis; Nole - Northern white-cheeked gibbon; Orcu - European rabbit; Patr - Common chimpanzee; Poab - Sumatran orangutan; Rano - Brown rat; Soar - Common shrew ; Susc - Pig. To access the species scientific names, the list of abbreviations should be consulted. Click here for file

Additional file 4: Figure S3 Evolutionary relationships of eutherian mammals. Placental mammals’ evolutionary relationships tree retrieved and adapted from Song *et al.*[15]. Click here for file

Additional file 5: Figure S4 Mammalian SAMD9 deduced protein sequences alignment. SAMD9 deduced protein sequences from fifteen species were aligned with ClustalW implemented in BioEdit. The abbreviations correspond to the following species common names: Hosa - Human; Patr - Common chimpanzee; Gogo - Western gorilla; Poab - Sumatran orangutan; Nole - Northern white-cheeked gibbon; Mamu - Rhesus monkey; Bota - Cow; Susc - Pig; Eqca - Horse; Mylu - Little brown myotis; Orcu - European rabbit; Rano - Brown rat; Crgr - Chinese hamster; Capo - Domestic Guinea pig; Soar - Common shrew. To access the species scientific names, the list of abbreviations should be consulted. Codons are numbered according to human SAMD9 protein. “?” represents undetermined codons; “.” represents identity with the reference sequence of human SAMD9 protein. Click here for file

Additional file 6: Figure S5 Mammalian SAMD9L deduced protein sequences alignment. SAMD9L deduced protein sequences from eighteen species were aligned with ClustalW implemented in BioEdit. The abbreviations correspond to the following species common names: Hosa - Human; Patr - Common chimpanzee; Gogo - Western gorilla; Poab - Sumatran orangutan; Nole - Northern white-cheeked gibbon; Caja - Common marmoset; Mamu - Rhesus monkey; Loaf - African bush elephant; Eqca - Horse; Calu - Domestic dog; Aime - Giant panda; Ereu - West European hedgehog; Orcu - European rabbit; Mumu - House mouse; Crgr - Chinese hamster; Rano - Brown rat; Capo - Domestic Guinea pig; Soar - Common shrew. To access the species scientific names, the list of abbreviations should be consulted. Codons are numbered according to human SAMD9L protein. “?” represents undetermined codons; “.” represents identity with the reference sequence of human SAMD9L protein. Click here for file

Additional file 7: Figure S6 Mammalian *SAMD9* gene estimated Maximum Likelihood tree. The phylogenetic tree of mammalian *SAMD9* gene alignment was estimated using the Maximum Likelihood method and the nucleotide substitution model TVM+G. The analyses were performed with 1,000,000 generations and 1,000 bootstrap searches. The bootstrap values are indicated on the branches. The abbreviations correspond to the following species common names: Hosa - Human; Patr - Common chimpanzee; Gogo - Western gorilla; Poab - Sumatran orangutan; Nole - Northern white-cheeked gibbon; Mamu - Rhesus monkey; Bota - Cow; Susc - Pig; Eqca - Horse; Mylu - Little brown myotis; Orcu - European rabbit; Rano - Brown rat; Crgr - Chinese hamster; Capo - Domestic Guinea pig; Soar - Common shrew. To access the species scientific names, the list of abbreviations should be consulted. Click here for file

Additional file 8: Figure S7 Mammalian *SAMD9L* gene estimated maximum likelihood tree. The phylogenetic tree of mammalian *SAMD9L* gene alignment was estimated using the Maximum Likelihood method and the nucleotide substitution model GTR+G. The analyses were performed with 1,000,000 generations and 1,000 bootstrap searches. The bootstrap values are indicated on the branches. The abbreviations correspond to the following species common names: Hosa - Human; Patr - Common chimpanzee; Gogo - Western gorilla; Poab - Sumatran orangutan; Nole - Northern white-cheeked gibbon; Caja - Common marmoset; Mamu - Rhesus monkey; Loaf - African bush elephant; Eqca - Horse; Calu - Domestic dog; Aime - Giant panda; Ereu - West European hedgehog; Orcu - European rabbit; Mumu - House mouse; Crgr - Chinese hamster; Rano - Brown rat; Capo - Domestic Guinea pig; Soar - Common shrew. To access the species scientific names, the list of abbreviations should be consulted. Click here for file

Additional file 9: Table S2 *SAMD9* and *SAMD9L* likelihood ratio test (LRT) for PARRIS analysis from HyPhy software. Only *SAMD9L* was found to be under selection when using this specific method. Click here for file

Additional file 10: Table S3 Positively-selected codon positions in *SAMD9* and *SAMD9L* determined by six different Maximum Likelihood methods. The six methods correspond to PAML M8, SLAC, FEL, REL, MEME and FUBAR. Codons positions are numbered according to human SAMD9 and SAMD9L proteins (Additional file 5 Figure S4 and Additional file 6 Figure S5). Click here for file

Additional file 11: Table S4 SAMD9 and SAMD9L amino acid properties under positive selection determined in TreeSAAP. SAMD9 exhibited three and SAMD9L evidenced seven amino acid properties under positive selection. Click here for file
