# Supplementary material for: Evolution and divergence of the mammalian SAMD9/SAMD9L gene family
Source: BMC Evol Biol. 2013 Jun 12;13:121. doi: 10.1186/1471-2148-13-121 (PMC3685527; doi:10.1186/1471-2148-13-121)
Supplement: Additional file 1: Figure S1 — Mammalian SAMD9 and SAMD9L deduced protein sequences alignment. SAMD9 and SAMD9L genes coding sequences were collected for fifteen and nineteen species, respectively. Sequences were aligned with ClustalW implemented in BioEdit. The abbreviations correspond to the following species common names: Hosa - Human; Patr - Common chimpanzee; Gogo - Western gorilla; Poab - Sumatran orangutan; Nole - Northern white-cheeked gibbon; Mamu - Rhesus monkey; Bota - Cow; Susc - Pig; Eqca - Horse; Mylu - Little brown myotis; Orcu - European rabbit; Rano - Brown rat; Crgr - Chinese hamster; Capo - Domestic Guinea pig; Soar - Common shrew; Caja - Common marmoset; Loaf - African bush elephant; Calu - Domestic dog; Aime - Giant panda; Ereu - West European hedgehog; Mumu - House mouse; Modo - Grey short-tailed opossum. To access the species scientific names, the list of abbreviations should be consulted. Codons are numbered according to human SAMD9 protein. “?” represents undetermined codons; “.” represents identity with the reference sequence of human SAMD9 protein. [file 1471-2148-13-121-S1.pdf]

```
                        10        20        30        40        50        60        70        80        90       100
               ....|....|....|....|....|....|....|....|....|....|....|....|....|....|....|....|....|....|....|....|
SAMD9_Hosa     MAKQLNLPENTDDWTKEDVNQWL-ESHKIDQKHREILTEQDVNGAVLKWLKKEHLVDMGITHGPAIQIEELFKELRKTAIEDSIQTSKMGKP--SKNAPK
SAMD9_Patr     .......................-....................................................................--......
SAMD9_Gogo     .......................-..............G....................................Q...T............--......
SAMD9_Poab     .......................-..............G..............................................M......--......
SAMD9_Nole     .................E.....-..............G.....................................................--...V..
SAMD9_Mamu     ........Q...........R..-..............G....................................Q.............R..--......
SAMD9_Bota     ..A....................-.....E....A...A...S..I..Y.T.D...A....F.......Q.....LE.SSG.PF..C.S..G--..SV..
SAMD9_Susc     ..A.....K...........R..-..........D...A......N..Y.T....I.I...F.......H.....ME.ST..PS.IC.RE.G--..DV..
SAMD9_Eqca     ..A.................R..-.............IA...S..I....T.NN.IE............N.....Q..SS.G.L..N.KK.G--...V..
SAMD9_Mylu     ..A.............D...R..-D.........K.............F.T.N..IE............Q...K.Q.ESS..P...CQKK.G--..KV..
SAMD9_Orcu     ..E.PE..............R..-.....H....D..VA...S.....F...SD.IE........V...G..R..Q..SSK.P.R..EQK.G--...VSN
SAMD9_Rano     ..ET....K...........L..-...R.........MA...S.V.....N..KN..E..................Q..PK.NLTK.C.KT.G--R..I..
SAMD9_Crgr     ..EK.......N........L..-.G.........T.ML...........D.N...E............A.....Q.SSSKQPT....RKRG--...I..
SAMD9_Capo     ..E.PH....I.........R..-.............IA...S..T....N.SD......P.......VG..N..Q..SS..PV..P.AK.S--.T.T.G
SAMD9_Soar     ..A.P..SK.P.........I..-.E.N..K.Y....VA.....D...L.T.K......V......L..N.V.K.Q..SS.N....REKKTN--N..I.E
SAMD9L_Hosa    .S..VS...MIK.....H.KK.VN.DL..NEQYGQ..LSEE.T.L..QE.TEKD..E..LPW...LL.KRSYNK.NSKSP.SDNHDPGQLDN--..--.S
SAMD9L_Patr    .S..VS...MIK.....H.KK.VT.DL..NEQYGQ..LSGE.T.L..QE.TEKD.IE..LPW...LL.KRSYNK.NSKSP.SDNHDPGQLDN--..--.S
SAMD9L_Gogo    .S..VS...MIK.....H.KK.VN.DL..SEQYGQ..FSEE.T.L..QE.TEKD.IE..LPW...LL.KRSYNK.NSKSP.SDNHDPGQLDN--..--.S
SAMD9L_Poab    .S..VS...MIE.....H.KK.VT.DL..NEQYGQ..LSEE.T.L..QE.TEKD.IE..LPR...LL.KRSYNKFYSKSP.SDNHDPGQLDN--..--.S
SAMD9L_Nole    .S..VS...MIK.....H.KK.VT.DL..NEQYGQ..LSEE.T.L..QE.TEKD.IE..LPR...LL.KRSYNK.NSKSP.SDNRDPGQLDN--..--.S
SAMD9L_Caja    .S..VT...MIK.....H.KK.VT.DL...E.YGQ..LNEE.T.L..QE.TEKD.IE..LPR...LL.KRSYNK.NN.SA.SDNHDPGQLSH--..--SS
SAMD9L_Mamu    .S..VS...IIK.....H.KK.VTDDL..NEQYGQ..LSEE.T.L..QE.TEKD.IE..LPR...LL.KRAYNK.NSKSP.SDNHDPGQLDH--..--.S
SAMD9L_Loaf    .SE.VT...M.Q.....H.KK.VT.DL...E.YGQ..LNEE.T.L..QE.TEKD.T...LPR...LL.KRGYNK.NNSSP.SHN.DFEQLDH--T.--TS
SAMD9L_Eqca    .NE.A....M.K.....H.K..VTKDL..GE.YGQ..LNEE.T.L..QE.TEKD.IE..LPR....L.KRAYNR.NNSSP.SNN.D.GQLDH--T.--SS
SAMD9L_Calu    .NEEV....VV......H.K..VTKHLNV.E.YGQ..LREE.T.L..QE.TEKD.RE..LPW.S.LL.KRKYNK.NNSSS.SNN.D.GQLDH--T.--.S
SAMD9L_Aime    .SE.V....IIN.....H.K..VTKDL...E.YGQ..LTEE.T.L..QE.TEND.RE..LPR...LL.KRAYNR.NNSSS.SNN.D.GQVDH--T.--.S
SAMD9L_Ereu    .DE.V....M.Q.....H.K..VTNDLQ..E.YGQ..LCEE.T.R..QV.TEKD.IE..LPR...LL.KRK..S.NNLSSKN.N.N.GQLDH--AE--.S
SAMD9L_Orcu    .NE.VT...LVK.....H.KK.VT.DL.V.E.YGQ..LNEE.T.L...E.TEDD.KE..LPR...LL.KRACNK.LNSSP.SDN.D.GKLDN--I.--.S
SAMD9L_Mumu    .SG.VTQ.KLIK.....H.RK.VT.DLN.VE.YAQ..FKEE.T.M..QE.TE.D.RE..LPR...LL.KRMYNK.IS-SP.SHN.D.RELND--K.--LS
SAMD9L_Crgr    .NE.VTA.KLVK.....Q.KK.IT.DLN..E.YA...FNEE.T.M..QE.TEKD.RE..LPR...LL.KRAYNK.SN-.T.SDN.D..QLHN--K.--LS
SAMD9L_Rano    .DRHVTQ.KLIK.....H.RK.IT.DL...E.YAQ.VF.EE.T.M..QE.TEKD.RE..LPR...LL.KRMYNK.IS-SP.GHN.D.RQLNN--KT--LS
SAMD9L_Capo    .NE.GT...MIK....DH.KK.IT.EL...E.YGQ..FNEE.T.L..QE.TEKD.KE..LPR...LL.KRMYNK.NTSFP.SDNPN.RQVHD--T.--SS
SAMD9L_Soar    .SEHT....M.KA......K..VT.VLQ..EEYGQ..LNEK.S.LA.QEITE.D.RE..LPR...LL.KRTYNK.NNISP.SNSPN.RPLDS--TN--VS
SAMD9L_Modo    ..E.T.....P....V...C..IT.KL..NS.YT...KKEE...KS..VSD.ND......K....L..INS....N.-SS.CPK...EEQ.DKQTE--VQ
```

```
                    110       120       130       140       150       160       170       180       190       200
             ....|....|....|....|....|....|....|....|....|....|....|....|....|....|....|....|....|....|....|....|
SAMD9_Hosa   DQTVSQKERRETSKQKQKGKENPDMANPSAMS--------------TTAKGSKSLKVELI--EDKIDYTKERQPSIDLTCVSYPFDEFSNPYRYKLDFSL
SAMD9_Patr   ...............R................--------------..............--...........L..........................
SAMD9_Gogo   ................................--------------..............--......................................
SAMD9_Poab   ........H.........D..........TI.--------------.I..S........M--..Q.............N...................I.
SAMD9_Nole   .......A.....................T..--------------.I............--..E...........................H.......
SAMD9_Mamu   ........S.........D..........T..--------------.I..V.E.......--....................................I.
SAMD9_Bota   T.----QKDGG...--..N.KKS.KV.D.TV.--------------.VT.......N.FM--..E..D..KK...TEP..MA.......D......N.K.
SAMD9_Susc   K.----EKN.........D..TS.RVTD.TI.--------------.VTE..M..NN.FM--..E.ND.QKK.S.VE.M..P.......D......N...
SAMD9_Eqca   T...M.E.NG........N..KS....APT..--------------.VT.D.....N.FK--VNE..D...K...VE............D......N...
SAMD9_Mylu   T..LM.E.NE......H....KS....A.T..--------------.V---.....S.F.--G.E..D...K.................D........C.
SAMD9_Orcu   K.PLV...K.D.......N...S.P.DG..A.--------------PGE.EP..P.T..TDN..ERGD...KL.AKEPS.R.H..NK.DEQW....H.I.
SAMD9_Rano   T.----T.S....N.N.RAE..SCK.DT..V.P---EGNSQRTAASPE......E.ND.V--...AVQ-QDK.A.PGP..IA...N..........H.I.
SAMD9_Crgr   T.----T.I...P..N.R.E..T.K.DA..LR--------------PE...AE.A.N..M--...TEL-QGK.SPPGP..IA...N..........H.I.
SAMD9_Capo   K.HLM...G......R.SD..ISHK.-----------------------.E...PDS..M--.NQP.D------VKQP..LP...NT.........H.I.
SAMD9_Soar   V..L..S.NG.K..K...D..KS.TVDT.TTH--------------.V..S...VEN.F.--..D.---Q.K.L.TEQ..IP.......D.....EN.V.
SAMD9L_Hosa  KT-----.HQKNP.HTK.EE..SMSS.I-DYDP---REIRDIKQEESIL----MKENV.DEVANAKHKK.GKLKPEQ...MP....Q.HDSH..IEHYT.
SAMD9L_Patr  KT-----.HQKNP.HTK.EE..SMSS.I-DYDP---REIRDIKQEESIL----MKENV.DEVANAK.KK.GKLKPEQ...MP....Q.HDSH..IEHYT.
SAMD9L_Gogo  KT-----.HQKNP.HTK.EE..STSS.I-DYDP---REIRDIKQEESIL----MKENV.DEVANAK.KK.GKLKPEQ...MP....Q.HDSH..IEHYT.
SAMD9L_Poab  KT-----.HQKNP..TK.EE..SMSS.I-DYDP---REIRDIKEQESIL----MKENV.DEVANAK.KK.GKLKPEQ...MP....Q.HDSH..IEHYT.
SAMD9L_Nole  KT-----.HQKNP..TK.EE..SKSS.I-DYDP---REIRDVKQQESIL----MKENV.DEVANAK.KK.GKLKPEQ...MP....Q.HDSH..IEHYT.
SAMD9L_Caja  KT-----.HHKKP..TK.KEK.SMSSSI-DYDP---REVRDIKEQKSIL----MKENV.DEA.NAK.KK.DKLKPER...MP....Q.HDGQ..IEHYT.
SAMD9L_Mamu  KR-----.HQKDP..TK.EE..STSS.I-DYDP---REVRDIKERESIL----MKENV.EEVANAK.KK.GELKPEQ...MP....Q.HDSH..IEHYT.
SAMD9L_Loaf  KK-----.Q-KKA.-----..KSISSSI-DHDL---TEIGDIKERESIL----MKENATNEVAATK.KK.NKVKTEQ...MP....Q.HDSQ..TEHYI.
SAMD9L_Eqca  KE-----.HK.KPQ.TK.E..KSTSS.I-DHNL---RETRDTKEQESIL----MKENA.NEVV-TK.KQ.NKLQAEQ...MP....Q.HDSQ..IEHYI.
SAMD9L_Calu  KK-----.HPKKPQ.MK.EE.KSVLS.I-DHDL---REARDTKEQESIL----MKEDA.NEGATAE.QNEDKLGIKQ...MP....Q.H.SHC.IENSV.
SAMD9L_Aime  KK-----.HPKKPQKMK.EE.KSVLS.N-DHDL---REMRDTKEQESIL----VKEDA.NEEVTTE.QNED.TETEQ...MP....Q.H.SQ..IEHSI.
SAMD9L_Ereu  KK-----.HQRK--H---KT..T-LS.N-DHDV---REIQNAKAQELA.----TG.NAQDEVGITEEK-.KKRKIVQS..MP....Q.HASHH.TEHYI.
SAMD9L_Orcu  KK-----KQQKKP...S.EEGTSMSL.I-DHDL---RETTEIEVQESIP----LKEKA.DETVNAA.K-ENAIQTER...MP....Q.HDSQ..IEHYI.
SAMD9L_Mumu  TK-----.QQTK---TKNEE..SVSS.S-DHGL---RETGQNEEQEPSL----TKENM.GDVV-TK.MEDNKPKPEQMS.TP....S.CDVKQ.IEHSI.
SAMD9L_Crgr  IK-----.HPKK---TNNEE.KLISS.S-GHDL---REMGLNTEQEPSL----LKEKA.SDVL-TK.MEGNTAKPEQMS.MP....S.HDDR..IERYI.
SAMD9L_Rano  IN-----.QPKK---SNSEE..SISS.S-DFGL---RETGQNEEQEPSI----MKVNT.GDV.-TK.MKDNMPK.EQMS.MPH..NFAHDAK..IEHSI.
SAMD9L_Capo  KK-----.HQKKP----NLEKELMPSSI-DQDL---SESRNIKDQDSIP----MEENAANEVSNT..KK.NKLKTEN...PP....N.HDGQ..IEHYI.
SAMD9L_Soar  KK-----KSKKN.Q--NTKE.KLVSS.S-DHDL---KESVNTKEQESVP----IE.DS.DYQGIPE.Q-.GKLKKEQS...P....Q.HDSR..VEHQI.
SAMD9L_Modo  --W.T.EKN.KR------RR---HHTD.-KNDTTTQDEEVYESPKTNPS----KGTESNAEET------.DNL.FRGQ..QT....K.HKSF..REQTII
```

```
                       210       220       230       240       250       260       270       280       290       300
              ....|....|....|....|....|....|....|....|....|....|....|....|....|....|....|....|....|....|....|....|
SAMD9_Hosa    Q-PETGPGNLIDPIHEFKAFTNTATATEEDVKMKFSNEVFRFASACMNSRTNGTIHFGVKDKPHGKIVGIKVTNDTKEALINHFNLMINKYFEDHQVQQA
SAMD9_Patr    .-..................................................................................................
SAMD9_Gogo    .-.......................................................................S..........................
SAMD9_Poab    .-.........................................................I.............S..........................
SAMD9_Nole    .-.......................................................................S..........................
SAMD9_Mamu    .-.....................E.............................................V...S..........................
SAMD9_Bota    .-.....L...............E.G....A..................................T...VEF.TI......D......HQ........K.
SAMD9_Susc    .-.....L...............E...K.........................................VE..TV....F.D...M..HQ........K.
SAMD9_Eqca    .-.....L...............ER.R...I......................................V..STV...T..D......QQ........K.
SAMD9_Mylu    .-.....L..........E....KN............................................VN..SV......D......HQ........K.
SAMD9_Orcu    .-..--------------------------------------?.............L......-.Q...V.LASI..N...D..DE..P....E......
SAMD9_Rano    .-.....L.....V.....L...E......I............................Q...Q.R...MEL.TV..D...D......VQ...V......
SAMD9_Crgr    .-.....L.....V.........EK.S...I.......I........................Q.R...VDLSTV..DT.........DQ...A......
SAMD9_Capo    .-.....Q...........L.D......K.I.......T..............................M...SV......E...S..PQ...A......
SAMD9_Soar    .-.....L............K.IEKG.K..I.I.......K..................E.......I.VRFIAI...V..D...QI.HQ...EG...K.
SAMD9L_Hosa   .-....AL...........L...E....V.I..................................E...V.I.SKA--.F.D...V..K....ESEINE.
SAMD9L_Patr   .-....AL...........L...E....V.I..................................E...V.I.SKA--.F.D...V..K....ESEINE.
SAMD9L_Gogo   .-....AL...........L...E....V.I..................................E...V.I.SKA--.F.D...V..K....ESEINE.
SAMD9L_Poab   .-....AL...........L...E....A.IN.................................E...V.I..KA--SF.D...V..R....ESEINE.
SAMD9L_Nole   .-....AL...........L...E....A.I..............................N...E...V.I.SKA--.F.D...V..K....ESEINE.
SAMD9L_Caja   .-....SL...........L...ER.....I........L.........................E...VNI.SKD--VF.D......K....ESDINE.
SAMD9L_Mamu   .-....AL...........L...E....A.I..................................E...V.I.SKD--.F.D......K....ESEIN..
SAMD9L_Loaf   .-.....L...........L...D....K.IH.................................E...V..ASKD--DF........KQ....SDIKE.
SAMD9L_Eqca   .-.....L...........L...E....K.I.......A.....................N....E...V...SKD--SF.D...V..KQ...ESEIKV.
SAMD9L_Calu   .-.....L...........L...EA...K.I.............................NR...Q...V..A.KD--.F.D...I..RQ...ESEINEV
SAMD9L_Aime   .-.....L...........L...EA...K.I.............................N....Q...V...SKD--.F.D...V..RQ...GNEINE.
SAMD9L_Ereu   .-.....L...........L...E....K.I............................L.....E...V..RSKD--.F.D......KQ...ESEIKE.
SAMD9L_Orcu   .-.....L...........L....A.....I..................................E...V.ISSKD--V......V..K.....SDINE.
SAMD9L_Mumu   RVA....L...............KK.....I.......T....A.....................E...VQ..SKD--IFV....T..T.....SEISE.
SAMD9L_Crgr   .VA....L.....V.....L...E......I.......I..........H...............E...V.IPSKD--VFVD...K..K.....SDIKE.
SAMD9L_Rano   RVA....L.....V.........E...Q.QM.......T....A....T................E...VQ..SKD--IF.....V..T.....SDIHE.
SAMD9L_Capo   .-.....L...........L...EN.....L.......T..........................E...V...SKD--.F.D......K....ESEISE.
SAMD9L_Soar   .-.....R...........L...ESVE.K.I............................L...Q.E...V...RKD--SF.D...K..KH....SEIKE.
SAMD9L_Modo   .-....SF...........L...ER.....IL................C............S...E...L.L.EED..KFVD..YKK.EV...KDD.S..
```

```
                      310       320       330       340       350       360       370       380       390       400
             ....|....|....|....|....|....|....|....|....|....|....|....|....|....|....|....|....|....|....|....|
SAMD9_Hosa   KKCIREPRFVEVLLPNSTLSDRFVIEVDIIPQFSECQYDYFQIKMQNY--NNKIWEQSKKFSLFVRDGTSSKDITKN----KVDFRAFKADFKTLAESRK
SAMD9_Patr   ........................................................--...T...............R.....----...................
SAMD9_Gogo   ........................................................--...T.....N.................----...................
SAMD9_Poab   ........................................................--...T............E.......M..----.....T..T..........
SAMD9_Nole   ..................A.............................--...T..............P.....M..----...................
SAMD9_Mamu   ............................................I...--...T........V...........M..----.A...T.............
SAMD9_Bota   .N..........................VV.KY...EV.........C--S..T.K..PN..V.....A.....M.S----NM..K...L.L.R......
SAMD9_Susc   .N...............IP.........VV.KY...EQE.........--S..T.K..P...V.M...A....VM..----N.E.KT..L.L.K...A..
SAMD9_Eqca   ..................P.........V..KY...ED...R..I..C--D.NT.K..S...I.....A.....M..----NA..KE..L.L.....A..
SAMD9_Mylu   ..................P.........V..KY...EH.........C--...V...R.H..V.....A.....M..----....KI.QLGLEA...H..
SAMD9_Orcu   .S...V.........D..V.........V..HY.V.GH......K.I.DN...K....S...VL....ARTVN.V.T----.T..KM..LNL........
SAMD9_Rano   ..................P.NK..........Y...KE.F.....HWH--KDET.Q.IP.Y.V.....PK....IG.----.A..K...L.L.A..D...
SAMD9_Crgr   ..................P.NK......VL..H...KE........HL--KSQT.Q..S.H.V.....PQ....IG.----.T..K...LGL.EV.D...
SAMD9_Capo   ..................V.....V...VV..Y...KH...R.T....--.HET.QP.S.Y.V.I...PNT.N.I..----....E..QL.L.SV.T...
SAMD9_Soar   .N............T.GIP...Y.........KY...EH.........F--...T.NK.SR..V.....A.C..LM.T----PA..K.Y.S.L........
SAMD9L_Hosa  ..............Q.N.P.........T..KH.I.NDK..Y.Q..IC--KD...K.NQNL.....E.A..R..LA.SKQRD...K..LQNL.S.VA...
SAMD9L_Patr  ..............Q.N.P.........T..KH.I.NDK..Y.Q..IC--KD...K.NQNL.....E.A..R..LA.SKQRD...K..LQNL.S.VA...
SAMD9L_Gogo  ..............Q.N.P.........T..KH.I.NDK..Y.Q..IC--KD...K.NQNL...I.E.A..R..LA.SKQRD...K..LQNL.S.VA...
SAMD9L_Poab  ..............Q.N.P...........KH.I.KDK..Y.Q..IC--KD.T.K.NQNL.....E.A..R..LA.SKQRD...K..LQNL.S.VA...
SAMD9L_Nole  ..............Q.N.P..........N.KH.I.KDK..Y.Q..IC--KD...K.NQNT.....E.A..R..LA.SKQRD...K..LQNL.S.VA...
SAMD9L_Caja  ..............Q.N.P.......I....KH.V.KDM..C.R..S.--TD.K.K.N.NS.....E.A..R..LADPNKRD...K..LQNLES.VA...
SAMD9L_Mamu  ..............Q.NMP............KY.I.KDK..Y.Q..IF--KD...K.NQNS.....E.A..R..LA.SKQRD...K..LQNL.S.VA...
SAMD9L_Loaf  E....Q.......QQ.N.P..K......V..RH.V.KDK..Y.N..IC--KDGS.R.D.DY.....Y.A.....LA.VRQRDA..K..TLN.ESVVA...
SAMD9L_Eqca  ..............Q.N.P.........V..KH.I.KEK..Y.....C--K.EK.K.NEDH.......A.....LA.GKQRDT..K..TQNL.S.VT...
SAMD9L_Calu  ..............Q.N...........V..KH.V.EKK..L.R...C--KSET.KPNQDL.......P.....LA.VKQRELNLK..LQNLTSVVA...
SAMD9L_Aime  ..............Q.N.P.........V..KY.V.GEK..L.R..SC--K.ET.KPNQDL.......P.....LA.VKQRDIAYKE.SQN..SV.T...
SAMD9L_Ereu  ..............Q.Y.P.........V..KH.I.KEK..YTN..S.--K.DT.K.NQES.......A..V..LA.GKQRDKN.KT.LQNL.S...D..
SAMD9L_Orcu  ..............Q..MP.........V..KH.V.KEK..Y....SC--KDNV.K.NQEY...I.E.A..R..LA.AKQRDP..K..TQNL.S.VA...
SAMD9L_Mumu  RA............Q.N.Q.N.......V..RH.I..EK..Y.M..SS--TG.T.K...DT.....E.A...N.LG.PNQRDRE.KK.LE.L.MWTA...
SAMD9L_Crgr  RE............Q.N.P.........V..KH.I.KEK..Y.I..S.--TD.T.K...ENC....E.....N.LA.AKQRDRE..K.LENL.AWTA...
SAMD9L_Rano  RA............L.N.Q.N.......V..KH.I..EK..YVML.TC--TGTT.K...DT.....E.A..R..LG.PKQRDRE.KK.LENL.MSIA...
SAMD9L_Capo  Q....Q...ID...Q.N...............KY.V.KEK..Y..L.SL--TD.T.K.NQNL.......A.....LA.VKRREIE.K..YENL.SC.AA..
SAMD9L_Soar  R.............Q.N.S.........V..KH.I..EK..YT...EL--KDG..K.TENT.....E.A.T...LA.ERTRDAG.K..LQ.L.S.ITM..
SAMD9L_Modo  .Q...P........K.NIV.......I..V.KH.I.ENK...V...IF--KENK.GKNAEL..Y........N.YA.PKQKDGNIK..L.ELEKCVKL..
```

```
                    410       420       430       440       450       460       470       480       490       500
             ....|....|....|....|....|....|....|....|....|....|....|....|....|....|....|....|....|....|....|....|
SAMD9_Hosa   AAEEKFRAKTNKKEREGPKLVKLLTGNQDLLDNSYYEQYILVTNKCHPDQTKHLDFLKEIKWFAVLEFDPESNINGVVKAYKESRVANLHFPSVYVEQKT
SAMD9_Patr   ....................................................................................................
SAMD9_Gogo   ....................................................................................................
SAMD9_Poab   .................................................................................................E..
SAMD9_Nole   ..........................................................................K......................E..
SAMD9_Mamu   ..............................................................................QD..A..........I...G..
SAMD9_Bota   E....C.V......S.....................DW....I.....T.I.....................VSK........T..V......L...G..
SAMD9_Susc   E....C.L......S............K........DW....I.....T.I.....................VSE...........V......L...G..
SAMD9_Eqca   E....Y.L..S...N.....................DW..........N.I.....................VS...........IT......L.M.G..
SAMD9_Mylu   E....C.I...N.NS.....IE.....K.........W..........N.I...............D.....VSK...N......IV...L..L...G..
SAMD9_Orcu   E..DNCT....NRH-..FR..E..............DW..........N.IQY...................ES....RSF....L.......Q.S.G..
SAMD9_Rano   E..K.SKV.SDGSNSQ.Q..ID.....K......H..W..................................ES......F.G..........LFI.E.P
SAMD9_Crgr   E..T..KE.PEGNKS..Q..ID...............W...I......N.NS..E.................ES......F.Q..........QF..E..
SAMD9_Capo   E....CKL....NNS..S...T..............DW............II............I.......KTK..L..F....A....S..QF..E..
SAMD9_Soar   E...N..L..S...S......RM.............SL.........QN.VN.........L..........ASK..................Q...E..
SAMD9L_Hosa  E...EYGM.AM...S..L......I..R.S......DW..........N.I.....................M...................NQ.EDKT.
SAMD9L_Patr  E...EYGM.AM...S..L......I..R.S......DW..........N.I........M............M...................NQ.EDKT.
SAMD9L_Gogo  E...EYGM.AM...S..L......I..R.S......DW..........N.I.....................M...................NQ.EDKT.
SAMD9L_Poab  E...EYGM.AI...S..L......I..R.S......DW..........N.I.....................M...................NQ.EDKT.
SAMD9L_Nole  E...EYGM.AM...S..L......I..R.S......DW.V........N.I.....................M.........Q.........NQ.EDKT.
SAMD9L_Caja  E...EY.M.AK...S..L.........R.S......DW....I.....N.I.....................QS.......R..........NQ.EDKTM
SAMD9L_Mamu  E...EYGM..M...S..L......I..R.S......DW..........N.I.....................V...................NQ.EDKT.
SAMD9L_Loaf  E...EYGV.A....S..L..A...I.SR.S..D...DW.......S..N.......................VSK.................DQ.EKKAA
SAMD9L_Eqca  E...EYEV.A....S..Q......I..R.S......NW....I.....N...N...................QSK..A..F.K..........Q.E.KS.
SAMD9L_Calu  E...EYEM.ADR..S..Q......I..R.S......NW..........N...N......M.L..........VSK.......K..I......NQFE.KTN
SAMD9L_Aime  V...EYEV.A....S..Q......I..R.S......SW..........N...N......M............VSK.......K.........NQFE.KSN
SAMD9L_Ereu  D..KEHEM.A....C..Q..I...I....S......NW........N.S.R--...................ESK..A.T.R........-.NQ.A.KT.
SAMD9L_Orcu  E...EYGR.AT...S..L......I..R.S......SW..........N.......................G....................Q.EDKTS
SAMD9L_Mumu  ....EL.M-VT...S..L..S....RH.GS..E...DW......T.A.T.LE..E.I..M.L....D...Y.H.K......R...I....L..H.E.K-.
SAMD9L_Crgr  E...QQ.M-VT...S..L..A....RH.GS......DW......N.A.N.IE.........L....D...Y.Q.K......R...I....L..Q.E.K-S
SAMD9L_Rano  ..-.ECMV-VS..DS..L..S....RH.GS..K...DW......S.V.T.ME....I....L....D...Y.H.K......R........L..H.E.K-.
SAMD9L_Capo  D...EHQM..T.R.S..L......I..R.S......NS..........N.MQ....................R....A...QK.....M...NQ.E.K..
SAMD9L_Soar  E....DEV.V..Q.S..Q..I...I..R.S..S...NW..........N...Y.......S.....D.....VSK....VFQ...........Q...G.S
SAMD9L_Modo  E.-.DHQP.EI.T.N..T...H.......-......D.....V..S..S.IE........E...........LNT..A...R.T.A......NQ.QDN..
```

```
                    510       520       530       540       550       560       570       580       590       600
             ....|....|....|....|....|....|....|....|....|....|....|....|....|....|....|....|....|....|....|....|
SAMD9_Hosa   TPNETISTLNLYHQPSWIFCNGRLDLDSEKYKPFDPSSWQRERASDVRKLISFLTHEDIMPRGKFLVVFLLLSSVDDPRDPLIETFCAFYQDLKGMENIL
SAMD9_Patr   ....................................................................................................
SAMD9_Gogo   ....................................................................................................
SAMD9_Poab   ....M.....................N............................R............................................
SAMD9_Nole   .S..M............................L..................................................................
SAMD9_Mamu   ....K..................S.......................................................................I....
SAMD9_Bota   .-T.K.TS....Q.L..............E...L.A......K..E.........Q............................................
SAMD9_Susc   .-..K.IS....Q.L..................L........K..E......I..............................................M
SAMD9_Eqca   .S..K.TS....Q..........I.........L........K..E......................................................
SAMD9_Mylu   ....K..S....Q....................L......K.K..E............V....................................I....
SAMD9_Orcu   .T..M..N....Q..........S..GI.....L..I........E............V..K...........P..........................
SAMD9_Rano   .VS.K..S....Q.............E.D..Q.L..I........E..R......RD..................E.................N......
SAMD9_Crgr   .L..K..S....Q..........S..E.D....L..I........E..R....F.RD....K.R.........P.............G............
SAMD9_Capo   .TE.K..S....QK.........S.......E.V..V...K....E.........R.....................K........T.....S......
SAMD9_Soar   .TD.K..S...HQ.....-.........D....L..????????????????????????...--...-....P....-..-Y............L....
SAMD9L_Hosa  NMW.K.......Q..........S..K..T...LE.HL.......E.....L...D.N..T..............ES.G.......W....A......M.
SAMD9L_Patr  NMR.K.......Q..........S..K..T...LE.HL.......E.....L...D.N..T..............ES.G.......W....A......M.
SAMD9L_Gogo  NMR.K.......Q..........S..K..T...LE.HL.H.....E.....L...D.N..T..............ES.G.......W....A......M.
SAMD9L_Poab  NMR.K.......Q..........S..K..TH..LE.HL.......E.....L...D.N..T..............ES.G.......W....A......M.
SAMD9L_Nole  NMR.K.......Q..........S..K..T...LE.YL.......E.....L...D.N..T..............ES.G.......W....A......M.
SAMD9L_Caja  NMR.K..A....Q..........S..K..T...LEAHL.......E.....L...D.N..T.R.........FT.ES.V.......W....A......M.
SAMD9L_Mamu  NMR.K..A....Q..........S..K..T...LE.HL.......E.....L...D.N..T..............ES.G.......W....A......M.
SAMD9L_Loaf  .IR.M.F.....E..........S..NN.T...L..HL.......E.....L...D.N..TK.........F...ES.G............A........
SAMD9L_Eqca  PMR.K..S....Q..........T..KD.RH..LE.HL.......EI....Q...D.NV.T..R.........A.ES.G.....S......A......M.
SAMD9L_Calu  NIR.K..S....Q.T........S..KN.S...LE.HL.H.....E.....L...D.N..T............P.ES.G............A........
SAMD9L_Aime  NIR.K..S....E..........S..KN.S...LE.HL.H.....E.....L...D.N..T..............ES.G............A........
SAMD9L_Ereu  .MR.K..S?S.W----------------------E.RQ..E....GRA..NY...D.N..T............P.ES.G.....P..T.FKV.N..Q..M
SAMD9L_Orcu  SLR.K.......Q....V.....S..T..T....E.HL.......E.....F...D.N..A..............ES.G............A......M.
SAMD9L_Mumu  .IA.K....K..E..........V..---SCQ.LE.HL...D...G..R......D.N.IVK..V........PIENQK............VFN..D.M.
SAMD9L_Crgr  .IE.K....K.FE..............KNDSCQ.LE.HL...D...G..R......D.N..V...V........P.NNQK............VFN..D.M.
SAMD9L_Rano  .IE.K....K..E..........V..---SCQ.LE.HL...D...G..........DGN.IA...V........P.ENQK...L........VFN..D.M.
SAMD9L_Capo  .MRQK..S....E.T........S..QN.T...LEAHL...D..CE.........D.NL.AK.............ESLG..FT........A.R....M.
SAMD9L_Soar  .Q...VCS....Q.T........S..KGAV...LE.HL.NKN...E.T...L...D.S..A............P.ES.G........V..KA.N......
SAMD9L_Modo  SIS.K.ESF..FK.T........A.FNT.E.Q.LEL...IKK..TK.........C..V.QP.R.........P.E..K..F....ST...E.G...D..
```

```
                     610       620       630       640       650       660       670       680       690       700
              ....|....|....|....|....|....|....|....|....|....|....|....|....|....|....|....|....|....|....|....|
SAMD9_Hosa    CICVHPHIFQGWKDLLEARLIKHQDEISSQCISALSLEEINGTILKLKSVTQSSKRLLPSIGLSTVLL-KKEEDIMTALEIICENECEGTLLEKDKNKFL
SAMD9_Patr    ....................................................................-...............................
SAMD9_Gogo    ....................................................................-...............................
SAMD9_Poab    ....................................................................-...............................
SAMD9_Nole    ....Q...............................................................-...............................
SAMD9_Mamu    ....Q...............................................................-........S......................
SAMD9_Bota    ....DSR.C...........TTQ...L.N..V.S......................F.....S.....-............L......D.I.....K.L.
SAMD9_Susc    ...A.SR.C...........A.QE..L.............................F...V.S.....-.R..........L.............EK...
SAMD9_Eqca    .....S..C.R.........T.Q...L.N........................L..F.....S.....-........S...L..............K...
SAMD9_Mylu    .V...S..C.R.........T.-...L......S.................K..Q.F.....S.....-EM..........L.....K.....N......
SAMD9_Orcu    ......Q.C...........T.E...L.N......N.Q..................F.....S.....-..Q..T......L......D....KD.K...
SAMD9_Rano    ........Y.V......V..SSQ...L...S.FS.N.A...........L......F...V.S.....-.R..E.......L.............RE.LF
SAMD9_Crgr    ..S.FSS.Y.V.........AGQ...L.NRS....N.A...........L......F.....ST....-....-.......L...........N..K.LF
SAMD9_Capo    ....Q.N.C...........TNE...L.DK.....N..Q......N...I....E......CS.....-.....-......L.V........H...T.LQ
SAMD9_Soar    V.-LNSQLC.I.........KIQ...LAN......N.................V.K......S.....-............L......D.D.........
SAMD9L_Hosa   ..S.NS..Y.R.....QT.MKM-E..LTNHS..T.NI.LV.S.........R..R.F..AR.S.S.I.E..K..VL.....L.....TE.DI....S...
SAMD9L_Patr   ..S.NS..Y.R.....QT.MKM-E..LTNHS..T.NI.LV.S.........R..R.F..AR.S.S.I.E..K..VL.....L.....TE.DI....S...
SAMD9L_Gogo   ..S.NS..Y.R.....QT.MKM-E..LTNHS..T.NI.LV.S.........R..R.F..AR.S.S.I.E..K..VL.....L.....TE.DI....S...
SAMD9L_Poab   ..S.NS..Y.R.....QT.MKM-E..LTNHS..T.NI.LV.S............R.F..AH.S.S.I.E..K..VL.....L.....KD.DI...ES...
SAMD9L_Nole   ..S.NS..Y.R.....QT.MKM-E..LTNHS..T.NI.LV.S...........PR.F..AH.S.S.I.E..K..VL.....L.....RD.DI...ES...
SAMD9L_Caja   ..S.SS..Y.R.....QT.MNI-E..LTNHS..T.NI.LV.SS...........R.F..AH.S.S.I.K..K..AL.....L.....RD.DI...ES...
SAMD9L_Mamu   ..S.NS..Y.R.....QT.MKM-E..LTNHS..T.NI.LV.S............R.F..AR.S.S.I.E..K..VL.....L.....RD.DI...ES...
SAMD9L_Loaf   ....KSQ.Y.R.....RT..TI-A..LTNHS..T.N..Q..S........I...R.F...R.S.S.I.E.....LF.....L.....KD.DI....S...
SAMD9L_Eqca   ....NSQ.Y.R.....QT..TV-A..LANHS..T.N..L..S......P..E..R.F...S...S.I.E......L.....L.....RD.DI...EF..Q
SAMD9L_Calu   ....NS..Y.R.....QT..TI-A..LTNHS..T.N..L..S......P....LR.F...H.F.SII.E......L.....L.....KD.DI....S..Q
SAMD9L_Aime   ....NSQ.Y.R.....QT..TA-A..LTNHS..T.N..L..S......P.....R.F...H.FAS.I.E.....TL.....L.....KD.DI...ES..Q
SAMD9L_Ereu   ....T.Q.Y.R.....QT.FVM-S..LTNHS..T....LL.S..............F...R.S.S.I.E.....FL.....L.....GD.DI...EC..E
SAMD9L_Orcu   ....NSQ.Y.R.....KT..TV-AH.L.NHS..T.N..LV.S.........R..R.F...R.S.S.I.E..K..TL.....L.....RD.DI...ES...
SAMD9L_Mumu   ....NSA.Y.Q.S...QV..EI-K.DLAKHS..T.NI.LV.N........I...R.F...C.S.S.I.E.MD....S....L.....KD.DI...ESQ..
SAMD9L_Crgr   ....N...Y.R.S...QV..EI-KG.LAEHS..T.NIQLV.N........I...R.F...C.S.S.I.E.I...TL.....L.....RD.DI..NESE..
SAMD9L_Rano   ....N.S.Y.Q.S...QV..EI-K.DLAKHS..T.NI.LV.S........I...R.F...C.S.S.I.E.MD....S....L.....RD.DI...ESQ..
SAMD9L_Capo   ....NSK.Y.R.....QT..TI-G..L.KHSV.T.NI.L..S.......L....RKF...Y.S.S.I.E.....LL...Q.LS....KE.DI....S...
SAMD9L_Soar   ....NLD.YKQ.....K....E-ES.LEDHT..T.N..FL.S.........R....F...K.S.S.T.E.....TF.T...L.....KD.DI..NAS..Q
SAMD9L_Modo   S.S.DQQ.GSQ..E...GKFSS-I.-LCNR..TT....QL........PQ.E.PI.F...N.S.-II.Q..D..S.S....L.....KD.AI...E-..T
```

```
                    710       720       730       740       750       760       770       780       790       800
             ....|....|....|....|....|....|....|....|....|....|....|....|....|....|....|....|....|....|....|....|
SAMD9_Hosa   EFKASKEEDFYRGGKVSWWNFYFSSESYSSPFVKRDKYERLEAMIQNCADSSKPTSTKIIHLYHHPGCGGTTLAMHILWELRKKFRCAVLKNKTVDFSEI
SAMD9_Patr   ....................................................................................................
SAMD9_Gogo   ..............................................................................................A.....
SAMD9_Poab   ....................................................................................................
SAMD9_Nole   ..............................................................................................A.....
SAMD9_Mamu   .......................................................C.......................D....................
SAMD9_Bota   ....L...........T.........N............K..E...RG.....S.CV...........................................
SAMD9_Susc   ....L.....................N........ER..K..T...E..A.....CV...................V................M...C..
SAMD9_Eqca   D..T.....................K...L.........I..E....W.......CV.....................................M......
SAMD9_Mylu   ....L.....................N............K..R..H.......S.CS...........................................
SAMD9_Orcu   .......KA.................NH..A................W......ICA...........................................
SAMD9_Rano   ...T...K..................N...S.......VK..KK..EW....Q.VCA.....................................E.....
SAMD9_Crgr   ...TA..Q..................N...S........K..KK..EW....Q.VCA......................D..............E.....
SAMD9_Capo   ...T...................-..Y...S........K.KE....Y..P..LMCV...........................................
SAMD9_Soar   A..TL....................KN...A........K.KD....W-....S.CV...Q..................N....................
SAMD9L_Hosa  ...K....H.................N...D.....S..K.KDL.HCW.E.P..IFA...N...............V..D.K.N..........T..A..
SAMD9L_Patr  ...K....H.................N...D.....S..K.KDL.HCW.E.P..IFA...N...............V..D.K.N..........T..A..
SAMD9L_Gogo  ...K....H.................N...D.....S..K.KDLVHCW.E.P..IFA...N...............V..D.K.N..........T..A..
SAMD9L_Poab  ...K....H.......C.........N...D.....S..K.KDL.RCW.E.P..IFA...N...............V..D.K.N..........T..A..
SAMD9L_Nole  ...K....H.................N...D.....S..K.KDL.HCW.E.P..IFA...N...............V..D.K.N..........T..A..
SAMD9L_Caja  ..RK....H.................N.P.D.....S..K.KDL..CL.E.P..IFA...N...............V..D.KE...........T..V..
SAMD9L_Mamu  ...K....H.................N...D.....S..K.KDL..CW.E.P..IFA...N...............V..D.K.N..........TG.A..
SAMD9L_Loaf  ..RK....H.....R...........N...A.....S..H.KDL..CW.E.P..VFA...N...............V..N.K.N.........AT..T..
SAMD9L_Eqca  ...K...KY.................N...A.....S..K.KDL.LCR.Q.P..PFA..VN..............NV..D.K.N.........AT..A..
SAMD9L_Calu  ...KL...H.................N...A.....S..K.KVL.KCW.E.P..EFA...N..............NV..D.K.N..........T..E..
SAMD9L_Aime  ...T....H.................N...A.....S..K.KDL..CW...P..VFA...N..............NV..D.K.S..........T..G..
SAMD9L_Ereu  ..RK....H.................N...-.I...R..E.RSL.EDW.E.P..VFA...N..............NV..DF............AT..A..
SAMD9L_Orcu  ...K....H.................N...A.....S.KE.KNL..CW.E.PR.VFA...N...............V..D.K.N.........AT..A..
SAMD9L_Mumu  ...K.R..H.....R...........N...A.....SF.E.TTL..Q....P..VFV.V.N...............V..D.KQ..........AT..V..
SAMD9L_Crgr  K..KLR..H.....RA..........N...A.....G..E.TTL..Q....PE.VFA.V.N...............V..D.K...........AT..L..
SAMD9L_Rano  ...KLR..H.....R...........N...A.....NF.E.TTL..Q....P..VFA.V.N...............V..D.KQ..........AT..I..
SAMD9L_Capo  ..RK.R..H.................N...A.....M.KN.KDL.ER..ECP..EFA...N..................D.KQ.Y........AI..E.V
SAMD9L_Soar  ..IK.N..Q....S............NHT.A.....R..N.KKL.EDW.E.P..LFA...N..............NV...............I.A..A..
SAMD9L_Modo  ..QMN..KH..L...........Y..NHT.D.IR..S..I..K...SL.N.PRESGAEV.N....................K.N.......S..FASED.
```

```
                    810       820       830       840       850       860       870       880       890       900
            ....|....|....|....|....|....|....|....|....|....|....|....|....|....|....|....|....|....|....|....|
SAMD9_Hosa  GEQVTSLITYGAMNRQEYVPVLLLVDDFEEQDNVYLLQYSIQTAIAKKYIRYEKPLVIILNCMRSQNPEKSAR-IPDSIAVIQQLSPKEQRAFELKLKEI
SAMD9_Patr  ....................................................................K...K-......L...................
SAMD9_Gogo  .........................................................................-..........................
SAMD9_Poab  .................................................V.......................-..........................
SAMD9_Nole  ..................I...................S.............................K....-..........................
SAMD9_Mamu  .....N..........D........................................................-..........................
SAMD9_Bota  .....N.....TT.N...L.I.................A..H..V.NR..............R.........K-.S....L...................
SAMD9_Susc  .....N.....TASS...L................F..A....T..N...............R.........K-N.N...L..N..S...........K.
SAMD9_Eqca  ....AT......T.Q...L...........E.......S.....V.N.......................C.K-NL.G..L..N...........F.....
SAMD9_Mylu  .K...N.....TA.H...L............GDI....A......TS...............V.......C.K-MS....L.....S.............
SAMD9_Orcu  .....N.....TT.H...L...............F...S.....V.E.H.......................K-....V.LLHH.............EK.
SAMD9_Rano  .....N......T.H...L...................T.....V...H.............T.........K-N...V.LV....D..........T..
SAMD9_Crgr  .....N......TS....L...................A.....V.N.H.............T.........K-....V.LVH...D.............
SAMD9_Capo  ....V.......T.H...L...................AA....V.Q...............A.........K-......LR.K..R..KE.........
SAMD9_Soar  .K.I.D.....TTSH...L............E......S..H..VSS.H.................D.R.C.KNF.N...L......S............
SAMD9L_Hosa A...IN.V..R.KSH.D.I............E...F..NA.HSVL.E.DL....T..........R..DE..K-LA....LNY...S......GA.....
SAMD9L_Patr A...IN.V..R.KSH.D.I............E...F..NA.HSVL.E.DL....T..........R..DE..K-LA....LNY...S......GA.....
SAMD9L_Gogo A...IN.V..R.KSH.D.I............E...F..NA.HSVL.E.DL....T..........R..DE..K-LA....LNY...S......GA.....
SAMD9L_Poab A...IN.V..R.KSH.D.I............E...F..NA.HSLL.E.DL....T..........R..DE..K-LA....LNY...S......GA.....
SAMD9L_Nole A...IN.V..K.KSH.D.I............E...F..NA.H.VL.E.DL....T..........R..DE..K-LA....LNY...S......GA.....
SAMD9L_Caja .......V..K.KSH.D.I............E...I..NV.HSVL.E.DL...NT.............DE..K-SA....LNY...S......GA.....
SAMD9L_Mamu ....IN.V..K.KSH.D.I............E...F..NA.HSVL.E.DL....T..........R..DE..K-LA....LNY...S......GA.....
SAMD9L_Loaf .D...N....K.TSH...I...........LE.ICI..DA.NSSL...GL....T.............DR..K-SA..V.LKY...........A..E..
SAMD9L_Eqca ..........K.TSH.D.I...........PE..CV..NA...IF.E.DL....T.............DE..K-SAY...LMHR.........GD.....
SAMD9L_Calu V....K....K.TSHED.F..........D.E..CV..NA.DSILGE.GL....T.............DET.K-LA..V.LTY...S.......A..E..
SAMD9L_Aime V....K....K.TSPED.F..........D.E...V..NA.DSIL.E.GL....T.............DET.K-LA..V.LTY...........A..E..
SAMD9L_Ereu .....N..A.K.SSY.D.I............E..FV..NA.HSIL.E.DL....T.............DE..K-LD...VLKY......K...ST.....
SAMD9L_Orcu .....N....K.TCH.D.I............E...I..NA.NSIL.E.DL....T.............DE..K-LI....LKY..........GA..E..
SAMD9L_Mumu ....SK.MS.K.TSHEDFI............E.A.I..NA.NAF..E.GL....T.............DE..K-LAN..SLKY...........A..Q..
SAMD9L_Crgr ....SK..S.K.TSH.D.I............E.T.I..NT.NSF..E.GL....T.............DA..K-LA...SLKY...........A..Q..
SAMD9L_Rano ....SK..S.K.SSH.D.I............Q.T.I..NA.NSF..E.GV....T.............DE..K-LA...SLKY......K....A..Q..
SAMD9L_Capo ...IIH.V..K.TSH.D.I............E.I.I..NA.HSVL..RDLQ...T..V..........DQ..K-LA...SLKYA..A.......A..E..
SAMD9L_Soar .....K....K.SSH.D.I...........LE...N..DA.HSSL.E.DV....T..............E..K-LA....LTN...L..R...GA.....
SAMD9L_Modo .A..RT.V...SEDQHS.M..........D.EA.SD.RNH.IS.LEEA..Q..T.................D.K-N...V...NK..S......VK.F...
```

```
                         910       920       930       940       950       960       970       980       990      1000
                ....|....|....|....|....|....|....|....|....|....|....|....|....|....|....|....|....|....|....|....|
SAMD9_Hosa      KEQHKNFEDFYSFMIMKTNFNKEYIENVVRNILKGQNIFTKEAKLFSFLALLNSYVPDTTISLSQCEKFLGIGNKKAFWGTEKFEDKMGTYSTILIKTEV
SAMD9_Patr      ....................................................................................................
SAMD9_Gogo      ....................................................................................................
SAMD9_Poab      .................................................T..................................................
SAMD9_Nole      ........................M...........................................................................
SAMD9_Mamu      ...................................................................R................................
SAMD9_Bota      ED.....Q..............K...D.......E...S.................................TS..........................
SAMD9_Susc      E......K............D.K...............C.................................T..........L................
SAMD9_Eqca      E...E..K.............EK...............S............................N....T.......V...............R...
SAMD9_Mylu      E...................D.K...............S.................................TT...Y.....L................
SAMD9_Orcu      .KEYD...............................DPS............................N....A..........L.E..........R...
SAMD9_Rano      ......V..............................VT.......A......AF............L....S....Y...................S..
SAMD9_Crgr      .KH.E...............DRK..............VA.......A......T.............YY...S........................S..
SAMD9_Capo      ..H......................KK..........KS.................SG..........Y...A..........L............Q.K.
SAMD9_Soar      EK....V.............D.M......K........C..................E.........Q....T......EL..L..............D.
SAMD9L_Hosa     EK....C.N........S..DET.............DVDS...Q.I......S...T.S...V....I....IYTSTP.EP.SL.........L......
SAMD9L_Patr     EK....C.N........S..DET.............DVDS...Q.I......S...T.S...V....I....IYTSTP.EP.SL.........L......
SAMD9L_Gogo     EK....C.N........S..DET.............DVDS...Q.I......S...T.S...V....I....IYTSTP.EP.SL.........L......
SAMD9L_Poab     EK....C.K.IP.....S..DET.............DVDR...Q.I..........T.S...V....I....IYTSTP.EP.SL.........L......
SAMD9L_Nole     EK....C.N........S..DET.............DVDS...Q.I..........T.S...V....I....IYTSTP.EP.NL.........L......
SAMD9L_Caja     EK....C.N........S..DET.............DVDS..VQ.I..........T.S...V....I....IYTSTP.EP.SL.........L......
SAMD9L_Mamu     EK....C.N........S..DET.............DVDS...Q.I..........T.S...V....I....IYTSTP.EP.SL.........L......
SAMD9L_Loaf     EK....C.N........S..DDM..............VDS...Q.I..........INS...V....F....TSARTP.EP.TL.........L......
SAMD9L_Eqca     EK....C.N........S..DEI....L........DADS..TQ.I..........I.S...V....I....MCTSIP.VP.SL.N...A...L..N...
SAMD9L_Calu     EK....C.N........S....M...K..........VDS..GQ.I..........TES...V....I....IYTSMP.EP.SL.....A...L..N...
SAMD9L_Aime     EK....C.N......L.S...EI..............VDS..GQ.I..........T.S...V....I....IYTSTP.EP.SLD....A...L..N...
SAMD9L_Ereu     EK......N........S..DEM......G......DGDS..TQ.I..........T.S...VP...I....TYI.TP.KQ.SL.........L..NADI
SAMD9L_Orcu     EK...DC.N......L.R..DET..K..........DVYS...Q.I.......T..T.S...V....I....IYTSTP.KP.SL.........L..N...
SAMD9L_Mumu     EKE...C.N......L.G..DTT..K...K.T..DLDAKSRR.Q.I.Y........T.S...V....I....TYT.KYGKP.TV.KN......L..R...
SAMD9L_Crgr     EKE...C.N.....ML.GS.ETT......K.T..DLD.HSRR.Q.I.Y........T.S........I....IYTNKHGKP.SV..N......LV.R...
SAMD9L_Rano     EKEY.DC.N......L.D..DTT..K...K.T..DLDAHSRK.QFI.Y.....T..I.S...V....I....TYTIKRGKP.TV..N......L..R...
SAMD9L_Capo     EK....C.N........G..DEK...........E...NS...Q.I..........T.S........I....IYTSVP.KP.SL..R....Y.L..S...
SAMD9L_Soar     EKE.....N......V.S..DDT.........F....ADS..TQ.I.Y........T.S...VP...I....RHTPSP.VP.SI.........LI.....
SAMD9L_Modo     EKK.E.CR.........E...RT..Q...KH....L.TDS..GQ.I...S.....IS.SDF.VQ......E.T.QR.N.KRGSL.N...N......C.KA
```

```
                  1010      1020      1030      1040      1050      1060      1070      1080      1090      1100
            ....|....|....|....|....|....|....|....|....|....|....|....|....|....|....|....|....|....|....|....|
SAMD9_Hosa  IECGNYCGVRIIHSLIAEFSLEELKKSYHLNKSQIMLDMLTENLFFDTGMGKSKFLQDMHTLLLTRH---RDEH---EGETGNWFSPFIEALHKDEGNEA
SAMD9_Patr  ...................................................................---....---.......................
SAMD9_Gogo  .....................................H.............................---....---.......................
SAMD9_Poab  .................K.................................................---....---.S.....................
SAMD9_Nole  .................K.................................................---....---D......................
SAMD9_Mamu  .................K.................................................---.N..---.......................
SAMD9_Bota  V............P...IR......I..D.D....T.........Y...I.R....E.VQ......Q---.N.N---...M.TL..........E...V.
SAMD9_Susc  .............P...TR......I..N.D....I.........Y...I..D...E..Q......Q---.ND.---.....T..........R....V.
SAMD9_Eqca  V...K........P...DR.....RT....D..............YE..L...R.SEHIQ......Q---.N..---.....TL..............V.
SAMD9_Mylu  V...K........P...IR......I..D.D.....W......V.Y...I.R...F...Q......Q---.I..---.....T...........E...K.
SAMD9_Orcu  L...M........P...SL......N..G.GRC.M..........YE..I........VQ.......---.N..---.D...................D.
SAMD9_Rano  ....S........H...TL......R..N.S..K.VM........Y...I.....Y...Q...I..Q---.N..---.R...................D.
SAMD9_Crgr  ....S.W......HE..SA......R..N.S..E.VMN.......Y.M.I.....F...Q......Q---.N..---.....................D.
SAMD9_Capo  E...T........P...IL.........D.D..............Y...L.R...F...Q......E---.S.Q---.....T.................
SAMD9_Soar  V...C........P...LR......L..Q............R...Y.R....T..I..IQ....I.Q---.N..---....ET.................
SAMD9L_Hosa A.Y.R.T......P...LYC.K..ER....D.C..A.NI.E....Y.S.I.RD..QH.VQ......Q---.KVY---GD..DTL...LM...QNKD----
SAMD9L_Patr A.Y.R.T......P...LYC.K..ER....D.C..A.NI.E....Y.S.I.RD..QH.VQ......Q---.K.Y---GD..DTL...LM...QNKD----
SAMD9L_Gogo A.Y.R.T......P...LYC.K..ER....D.C..A.NI.E....Y.S.I.RD..QH.VQ......Q---.K.Y---GD..DTL...LM...QNKD----
SAMD9L_Poab A.Y.R.T......P...VYC.K..ER....D.C..A.NI.E....Y.S.I.RD..QH.VQ......Q---.K.Y---GD..DTL...LM...QNKD----
SAMD9L_Nole A.Y.R.T......P...LYC.K..ER....D.C..A.NI.E....Y.S.I.RD..QH.VQ......Q---.K.Y---GD..DTL...LM...QNKD----
SAMD9L_Caja S.Y.R.T......P...VYC.K..ER....D.C..A.KI.E....Y.S.I.RD..QH.VQ...V..Q---.R.Y---GD..DTL...LM...QNKD----
SAMD9L_Mamu A.Y.R.T...V..P...LYC.K..ER....D.C..A.NI.D....Y.S.I.RD..QH.VQ......Q---.K.Y---GD..DTL...LM...QNKD----
SAMD9L_Loaf A.Y.R.T......P...IAC.K..E.....D....A.NL.R.K..YVS.I.RE...H.VQ......Q---.R.Y---GD..DTL...L....PNSK----
SAMD9L_Eqca T.YRR.T......P...ISC.K..E...D.D.CK.A.KI.K....Y.S.I.RD..Q..VQ......Q---.K..---GD..DTL...L....QNE.----
SAMD9L_Calu T.Y.R.T......P...ISC.K..EE..D...C..A.KI.N....YVS.I.RE..QH.VQ......Q---.R.Y---GD..DTL.A.L....QNED----
SAMD9L_Aime A.F.R.T......P...I.C.K..EN..N...C..A.KI.K.D..YVS.I.RE..QH.VQ......Q---.K.Y---GD..DTL.A.L....ENE.----
SAMD9L_Ereu A.Y.-FP......P...I.C.K........D..R.A.KL.S....YNS.I..D..QH.VQ......Q---.KGY---G...D-M...L.D-.Q???----
SAMD9L_Orcu S.Y.R.T......P...IHC.K..EEKH....HH.A.KL.N....Y.F.I.RD..QH.VQ......R---.K.Y---GD..DTL...L..D.QNK.----
SAMD9L_Mumu SDY.R.T.I....P...THC.K..EM..RMD.C..A.N..E..VLY.S.L.RD..KY.VQ......Q---.K..---GA..DTL...L..E.QNE.----
SAMD9L_Crgr LDY.R.T.I....P...IHC.K..EM..GMG.C..T.NI.E.KV.Y.S.I.RD..KH.VQ......Q---.K..---GA..DTR...L..E.KNE.----
SAMD9L_Rano SDY.R.A.I....P...IHC.K..EM.HGMD.C..A.N..E..I.Y.S.I.RD..KH.VQ......Q---.K..---GA.IDTL......E.QNEQ----
SAMD9L_Capo A.Y.R.T......P...IHC.K..ER...MD.C..A.NI.N.TV.Y.S.I.RD..QH.VQ......Q---.K.Y---GA..DTL...L..T.KNE.----
SAMD9L_Soar K.Y.-.K..........LCC.K..E...Q.DM.R.A.N..R.K..Y.S.I..D..QH.VQ.......---.K..---GD..DTL...L..D.KNED---I
SAMD9L_Modo .D.KR.DR...V.P...SCC.....ITSEMKR.D.AVNL.K.DTLYSP.IRRD..VRIVQ....K.QKEYSK.TLISGKDMNTP....LMDIK.....DQ
```

```
                     1110      1120      1130      1140      1150      1160      1170      1180      1190      1200
             ....|....|....|....|....|....|....|....|....|....|....|....|....|....|....|....|....|....|....|....|
SAMD9_Hosa   VEAVLLESIHRFNPNAFICQALARHFYIKKKDFGNALNWAKQAKIIEPDNSYISDTLGQVYKSKIRWWIEENGGNGNISVDDLIALLDLAEHASSAFKES
SAMD9_Patr   ....................................................................................................
SAMD9_Gogo   ...........................................................................RS..........N............
SAMD9_Poab   .......G.R.......................D.......E.................................R...........N............
SAMD9_Nole   .......G....K...................................................M.......ER.............N............
SAMD9_Mamu   .......G.................................................................R.R....N......N...Q........
SAMD9_Bota   .KN..RGG.R............T......E...DS..H..N...K...............F.......MDD.ER.RS...GE.SD.....VQ..N.....
SAMD9_Susc   .KN....G.RQ..S........S......ER..NS..H..NE..K.........................-D.R.W...A...AD.....V...N.....
SAMD9_Eqca   .KE....G.R...S.............L.E...SS..K..N...K...H.....................D..K.R.....N.TD..E..VE.TN.....
SAMD9_Mylu   ..K....G.....................ER..TS..H..N...N...N...................T.N.ER.R....A..TE.....V...E.....
SAMD9_Orcu   .KM..EKG.N...K....R.......---------------------------...........L.....D..RGNT..AN..TD..Q..V..AE.....
SAMD9_Rano   .K...H.ATR..D..............L.E...ES..L.....RK.A.N...............L...V.D.IK..I....E.TE.....K...E...D.
SAMD9_Crgr   .KD....ATR.................L.E...K...F.....RT...N....................DD.IR..S..AE..TD...S.V...D...D.
SAMD9_Capo   .KQ..CQG.E..K.......V....Y..RE...N...L...E..K...N.....................DTER.RD......ST.........N.....
SAMD9_Soar   .KE..I.GVD..KQ...............E...ER..E..NK..N...Y....................DD..R.R....A..TM..E..V...N.....
SAMD9L_Hosa  I.K..SAGSR..PQ...............E...NT..D..R...MKA.K..............E.K..LDG.KNCRS.T.N..TH..EA..K..R.....
SAMD9L_Patr  I.K..SAGSR..PQ...............E...NT..D..R...MKA.K..............E.K..LDG.KNCRS.T.N..TH..EA..K..R.....
SAMD9L_Gogo  I.K..SAGSR..PQ...............E...NT..D..R...MKA.K..............E.K..LDG.KNCRS.T.N..TH..EA..K..R.....
SAMD9L_Poab  I.K..SAGSR..PQ...............E...NT..D..R...MKA.K..............E.K..LDG.KNCRS.T.N..TH..EA..K..R.....
SAMD9L_Nole  I.K..SAGSR..PQ...............E...NT..D..H...MKA.K..............E.K..LDG.KNCRS.T.N..TH..EA..K..R.....
SAMD9L_Caja  I.M..SAGSR..PQ...............E...NT..D..C...MKA.K..............E.K..LD..KNCR..T.SQ.TH..EA..K..R.....
SAMD9L_Mamu  I.K..SAGSR..PQ...............E...NT..D..R...MKA.K..............E.K..LDG.KNCRS.T.N..TH..EA..K..R.....
SAMD9L_Loaf  I.N..TAGSIQ.PQ..........Y....E...NT..H..N...K.A.K.......V........K..LD..KTCREVT.K..THF.ET..K..G.....
SAMD9L_Eqca  I.K..V.GSI..PQ...............E.K.NI..E..E..QKRA.K...........H....K..LG..KNCKD.T.N..THF.EA..K..R...K.
SAMD9L_Calu  I.K..IAGAT..PQ..........Y....E.N..T..E..N...KKA.K..............Q.K..LD..KNSTD.T.N...C..EA..N..K...K.
SAMD9L_Aime  I.K..MAGTL..PQ..........Y....E.N.ST..D..NE..KKA.K..............R.K.RLD.MKNAKD.T.N...C..EA..N..K...K.
SAMD9L_Ereu  ????????SN..PQ..........-L..HERN.NT..D..Y...RKA.K.............NEVK..LD..RSCKE.TI...MHC..A..K..R...T.
SAMD9L_Orcu  I.E..VAGCS..PQ...............E.N.ET..E..NL..TRA.K................K..LD..KNSKV.T.K..TH..EA..K..G.....
SAMD9L_Mumu  T.K..IAGSD..PQ...............E.N.ST..V..NL..RKA.K..............ELKQ.LGK.KTC........AYF.EV..K..K.....
SAMD9L_Crgr  T.KI.TVGSD..PQ...............E.N.ST..V..NL..RKA.K..............E.KQ.LGK.ENCRL......TH..EI..K.TK.....
SAMD9L_Rano  T.K..TAGSD..PQ.............L.E.N.ST..V..N...RKA.K..............E..Q.L------------------KV..K..K.....
SAMD9L_Capo  .KK..TRGSS..PE...............E.N.ND..T..NL..ERA.K..............E.KC.-------------------ET.GK.AR..R..
SAMD9L_Soar  ..N..TAGSN..PK.................N.DT..F..T...TKA.N..............E.K..L.K.KNCKI..AS..TYF.EA..K..K...M.
SAMD9L_Modo  ..R..T.GTI..KDH.L.Y.........NENN.EA.IFY.EE..KRSVR..FV..........QLIFCNNKISQGKS.PCQE.EY..NI..K..N...D.
```

```
                       1210      1220      1230      1240      1250      1260      1270      1280      1290      1300
                  ....|....|....|....|....|....|....|....|....|....|....|....|....|....|....|....|....|....|....|....|
SAMD9_Hosa        QQQSEDREYEV--KERLYPKSKRRYDTYNIAGYQGEIEVGLYTIQILQLIPFFDNKNELSKRYMVNFVSGSSDIPGDP---NNEYKLALKNYIPYLTKLK
SAMD9_Patr        ...........--.................................................................---...................
SAMD9_Gogo        ...........--.................................................................---D..................
SAMD9_Poab        ...........--.....L...........................................D....I..........---...................
SAMD9_Nole        ...........--.........................................K.......D....I..........---...................
SAMD9_Mamu        .....Y.....--.....Q...........................................D........G.....Q---.D..Q..............
SAMD9_Bota        ..........G--...F.Q..........T...L..............F.............D.I..I...K....EA---...F..V...F.S...N..
SAMD9_Susc        .R.........--...F.Q...........................................D.I..I..TT......---...F..V.N.F........
SAMD9_Eqca        .......Q..G--...F.Q...................A...............S.......D.I..I.........S---S..F......F.....N..
SAMD9_Mylu        .....YK.D.A--M..FNQ.......................A...................D.I..I..N.......---...F......F.......R
SAMD9_Orcu        .R........G--.....Q..........................................KD.......T......S---...F.SV...F.....N.R
SAMD9_Rano        ...T......G--..SV.Q............................K.L..NS......RQV.I..I..NC.....L----..F.SV...F.S...N.Q
SAMD9_Crgr        .R.T......G--....NQ.........................K...........D.....D.I..I..NR.....L----..F.I....F.....N.Q
SAMD9_Capo        .R........G--....GQ...........S..H........A...........K.....R.D.I..I..N......L----..F......F.....T..
SAMD9_Soar        .R...YK.R.A--.GKFHQ...................................S....F.KD.I..I..T......S---...F....S.F.S...N.R
SAMD9L_Hosa       .R.TDSKN..T--ENWSPQ..Q....M..T.CFL...............T...HKE.....KH..Q.L..KWT..P..---R..CY...SKFTSH.KN.Q
SAMD9L_Patr       .R.TDSKN..T--ENWSPQ..Q....M..T.CFL...............T...HKE.....KH..Q.L..KWT..P..---R..CY...SKFTSH.KN.Q
SAMD9L_Gogo       .R.TDSKN..T--ENWSPQ..Q....M..T.CFL...............T...HKE.....KH..Q.L..KWT..P..---R..CY...SKFTSH.KN.Q
SAMD9L_Poab       .R.TDSKN..T--ENWSPQ.YQ....M..T.CFL...............T...HKE.....KH..Q.L..KWT..P..---R..CY...SKFTSH.KN.Q
SAMD9L_Nole       .R.TDSKN..T--ENWSPQ..Q....M..T.CFL...............T...HKE.....KH..Q.L..KWT..P..---R..CY...SKFTSH.KN.Q
SAMD9L_Caja       .R.TDSKNC.T--ETWSPQ..Q...G...T.CFL...............T...QKE.....ENP..Q.L..KWT..P..---K...Y...SKFTSH.KH.Q
SAMD9L_Mamu       .R.TDSKN..T--E.WSPQ..Q....M..T.CFL...............T...HKE....IKT..Q.L..KWT..P..---R...Y...SKFTSH.KT.Q
SAMD9L_Loaf       .R.NDSKD..P--EVWPLQ.YQ.K..M..T..FL........S.E....T.L.HKE...AQKPL.Q.L..IG..LA..---E...H.T...FTS..QN.R
SAMD9L_Eqca       .E.TDRKN..T--EAW.PQ..Q.K.VM..T..FL........A......T.C.HKE.....KS..E.L..KGN.LTNS---KS..Y.V.RKFTS..QN.Q
SAMD9L_Calu       .E.T.RKG..TWADTWAKQTLQ.K.....T..FF........A......T.C.LKQ.....KA..E.L..KGM..TN.---KC..Y...NKFTS..EN.Q
SAMD9L_Aime       .E.A.RKAC.T--EPWGPQ.LQ.K.....T..FV........A......T.C.LKE..F..KA.AD.L..KGMT.TN.---KC..Y...SKFTS..EN.Q
SAMD9L_Ereu       .E.TDKKDS.T--EAWSTQ..LK..EM..T..FL........A......T.C.HKE.K..Q.HT.E.L..KGN..EHS---KGK.Y..ISKFTSH.RN.Q
SAMD9L_Orcu       ...TDSKN..T--EAWSAQ..Q.......T..FF...............T...HKD.VSFLKP..Q.L..KG...F.A---R...Y...SKFTSL.QN.Q
SAMD9L_Mumu       .N..DSKD.GT--EAWSPQN.Q.....F.T..FF.......D...L...T.L.HKE..I..ES.AE.L..KGT.LS..---KG..CVV.SKFTSL.QN.H
SAMD9L_Crgr       .E.TDNKD..T--EAWSPR..Q.......T..FF.......D.......T.P.HKES.IAIEA..Q.L..KGT.S-..---KD..GAV.SKFASH.QN.Q
SAMD9L_Rano       .E.TDSKD..I--EVWSPQ..Q.......T..FF.......D...L...T.L.HKE..M..ES.AQ.L..KGT..P..---KG..YVV.NKFKSF.QN.Q
SAMD9L_Capo       ...TDSKD..T--EAWSPQ.PQ.......T..FL....S.........T...HKED.S.LES..Q.L..KEN.SP..---K...Y...SKFTAH.QS.Q
SAMD9L_Soar       .E.TDKKDC.R--GVW.QQRFR.K..M..T..FL.......FA........C.HKE.D..EKR..K.LL.KVNV.T..---K.D.Y...RQFTS..QN.Q
SAMD9L_Modo       ..YN.NKDDKT--G--Q.HRY..S-....TSC.F......IQI..L.EKVFL..ER..SP.GL.GQLLR.N.GVLLN.LIQR.M.YPVI.KH.D..NA.Q
```

```
                        1310      1320      1330      1340      1350      1360      1370      1380      1390      1400
               ....|....|....|....|....|....|....|....|....|....|....|....|....|....|....|....|....|....|....|....|
SAMD9_Hosa     FSLKKSFDFFDEYFVLLKPRNNIKQNEEAKTRRKVAGYFKKYVDIFCLLEESQNNTGLGSKFSEPLQVERCRRNLVALKADKFSGLLEYLIKSQEDAIST
SAMD9_Patr     ....................................................................................................
SAMD9_Gogo     ........................................................D.R..............S..........................
SAMD9_Poab     .....................................................K-.DP..........................................
SAMD9_Nole     ..........................................I....VS.....-KD...............SS....................A..V..
SAMD9_Mamu     ....................................V.....T......G...D-I...........I....KS..........................
SAMD9_Bota     ..........................................A...GPS..L..-KDF...L.V.....LY..S.EV...............A....VH.
SAMD9_Susc     ...........D....................K...V.....A...GPS.D..S-KY....L.L.....Q...S.EV...............N.K..A..
SAMD9_Eqca     C...RC.....D.................R.......H........GPSV....-KD..A.L.I.....LY.KS.EV...........N...N.....R.
SAMD9_Mylu     S..........D........S......QS.............A...GPF..L..-RD...QL.L..R..LY..S.EV..................I..N.
SAMD9_Orcu     ...........D................T.......TC..R.L...GPSA.ILS-K.....I.LS...QE...S.E..............V....H....
SAMD9_Rano     S..........D...........E.Y....S.....M......E..GP.A.L..-QNFR..L.L......T..S.E.................PK.TV.S
SAMD9_Crgr     S...RA.....D.............Y..S.C.S..V....R.....S.......-QNFR..LTL......I..T.E.................PK..V.S
SAMD9_Capo     S...DA.....D.............H.........VV.....I...GPSG..L.-RD.EL.L.L......NQ.S.E................T.-..V.S
SAMD9_Soar     ....N......D..........M..SD--.............G...D.FNGT.S-KDF..NI.LS.KI.LF.SK.EV.............V.N.NSP.E.
SAMD9L_Hosa    SD..RC....ID.M....M.YTQ.EIA.IMLSK..SRC.R..TEL..H.D----PCL.Q..E.QL..E.N..KK.E..R..R.A......NPNYK..-T.
SAMD9L_Patr    SD..RC....ID.M....M.YTQ.EIA.IMLSK..SRC.R..TEL..H.D----PCL.Q..E.HL..E.N..KK.E..R..R.A......NPNYK..-T.
SAMD9L_Gogo    SD..RC....ID.M....M.YTQ.EIA.IMLSK..SRC.R..TEL..H.D----PCL.Q..E.QL..E.N..KK.E..R..R.A......NPNYK..-T.
SAMD9L_Poab    SD..RC....ID.M....M.YTQ.EIA.ITLGK..SRC.R..TEL..H.D----PCL.Q..E.QL..E.N..KK.E..R..R.A......NPNYK..-A.
SAMD9L_Nole    SD..RC....ID.M....M.YTQ.EIT.IMLSK..SRC.R..TEL..H..----PCL.Q..E.QL..E.N..KK.E..R..R.A......NPNYK..-A.
SAMD9L_Caja    SD..RC....ID.M....M.YAQ.EIV.VMLSK..SRC.R..TEL..H.D----SDQ.QN.G.QL..E.N..KK.E..R..R.A......NPKYK.SATI
SAMD9L_Mamu    SD..RC....ID.M....M.YTQ.EIV.ITLSK..SRC.R..TEL..H.D----PCL.R..E.QLF.E.N..KK.E..R..R.A......NPNFKN.AT.
SAMD9L_Loaf    LD...C....VD.M....T..TH.EIV.SILSK..SR.YR..TE...H.G----LDP.Q..E--L..E.NY.KS.E..R..R........NPNHK..AN.
SAMD9L_Eqca    SD..RC.E..AD.M...EMK.TQ.ETA.LSLNK.ISRC.R..MEL..H.D----LGV.Q.RE.QL.KE.N..KA.E..R..R........NSNHKEVAT.
SAMD9L_Calu    SD..RC.....D.L....MK.TQ.ETG.ISLSK.ITRC.....EL..H.D----SGP.HR-E.QL..E.N..KA.E..R..R........NPNHRE.ATN
SAMD9L_Aime    SD..RC....ND.I...QM..TQ.ETV.ISLNK.ITRC.R..GEL..C.D----LGL.Q..E.QFF.E.SF.KG.E..R..R........NPNHRE.ATN
SAMD9L_Ereu    SD..RC....AD.M.F..T..TQ.ETA.IILSK..SR..RQ.IEL..HSD----VGI.QN.G.QL..E.YY.KS.E.KR..R........NP.HQETGNI
SAMD9L_Orcu    SN..RC....ID.M....M..TQ.EMA.ILLSK..SRCY...R.L..I.D----PSP.H..E.QL.LE.N..KK.E.WR..R........SLNHKE.T..
SAMD9L_Mumu    SD.ERC.H..GD.MGF.....TP.ELT.LLLSK..SRC.....EL..H.D----TNLVQG.EDLL..K.N..KRIQ.WR..T........NPNHKE.-NN
SAMD9L_Crgr    SE..RC....LD.IG.....ITP.ETT.LSLIK...R.....AGL..RMN----TNL.QG.ENVL..E.N..KRI..WR..T........NPNHKEV-DN
SAMD9L_Rano    SD..RC....LD.MG......TP.ELT.LSLSK..SRC....A.L..Q-.-------.QG.EDLL..E.N..KRIK.WR..T........NPNHKE.-NN
SAMD9L_Capo    SD.I.C.H..SD.M....T..FQ.EMT.L.LSK..IFCY.T.TKL..H.D----S.P.QG.E.QL..E.N..KR.E.WR..R........NSNHK..ATI
SAMD9L_Soar    PD..RC....AD.M.F..T.TVH.EIT.ISL.K.IGHC.MS.IS..NH..----LGTTQAQENLL..E.N..KR.EKCR..R..L.....NPNYK..AT.
SAMD9L_Modo    KR.ETI.NNLQD..SF..LKD.E.EIV.E.NQI.MEKL..Q.LS..SNS.-------IRPSM----VTSV.W.E.EISN.AR.....K..T.TH.N.EME
```

```
                   1410      1420      1430      1440      1450      1460      1470      1480      1490      1500
              ....|....|....|....|....|....|....|....|....|....|....|....|....|....|....|....|....|....|....|....|
SAMD9_Hosa    MKCIVNEYTFLLEQCTVKI----QSKEKLNFILANIILSCIQPTSRLVKPVEKLKDQLREVLQPIGLTYQFSEPYFLASLLFWPENQQLDQHSEQMKEYA
SAMD9_Patr    ...................----..................K..........................................................
SAMD9_Gogo    .EY..........R.....----..................K...........................................Y..............
SAMD9_Poab    .E...K.......R.A...----..................K...........................R.......................K.....S
SAMD9_Nole    .EN..DK....S.......----..................K...........................R.......................G......
SAMD9_Mamu    .ED...K.A..........----E.................K...T......................HR.......................Q...A..
SAMD9_Bota    .ED.MDK....F.......----LI...Q.........Y..K...KI...IK........L..QT.MS.RYP...................D.RL.EK..
SAMD9_Susc    .ED.M.K.S..F.....RM----L...Q..........Y..K...KI.M.IK...E.......QAETICRQ....................D.K..EK..
SAMD9_Eqca    .DD...K....F..G....----.P...Q.........Y..K...KI...IR...........Q.....R.................H...D.K..EK..
SAMD9_Mylu    .ED..KK....F...A.R.----.Q...Q.........Y..K...KI.M.TK........I..Q..PS...................K...D.K..EKH.
SAMD9_Orcu    .EN..HK....F..S.E..----.L...Q............K...KS..TIK......Q.I..QV..NCR.....................D.K..EK..
SAMD9_Rano    .ED...K........AI..----.Q...Q.....S......N.I.KF...IK...E....I..............................D.K..EK..
SAMD9_Crgr    .EG...Q..S.....VI.T----.Q...Q............N.A.K.....K...E....I..............................D.K..EK..
SAMD9_Capo    .EY...T.I..FD..I.RM----HL...Q............K.A.K....IK...EH..DI..QV.P.HR.....................D.KE.EK.V
SAMD9_Soar    .ES..EQ.S.I..H.AAS------T...Q.......V.Y..K...KF.S..K...E.......-V.TN.R.P.............T.N...D.K..EK..
SAMD9L_Hosa   .ES.....A...Q.NSK.P----MTN..Q.S.........LK.N.K.IQ.LTT..K.......FV..SH.YPG.....C........E...D.KLIEK.V
SAMD9L_Patr   .ES.....A...Q.NSK.P----MTN..Q.S.........LK.N.K.IQ.LTT..K.......FV..SH.YPG.....C........E...D.KLIEK.V
SAMD9L_Gogo   .ES.....A...Q.NSK.P----MTN..Q.S.........LK.N.K.IQ.LTT..K.......FV..SH.YPG.....C........E...D.KLIEK.V
SAMD9L_Poab   .ES.........Q.NSK.H----MTN..Q.S.........LK.N.KFIQ.LTT..K.......FV.PSH.YPG.....C........E...D.KLIEK.V
SAMD9L_Nole   .ES.....A...Q.NSK.R----MTN..Q.S.........LK.K.K.IQ.LTT..K.......CV..SH.YPG.....C........E...D.KLIEK.V
SAMD9L_Caja   .ES.....A...H.NSK.C----MTN..Q.S.........LK.N.KSIQ.LNT..K.......FV..SH.YPN.....C........E...D.KLIEK.V
SAMD9L_Mamu   .EI..D..A...K.NSN.R----MTN..Q.S.......N.LK.S..FIQ.LTM..K.......FV..SH.YPD.....C........E...D.KLIEK.V
SAMD9L_Loaf   VER...Q.AL.FQ.NLN.K----LI...Q...........LK.S.KYIR.FNI..K......ELVEP.H.YPD.....C........E...D.KL.EK.V
SAMD9L_Eqca   .ENV..K.....Q.NPN.Q----LTR..Q.........N.LK...KSIQ.LS...K..Q...HIV.PRH.YPD.....C....L...E..ED.QL.EK.V
SAMD9L_Calu   .EN...K.N...Q.NPK.Q----MT...Q.........N.LK.H.KFIQ.LPI..K......LS..PSH.YPN.....C.....Q..E..ED.KF.EK.V
SAMD9L_Aime   .EN...K.S...R.NPN.Q----LT.............N.LK.H.KSIQ.LSI..KL...A.LS..PS..YPD.....C........E..ED.KL.EK.V
SAMD9L_Ereu   .EN..E..A...Q.NPN.R----MI...Q...........LK.N.KFIQ.LDT..K.......LVEINR.YPD.....C.....D..E...D.KL.EK.V
SAMD9L_Orcu   .EN..D..A...K.NPN.R----LT...Q...........LK...KSIQ.LNM..K.......LV..HH.Y.D.....C........E...D.KL.EK.V
SAMD9L_Mumu   IEN..GN.....QDILN.QLSKVLT.DIQ...........LK.S.KYIL.FST..KK......IV...HSYPD.....C.......KE..ED.TLIEK.V
SAMD9L_Crgr   .ES..KD.....QHSLS.RVTKGLT..TQ...........LK.S.KYIL.FNT..TK....ELV..GHPYPD.....C.......KE...D.TLIEK.V
SAMD9L_Rano   .EN..EH.....QHTLN.QLSKALI.DTQ...........LK.S.KHIL.FST..KK......IV...HSYPD.....C.......KE...D.SLIEK.V
SAMD9L_Capo   .EN.....A...Q.NPSIR----SI...Q...........VK.N.KFIQ.LSI..Q.......LV..N..YPD.....C........K...D.KL.EK.V
SAMD9L_Soar   IES..KD.....Q.NLQNP----MR...Q...........LN.N.VFIE.LDT..EL......LAK.DHSYPD.F...C.......EV...D.QLLEK.V
SAMD9L_Modo   .ES..DR.ML.VQ.ASSM-----KTRA.Q.........N.LK.N.KIR..LDE..AM.Q...LQV..HSSYPD.....T........H.N.N.TEIID.V
```

```
                       1510      1520      1530      1540      1550      1560      1570      1580      1590      1600
               ....|....|....|....|....|....|....|....|....|....|....|....|....|....|....|....|....|....|....|....|
SAMD9_Hosa     QALKNSFKGQYKHMHRTKQPIAYFFLGKGKRLERLVHKGKIDQCFKKTP-DINSLWQSGDVWKEEKVQELLLRLQGRAENN-CLYIEYGINEKITIPITP
SAMD9_Patr     .............................................E...-...............................-..................
SAMD9_Gogo     .................................................-...............................-..................
SAMD9_Poab     ...E.........................E..K............E...-...............................-..................
SAMD9_Nole     ...E............................K............E..L-...............................-..................
SAMD9_Mamu     ...E............................K............E...-..............A................-..................
SAMD9_Bota     SS.E...R......Y..............NSVN............E..A-...F..........K...K.....K..VG..-..H...............
SAMD9_Susc     RS.E...R......C..............NNMN..I.R......C...AA..K.F.........KE..K..F..K......-..................
SAMD9_Eqca     RS.E...R......Y..............NNMT............RN.S-..............RN........K......-.......V.D........
SAMD9_Mylu     RS.Q...R......Y..............NNMN...........YG..L-...IW....E....K.........K......-..........R.......
SAMD9_Orcu     .S.E...R......Y..............NNMN............E..L-..............R................-.......VSD...L....
SAMD9_Rano     ...EK..R....RLY..............NSRN..I......EN.ENLS-....W.HT.K..E.K...D..V..P......-................K.
SAMD9_Crgr     W..ET..R....R.Y...........E..NNRN..I......EN.E.VS-....W.H..K..E.K...D............-N............Q...
SAMD9_Capo     .S.EK........................TNMN.........E..GEMS-...F.....K..E.K...D............EK.F...............
SAMD9_Soar     RL.ET..L.K.R..Y.............A.NIN............G..--.......N....R.K...D.....N......-.V..D...K...I.....
SAMD9L_Hosa    SS.NR..R....R.C.S..ASTL.Y...R.G.NSI...A..E.Y.D.AQ-NT....H......KNE.KD..R..T.Q..GK-LISV...TE...K..VIS
SAMD9L_Patr    SS.NR..R....R.C.S..ASTL.Y...R.G.NSI...AE.E.Y.D.AQ-NT....H......KNE.KD..R..T.Q..GK-LIS....TE...K..VIS
SAMD9L_Gogo    SS.NR..R....R.C.S..ASTL.Y...R.G.NSI...A..E.Y.D.AQ-NT....H......KNE.KD..R..T.Q..GK-LIS....TE...K..VIS
SAMD9L_Poab    SS.NR..R....R.C.S..ASTL.Y...R.G.NSI...A..E.Y.D.AQ-NT....H......KNE.KD..R..T.Q..GK-LIS....TE...K..VIS
SAMD9L_Nole    SS.NR..R....R.C.S..ASTL.Y...R.G.NSI...AE.E.Y.D.AQ-NT....H......KNE.KD..R..T.Q..GK-LIS....TE...K..V.S
SAMD9L_Caja    SS.NR..R......C.S..ASTL.....R.G.NSI...AE.E.Y.G.VH-NT...........KNE.KD..R..I.Q..GK-LIS....TK...K..VIS
SAMD9L_Mamu    SS.NR..R....R.C.S..ASTL.Y...RRG.NSI...AE.E.Y.D.VQ-NT....H......KHE.KH..C..I.Q..GK-LIS....TE...K..VIS
SAMD9L_Loaf    SS.NR..GR..R..C.S..ASTL.Y...S.G.HSI...AE.E.Y.S.EQ-NT...........KNE.KD..R..T.Q.VGK-LIS....TE...R..VIS
SAMD9L_Eqca    SS.NR...R..SN.C.SR.ASTV.Y...K.G.HS....A..E.Y.G.VQ-NT.....N....EKKE.KD..C..T.Q..GK-RIS....TK...K..VIS
SAMD9L_Calu    SS.NRN..R..RS.C.S..ASTL.Y...Q.G.HS..C.AE.E.Y.N.AQ-NTS..S..A..C.K-..KD..C..T.Q..GK-LISM...TEK.VK..VI.
SAMD9L_Aime    SS.NRT..R..RS.C.S..ASTL.Y...K.G.HS..C.AE.E.Y.N.AQ-NV...SP......KKE.KD..C..T.Q..GK-LISM...TEK..K..VI.
SAMD9L_Ereu    SS..K..NRH.RL.C.S..ASTL.Y..NR.G.NS....AE.E.YIS.VK-NT..F....H...K.E.KG..R..N....GK-LILM...TE...K..VIS
SAMD9L_Orcu    SS.NRT.R....R.C.S..ASTF.Y...R.GFNG..L.AE.E.Y.S.AQ-N............KKE.KD..H..V.Q..GK-LIS....TE...K..V.S
SAMD9L_Mumu    SS.NR..RR.....C.SR..STL.Y..QK.G.NS....AE.ERY.SEVQ-.S..F.H..V..EKRE.KD..RL.D.Q..GK-LISL...TEA..K..V.S
SAMD9L_Crgr    SS.NR..RR.....C.S.K.STL.Y..QK.G.HS....AE.ERYVSEVQ-NS..F....V..EKRE.KD..RL.D.Q..GK-LISV...TEA..K..V.S
SAMD9L_Rano    SS.NR..RR.....C.SR..STL.Y..QK.G.NS....AE.ERY.SEVQ-.S..F....V..EKGE.KDI.RL.D.Q..GK-LISL...TEA..K..V.S
SAMD9L_Capo    VS.NR..R....R.C.S..ASTF.Y..TK.G.NS....AELEKHIS..K-NT..F.....I..T-E.KH..H..T.Q..GK-LIS...............
SAMD9L_Soar    SS..K..NR..RR.C.S..ASTL.Y..N..G.KG....TE.E.Y.S.V.-NTSI...N.....KKE.KS..C..T.Q..GK-LIS.Q..TE...N..VIS
SAMD9L_Modo    T..RKT.WD..GQ.C.G...FTH.Y..NDRG.N....R...E...QDAS-.VSI...R....R....KD.....S..V..D-LI..DC.SK.NAE..VR.
```

```
                    1610      1620      1630
             ....|....|....|....|....|....|....
SAMD9_Hosa   AFLGQLRSGRSIEKVSFYLGFSIGGPLAYDIEIV
SAMD9_Patr   ..................................
SAMD9_Gogo   ..................................
SAMD9_Poab   ..................................
SAMD9_Nole   ..................................
SAMD9_Mamu   T.................................
SAMD9_Bota   .................................I
SAMD9_Susc   ............D.........T.........VI
SAMD9_Eqca   ..F..............................I
SAMD9_Mylu   ..W..............................I
SAMD9_Orcu   ..W.........D....................I
SAMD9_Rano   THF......................L.....K..
SAMD9_Crgr   TH.......................L.....K..
SAMD9_Capo   ..F............................Q.L
SAMD9_Soar   T.F............................QVI
SAMD9L_Hosa  VYS.P.....N..R.........E........VI
SAMD9L_Patr  VYS.P.....N..R.........E........VI
SAMD9L_Gogo  VYS.P.....N..R.........E........VI
SAMD9L_Poab  VYS.P.....N..R.........E........VI
SAMD9L_Nole  VYS.P.....N..R.........E.......KVI
SAMD9L_Caja  VYS.P.....N..R.........E.......QVI
SAMD9L_Mamu  VYS.P.....N..R.........E........VI
SAMD9L_Loaf  VYS.P....GN..R.........E........VI
SAMD9L_Eqca  VYS.P.Q...N..R........ME........VI
SAMD9L_Calu  VYS.P....GN..R........ME.LQ..E...I
SAMD9L_Aime  VYS.P....GN..R.....A...E..Q......I
SAMD9L_Ereu  VYS.P.....N.QR........ME........VI
SAMD9L_Orcu  VYS.P.....N............E........V.
SAMD9L_Mumu  VYSAP.....N..R.........E.....G.KVI
SAMD9L_Crgr  VYS.P.....N..R.V.......E.....G.KVI
SAMD9L_Rano  VYS.P.....N..R.........E.....G.KVI
SAMD9L_Capo  ..F............................Q.L
SAMD9L_Soar  VYS.P.....H.QS........ME........LI
SAMD9L_Modo  .V.....R.Q.M.R........TA..V....KNI
```
