# Supplementary material for: Evolution and divergence of the mammalian SAMD9/SAMD9L gene family
Source: BMC Evol Biol. 2013 Jun 12;13:121. doi: 10.1186/1471-2148-13-121 (PMC3685527; doi:10.1186/1471-2148-13-121)
Supplement: Additional file 2: Table S1 — Detection of recombination breakpoints from SAMD9 and SAMD9L genes alignment using GARD analysis. SAMD9 and SAMD9L complete coding sequences were aligned together and the software GARD was used to look for any evidence of recombination. Three breakpoints were identified, but only one was strongly supported by the Kishino-Hasegawa (KH) test. [file 1471-2148-13-121-S2.pdf]

Table S1: Detection of recombination breakpoints from *SAMD9* and *SAMD9L* genes alignment using GARD analysis

***SAMD9+SAMD9L* alignment**				
Breakpoint	LHS[a] Raw *p*	LHS[a] adjusted *p*	RHS[b] Raw *p*	RHS[b] adjusted *p*
1179	0.94650	1.00000	0.00010	0.00060
2404	0.00010	0.00060	0.40590	1.00000
**4755**	**0.00010**	**0.00060**	**0.00010**	**0.00060**

[a]LHS: segment to the left of the breakpoint
[b]RHS: segment to the right of the breakpoint
