# Supplementary figures and images for: Evolution and divergence of the mammalian SAMD9/SAMD9L gene family
Source: BMC Evol Biol. 2013 Jun 12;13:121. doi: 10.1186/1471-2148-13-121 (PMC3685527; doi:10.1186/1471-2148-13-121)

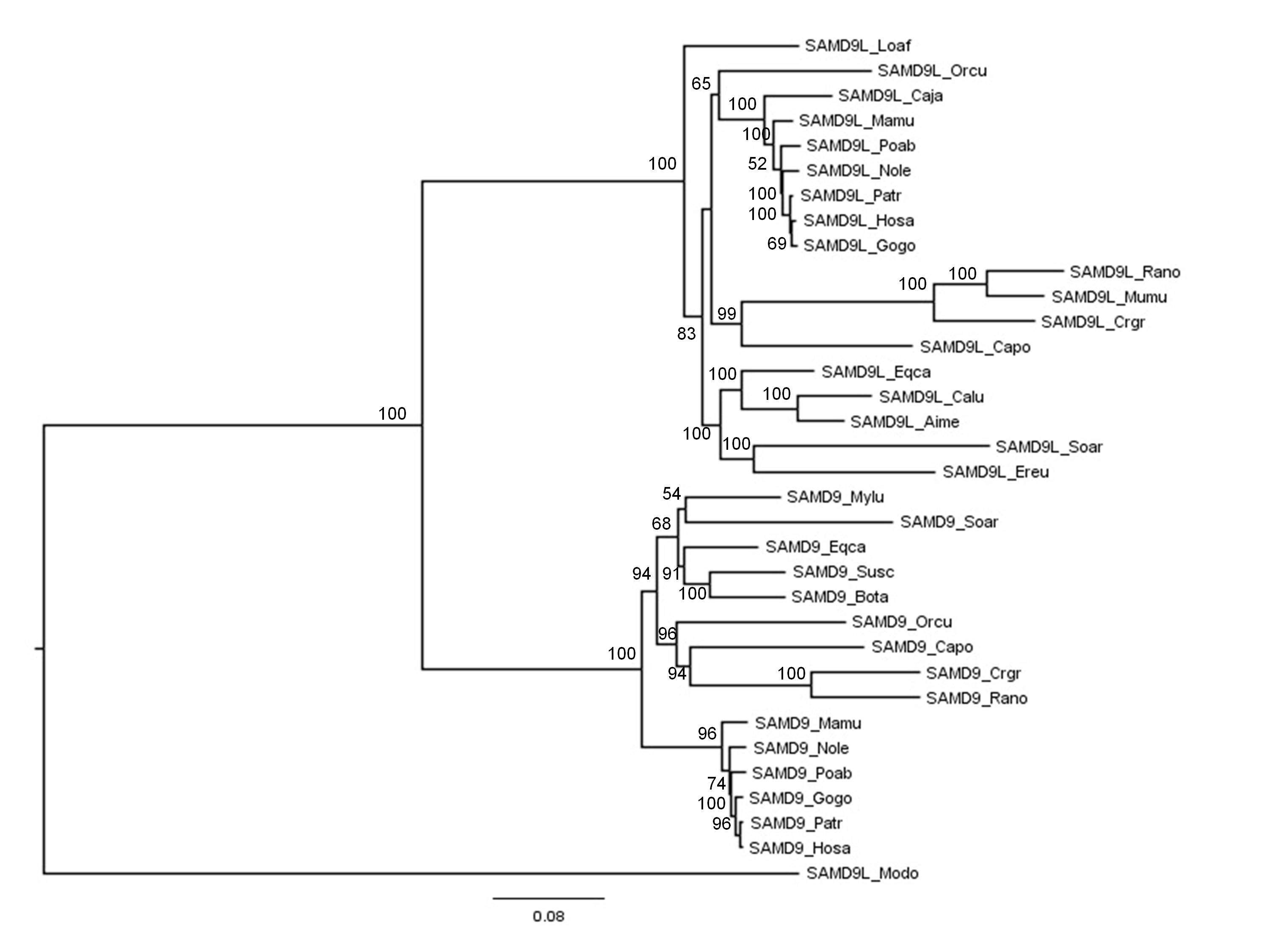

Supplement: Additional file 3: Figure S2 — Mammalian SAMD9 and SAMD9L genes estimated Maximum Likelihood tree without testing recombination. A phylogenetic tree was estimated for the mammalian SAMD9 and SAMD9L genes alignment using the Maximum Likelihood (ML) method and under the GTR+I+G nucleotide substitution model. The analyses were performed with 1,000,000 generations and 1,000 bootstrap searches. The bootstrap values are indicated on the branches. The abbreviations correspond to the following species common names: Aime - Giant panda; Bota - Cow; Caja - Common marmoset; Calu - Domestic dog; Capo - Domestic Guinea pig; Crgr - Chinese hamster; Eqca - Horse; Ereu - West European hedgehog; Gogo - Western gorilla; Hosa - Human; Loaf - African bush elephant; Mamu - Rhesus monkey; Modo - Grey short-tailed opossum; Mumu - House mouse; Mylu - Little brown myotis; Nole - Northern white-cheeked gibbon; Orcu - European rabbit; Patr - Common chimpanzee; Poab - Sumatran orangutan; Rano - Brown rat; Soar - Common shrew ; Susc - Pig. To access the species scientific names, the list of abbreviations should be consulted. [file 1471-2148-13-121-S3.jpeg]

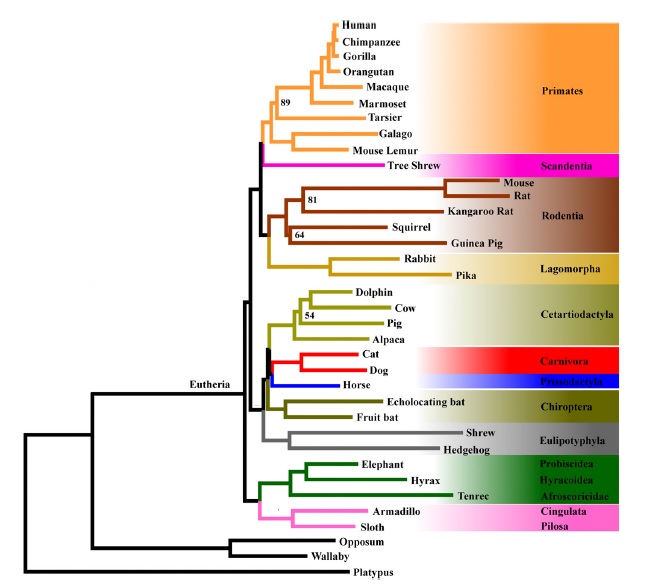

Supplement: Additional file 4: Figure S3 — Evolutionary relationships of eutherian mammals. Placental mammals’ evolutionary relationships tree retrieved and adapted from Song et al.[15]. [file 1471-2148-13-121-S4.jpeg]

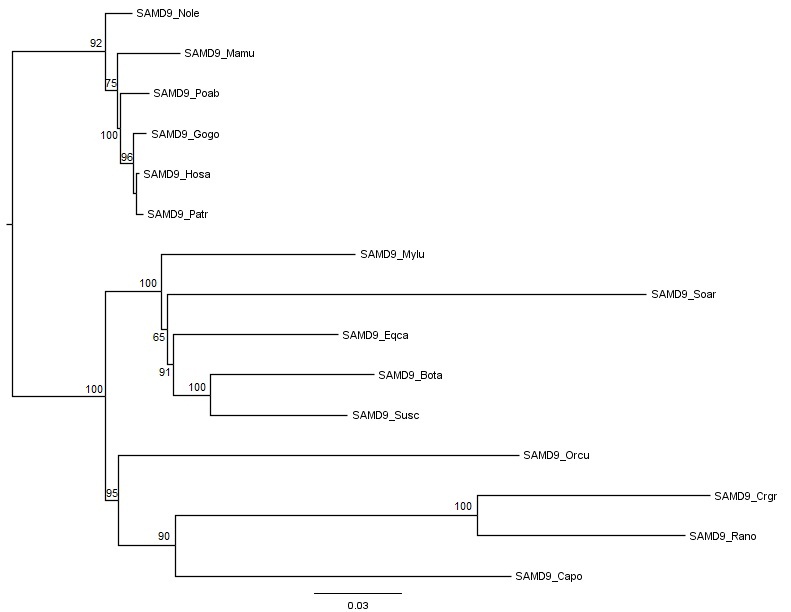

Supplement: Additional file 7: Figure S6 — Mammalian SAMD9 gene estimated Maximum Likelihood tree. The phylogenetic tree of mammalian SAMD9 gene alignment was estimated using the Maximum Likelihood method and the nucleotide substitution model TVM+G. The analyses were performed with 1,000,000 generations and 1,000 bootstrap searches. The bootstrap values are indicated on the branches. The abbreviations correspond to the following species common names: Hosa - Human; Patr - Common chimpanzee; Gogo - Western gorilla; Poab - Sumatran orangutan; Nole - Northern white-cheeked gibbon; Mamu - Rhesus monkey; Bota - Cow; Susc - Pig; Eqca - Horse; Mylu - Little brown myotis; Orcu - European rabbit; Rano - Brown rat; Crgr - Chinese hamster; Capo - Domestic Guinea pig; Soar - Common shrew. To access the species scientific names, the list of abbreviations should be consulted. [file 1471-2148-13-121-S7.jpeg]

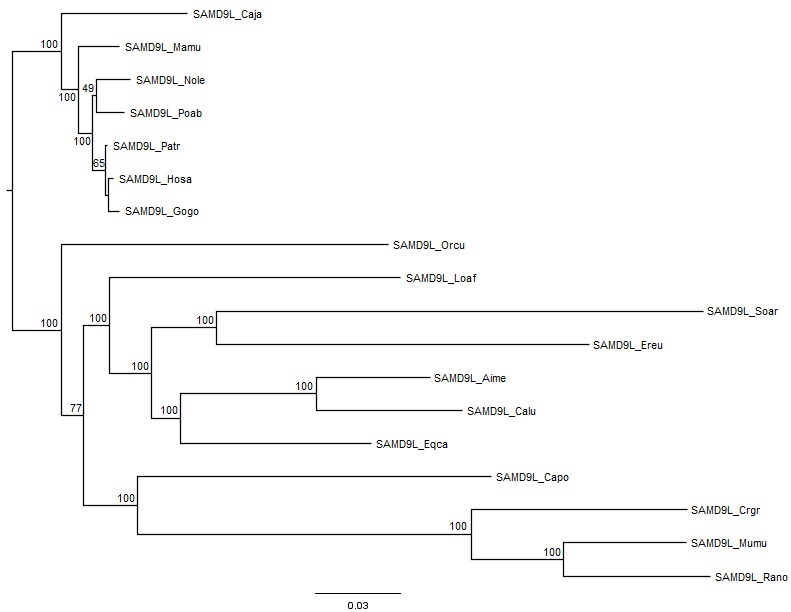

Supplement: Additional file 8: Figure S7 — Mammalian SAMD9L gene estimated maximum likelihood tree. The phylogenetic tree of mammalian SAMD9L gene alignment was estimated using the Maximum Likelihood method and the nucleotide substitution model GTR+G. The analyses were performed with 1,000,000 generations and 1,000 bootstrap searches. The bootstrap values are indicated on the branches. The abbreviations correspond to the following species common names: Hosa - Human; Patr - Common chimpanzee; Gogo - Western gorilla; Poab - Sumatran orangutan; Nole - Northern white-cheeked gibbon; Caja - Common marmoset; Mamu - Rhesus monkey; Loaf - African bush elephant; Eqca - Horse; Calu - Domestic dog; Aime - Giant panda; Ereu - West European hedgehog; Orcu - European rabbit; Mumu - House mouse; Crgr - Chinese hamster; Rano - Brown rat; Capo - Domestic Guinea pig; Soar - Common shrew. To access the species scientific names, the list of abbreviations should be consulted. [file 1471-2148-13-121-S8.jpeg]
