# Supplementary material for: Evolution and divergence of the mammalian SAMD9/SAMD9L gene family
Source: BMC Evol Biol. 2013 Jun 12;13:121. doi: 10.1186/1471-2148-13-121 (PMC3685527; doi:10.1186/1471-2148-13-121)
Supplement: Additional file 5: Figure S4 — Mammalian SAMD9 deduced protein sequences alignment. SAMD9 deduced protein sequences from fifteen species were aligned with ClustalW implemented in BioEdit. The abbreviations correspond to the following species common names: Hosa - Human; Patr - Common chimpanzee; Gogo - Western gorilla; Poab - Sumatran orangutan; Nole - Northern white-cheeked gibbon; Mamu - Rhesus monkey; Bota - Cow; Susc - Pig; Eqca - Horse; Mylu - Little brown myotis; Orcu - European rabbit; Rano - Brown rat; Crgr - Chinese hamster; Capo - Domestic Guinea pig; Soar - Common shrew. To access the species scientific names, the list of abbreviations should be consulted. Codons are numbered according to human SAMD9 protein. “?” represents undetermined codons; “.” represents identity with the reference sequence of human SAMD9 protein. [file 1471-2148-13-121-S5.pdf]

```
                    10        20        30        40        50        60        70        80        90       100
           ....|....|....|....|....|....|....|....|....|....|....|....|....|....|....|....|....|....|....|....|
SAMD9_Hosa MAKQLNLPENTDDWTKEDVNQWLESHKIDQKHREILTEQDVNGAVLKWLKKEHLVDMGITHGPAIQIEELFKELRKTAIEDSIQTSKMGKPSKNAPKDQT
SAMD9_Patr ....................................................................................................
SAMD9_Gogo .....................................G....................................Q...T.....................
SAMD9_Poab .....................................G..............................................M...............
SAMD9_Nole .................E...................G.........................................................V.....
SAMD9_Mamu ........Q...........R................G....................................Q.............R...........
SAMD9_Bota ..A.........................E....A...A...S..I..Y.T.D...A....F.......Q.....LE.SSG.PF..C.S..G..SV..T.-
SAMD9_Susc ..A.....K...........R............D...A.......N..Y.T....I.I...F.......H.....ME.ST..PS.IC.RE.G..DV..K.-
SAMD9_Eqca ..A.................R...............IA...S..I....T.NN.IE...........N.....Q..SS.G.L..N.KK.G...V..T..
SAMD9_Mylu ..A.............D...R..D.........K.............F.T.N..IE...........Q...K.Q.ESS..P...CQKK.G..KV..T..
SAMD9_Orcu ..E.PE..............R.......H....D..VA...S.....F...SD.IE.......V...G..R..Q..SSK.P.R..EQK.G...VSNK.P
SAMD9_Rano ..ET....K...........L.....R.........MA...S.V.....N.KN..E..................Q..PK.NLTK.C.KT.GR..I..T.-
SAMD9_Crgr ..EK.......N........L...G.......T.ML...........D.N...E...........A.....Q.SSSKQPT....RKRG...I..T.-
SAMD9_Capo ..E.PH....I.........R...............IA...S..T....N.SD......P.......VG..N..Q..SS..PV..P.AK.S.T.T.GK.H
SAMD9_Soar ..A.P..SK.P.........I...E.N..K.Y....VA.....D...L.T.K......V......L..N.V.K.Q..SS.N....REKKTNN..I.EV..

                   110       120       130       140       150       160       170       180       190       200
           ....|....|....|....|....|....|....|....|....|....|....|....|....|....|....|....|....|....|....|....|
SAMD9_Hosa VSQKERRETSKQKQKGKENPDMANPSAMS-----------TTAKGSKSLKVELI--EDKIDYTKERQPSIDLTCVSYPFDEFSNPYRYKLDFSLQPETGP
SAMD9_Patr ............R................-----------..............--...........L................................
SAMD9_Gogo .............................-----------..............--............................................
SAMD9_Poab .....H.........D..........TI.-----------.I..S........M--..Q.............N...................I.......
SAMD9_Nole ....A.....................T..-----------.I............--..E............................H............
SAMD9_Mamu .....S.........D..........T..-----------.I..V.E.......--......................................I.......
SAMD9_Bota --..-DGG...--..N.KKS.KV.D.TV.-----------.VT.......N.FM--..E..D..KK...TEP..MA.......D......N.K.......
SAMD9_Susc ---EKN.........D..TS.RVTD.TI.-----------.VTE..M..NN.FM--..E.ND.QKK.S.VE.M..P.......D......N.........
SAMD9_Eqca .M.E.NG........N..KS....APT..-----------.VT.D.....N.FK--VNE..D...K...VE............D......N.........
SAMD9_Mylu LM.E.NE......H....KS....A.T..-----------.V---.....S.F.--G.E..D...K.................D........C.......
SAMD9_Orcu LV...K.D.......N...S.P.DG..A.-----------PGE.EP..P.T..TDN..ERGD...KL.AKEPS.R.H..NK.DEQW....H.I.....--
SAMD9_Rano ---T.S....N.N.RAE..SCK.DT..V.PEGNSQRTAASPE......E.ND.V--...AVQ-QDK.A.PGP..IA...N..........H.I.......
SAMD9_Crgr ---T.I...P..N.R.E..T.K.DA..LR-----------PE...AE.A.N..M--...TEL-QGK.SPPGP..IA...N..........H.I.......
SAMD9_Capo LM...G......R.SD..ISHK.--------------------.E...PDS..M--.NQP.D------VKQP..LP...NT.........H.I.......
SAMD9_Soar L..S.NG.K..K...D..KS.TVDT.TTH-----------.V..S...VEN.F.--..D.---Q.K.L.TEQ..IP.......D.....EN.V.......

                   210       220       230       240       250       260       270       280       290       300
           ....|....|....|....|....|....|....|....|....|....|....|....|....|....|....|....|....|....|....|....|
SAMD9_Hosa GNLIDPIHEFKAFTNTATATEEDVKMKFSNEVFRFASACMNSRTNGTIHFGVKDKPHGKIVGIKVTNDTKEALINHFNLMINKYFEDHQVQQAKKCIREP
SAMD9_Patr ....................................................................................................
SAMD9_Gogo ..................................................................S.................................
SAMD9_Poab ....................................................I.............S.................................
SAMD9_Nole ..................................................................S.................................
SAMD9_Mamu ................E.............................................V...S.................................
SAMD9_Bota L...............E.G....A..................................T...VEF.TI......D......HQ........K..N.....
SAMD9_Susc L...............E...K.........................................VE..TV...F.D...M..HQ........K..N.....
SAMD9_Eqca L...............ER.R...I.......................................V..STV...T..D......QQ........K........
SAMD9_Mylu L..........E....KN............................................VN..SV......D......HQ........K........
SAMD9_Orcu ------------------------------------.............L......-.Q...V.LASI..N...D..DE..P....E.......S...V.
SAMD9_Rano L.....V.....L...E......I............................Q...Q.R...MEL.TV..D...D......VQ...V.............
SAMD9_Crgr L.....V.........EK.S...I.......I.........................Q.R...VDLSTV..DT.........DQ...A.............
SAMD9_Capo Q...........L.D......K.I.......T..............................M...SV......E...S..PQ...A.............
SAMD9_Soar L............K.IEKG.K..I.I.......K..................E.......I.VRFIAI...V..D...QI.HQ...EG...K..N.....
```

```
                   310       320       330       340       350       360       370       380       390       400
            ....|....|....|....|....|....|....|....|....|....|....|....|....|....|....|....|....|....|....|....|
SAMD9_Hosa  RFVEVLLPNSTLSDRFVIEVDIIPQFSECQYDYFQIKMQNY--NNKIWEQSKKFSLFVRDGTSSKDITKNKVDFRAFKADFKTLAESRKAAEEKFRAKTN
SAMD9_Patr  ..........................................--...T..................R...................................
SAMD9_Gogo  ..........................................--...T.....N..............................................
SAMD9_Poab  ..........................................--...T............E.......M.......T..T.....................
SAMD9_Nole  ...........A..............................--...T..............P.....M................................
SAMD9_Mamu  .....................................I...--...T........V...........M...A...T........................
SAMD9_Bota  .....................VV.KY...EV.........C--S.T..K..PN..V.....A.....M.SNM..K...L.L.R......E....C.V...
SAMD9_Susc  ..........IP.........VV.KY...EQE.........--S..T.K..P...V.M...A....VM..N.E.KT..L.L.K...A..E....C.L...
SAMD9_Eqca  ...........P.........V..KY...ED...R..I..C--D.NT.K..S...I.....A.....M..NA..KE..L.L.....A..E....Y.L..S
SAMD9_Mylu  ...........P.........V..KY...EH.........C--...V...R.H..V.....A.....M......KI.QLGLEA...H..E....C.I...
SAMD9_Orcu  ........D..V.........V..HY.V.GH......K.I.DN...K....S...VL....ARTVN.V.T.T..KM..LNL........E..DNCT....
SAMD9_Rano  ...........P.NK..........Y...KE.F.....HWH--KDET.Q.IP.Y.V.....PK....IG..A..K...L.L.A..D...E..K.SKV.SD
SAMD9_Crgr  ...........P.NK......VL..H...KE........HL--KSQT.Q..S.H.V.....PQ....IG..T..K...LGL.EV.D...E..T..KE.PE
SAMD9_Capo  ...........V.....V...VV..Y...KH...R.T....--.HET.QP.S.Y.V.I...PNT.N.I......E..QL.L.SV.T...E....CKL...
SAMD9_Soar  .......T.GIP...Y........KY...EH.........F--...T.NK.SR..V.....A.C..LM.TPA..K.Y.S.L........E...N..L..S

                   410       420       430       440       450       460       470       480       490       500
            ....|....|....|....|....|....|....|....|....|....|....|....|....|....|....|....|....|....|....|....|
SAMD9_Hosa  KKEREGPKLVKLLTGNQDLLDNSYYEQYILVTNKCHPDQTKHLDFLKEIKWFAVLEFDPESNINGVVKAYKESRVANLHFPSVYVEQKTTPNETISTLNL
SAMD9_Patr  ....................................................................................................
SAMD9_Gogo  ....................................................................................................
SAMD9_Poab  ......................................................................................E......M......
SAMD9_Nole  ...............................................................K......................E...S..M......
SAMD9_Mamu  ...................................................................QD..A..........I...G......K......
SAMD9_Bota  ...S.....................DW....I.....T.I.....................VSK........T..V......L...G...-T.K.TS...
SAMD9_Susc  ...S............K........DW....I.....T.I.....................VSE...........V......L...G...-..K.IS...
SAMD9_Eqca  ...N.....................DW..........N.I.....................VS..........IT......L.M.G...S..K.TS...
SAMD9_Mylu  N.NS.....IE.....K.........W..........N.I...............D.....VSK...N......IV...L..L...G......K..S...
SAMD9_Orcu  NRH-..FR..E...............DW..........N.IQY...................ES....RSF....L.......Q.S.G...T..M..N...
SAMD9_Rano  GSNSQ.Q..ID.....K......H..W..................................ES......F.G..........LFI.E.P.VS.K..S...
SAMD9_Crgr  GNKS..Q..ID...............W...I......N.NS..E.................ES......F.Q..........QF..E...L..K..S...
SAMD9_Capo  .NNS..S...T..............DW............II............I.......KTK..L..F....A....S..QF..E...TE.K..S...
SAMD9_Soar  ...S......RM.............SL.........QN.VN.........L..........ASK..................Q...E...TD.K..S...

                   510       520       530       540       550       560       570       580       590       600
            ....|....|....|....|....|....|....|....|....|....|....|....|....|....|....|....|....|....|....|....|
SAMD9_Hosa  YHQPSWIFCNGRLDLDSEKYKPFDPSSWQRERASDVRKLISFLTHEDIMPRGKFLVVFLLLSSVDDPRDPLIETFCAFYQDLKGMENILCICVHPHIFQG
SAMD9_Patr  ....................................................................................................
SAMD9_Gogo  ....................................................................................................
SAMD9_Poab  ...............N............................R.......................................................
SAMD9_Nole  ......................L.......................................................................Q......
SAMD9_Mamu  ............S.......................................................................I........Q......
SAMD9_Bota  .Q.L..............E...L.A......K..E.........Q.................................................DSR.C..
SAMD9_Susc  .Q.L..................L........K..E.......I..............................................M...A.SR.C..
SAMD9_Eqca  .Q..........I.........L........K..E...........................................................S..C.R
SAMD9_Mylu  .Q....................L......K.K..E............V....................................I.....V...S..C.R
SAMD9_Orcu  .Q..........S..GI.....L..I........E............V..K...........P................................Q.C..
SAMD9_Rano  .Q.............E.D..Q.L..I........E..R......RD..................E.................N..............Y.V
SAMD9_Crgr  .Q..........S..E.D....L..I........E..R....F.RD....K.R..........P.............G..............S.FSS.Y.V
SAMD9_Capo  .QK.........S.......E.V..V...K....E.........R......................K........T.....S..........Q.N.C..
SAMD9_Soar  HQ.....-.........D....L..????????????????????????...--...-....P....-..-Y............L....V.-LNSQLC.I
```

```
                    610       620       630       640       650       660       670       680       690       700
           ....|....|....|....|....|....|....|....|....|....|....|....|....|....|....|....|....|....|....|....|
SAMD9_Hosa WKDLLEARLIKHQDEISSQCISALSLEEINGTILKLKSVTQSSKRLLPSIGLSTVLLKKEEDIMTALEIICENECEGTLLEKDKNKFLEFKASKEEDFYR
SAMD9_Patr ....................................................................................................
SAMD9_Gogo ....................................................................................................
SAMD9_Poab ....................................................................................................
SAMD9_Nole ....................................................................................................
SAMD9_Mamu .................................................................S..................................
SAMD9_Bota .........TTQ...L.N..V.S......................F.....S.................L......D.I.....K.L.....L.......
SAMD9_Susc .........A.QE..L.............................F...V.S......R..........L.............EK.......L.......
SAMD9_Eqca .........T.Q...L.N........................L..F.....S.............S...L..............K...D..T........
SAMD9_Mylu .........T.-...L......S.................K..Q.F.....S.....EM..........L.....K.....N..........L.......
SAMD9_Orcu .........T.E...L.N......N.Q..................F.....S.......Q..T......L......D...KD..K.........KA....
SAMD9_Rano ......V..SSQ...L...S.FS.N.A...........L......F...V.S......R..E.......L.............RE.LF...T...K....
SAMD9_Crgr .........AGQ...L.NRS....N.A...........L......F.....ST........-.......L...........N..K.LF...TA..Q....
SAMD9_Capo .........TNE...L.DK.....N..Q......N...I....E......CS..........-......L.V........H...T.LQ...T........
SAMD9_Soar .........KIQ...LAN......N.................V.K......S.................L......D.D.........A..TL.......

                    710       720       730       740       750       760       770       780       790       800
           ....|....|....|....|....|....|....|....|....|....|....|....|....|....|....|....|....|....|....|....|
SAMD9_Hosa GGKVSWWNFYFSSESYSSPFVKRDKYERLEAMIQNCADSSKPTSTKIIHLYHHPGCGGTTLAMHILWELRKKFRCAVLKNKTVDFSEIGEQVTSLITYGA
SAMD9_Patr ....................................................................................................
SAMD9_Gogo ..................................................................................A.................
SAMD9_Poab ....................................................................................................
SAMD9_Nole ..................................................................................A.................
SAMD9_Mamu ...........................................C.......................D.........................N......
SAMD9_Bota ....T.........N............K..E...RG.....S.CV................................................N.....T
SAMD9_Susc ..............N........ER..K..T...E..A.....CV...................V................M...C.......N.....T
SAMD9_Eqca .............K...L.........I..E....W.......CV.....................................M.........AT......
SAMD9_Mylu ..............N............K..R..H.......S.CS............................................K...N.....T
SAMD9_Orcu ..............NH..A................W......ICA................................................N.....T
SAMD9_Rano ..............N...S.......VK..KK..EW....Q.VCA.....................................E..........N......
SAMD9_Crgr ..............N...S........K..KK..EW....Q.VCA......................D..............E..........N......
SAMD9_Capo ...........-..Y...S........K.KE....Y..P..LMCV..............................................V........
SAMD9_Soar .............KN...A........K.KD....W-....S.CV...Q..................N.....................K.I.D.....T

                    810       820       830       840       850       860       870       880       890       900
           ....|....|....|....|....|....|....|....|....|....|....|....|....|....|....|....|....|....|....|....|
SAMD9_Hosa MNRQEYVPVLLLVDDFEEQDNVYLLQYSIQTAIAKKYIRYEKPLVIILNCMRSQNPEKSAR-IPDSIAVIQQLSPKEQRAFELKLKEIKEQHKNFEDFYS
SAMD9_Patr ........................................................K...K-......L...............................
SAMD9_Gogo .............................................................-......................................
SAMD9_Poab .....................................V.......................-......................................
SAMD9_Nole ......I...................S.............................K....-......................................
SAMD9_Mamu ....D........................................................-......................................
SAMD9_Bota T.N...L.I.................A..H..V.NR..............R.........K-.S....L...................ED.....Q....
SAMD9_Susc ASS...L................F..A....T..N...............R.........K-N.N...L..N..S...........K.E......K....
SAMD9_Eqca T.Q...L...........E.......S.....V.N.......................C.K-NL.G..L..N..........F.....E...E..K....
SAMD9_Mylu A.H...L............GDI....A......TS...............V.......C.K-MS....L.....S.............E...........
SAMD9_Orcu T.H...L...............F...S.....V.E.H.......................K-....V.LLHH.............EK..KEYD.......
SAMD9_Rano T.H...L...................T.....V...H.............T.........K-N...V.LV....D..........T........V.....
SAMD9_Crgr TS....L...................A.....V.N.H.............T.........K-....V.LVH...D..............KH.E.......
SAMD9_Capo T.H...L...................AA....V.Q...............A.........K-......LR.K..R..KE...........H.........
SAMD9_Soar TSH...L............E......S..H..VSS.H.................D.R.C.KNF.N...L......S............EK....V.....
```

```
                  910       920       930       940       950       960       970       980       990      1000
           ....|....|....|....|....|....|....|....|....|....|....|....|....|....|....|....|....|....|....|....|
SAMD9_Hosa FMIMKTNFNKEYIENVVRNILKGQNIFTKEAKLFSFLALLNSYVPDTTISLSQCEKFLGIGNKKAFWGTEKFEDKMGTYSTILIKTEVIECGNYCGVRII
SAMD9_Patr ....................................................................................................
SAMD9_Gogo ....................................................................................................
SAMD9_Poab .....................................T..............................................................
SAMD9_Nole ............M.......................................................................................
SAMD9_Mamu .......................................................R............................................
SAMD9_Bota ..........K...D.......E...S.................................TS..........................V...........
SAMD9_Susc ........D.K...............C.................................T..........L............................
SAMD9_Eqca .........EK...............S............................N....T.......V...............R...V...K.......
SAMD9_Mylu ........D.K...............S.................................TT...Y.....L................V...K.......
SAMD9_Orcu ........................DPS............................N....A..........L.E..........R...L...M.......
SAMD9_Rano .........................VT.......A......AF............L....S....Y...................S......S.......
SAMD9_Crgr ........DRK..............VA.......A......T.............YY...S........................S......S.W.....
SAMD9_Capo .............KK..........KS.................SG..........Y...A..........L............Q.K.E...T.......
SAMD9_Soar ........D.M......K........C..................E.........Q....T......EL..L..............D.V...C.......

                 1010      1020      1030      1040      1050      1060      1070      1080      1090      1100
           ....|....|....|....|....|....|....|....|....|....|....|....|....|....|....|....|....|....|....|....|
SAMD9_Hosa HSLIAEFSLEELKKSYHLNKSQIMLDMLTENLFFDTGMGKSKFLQDMHTLLLTRHRDEHEGETGNWFSPFIEALHKDEGNEAVEAVLLESIHRFNPNAFI
SAMD9_Patr ....................................................................................................
SAMD9_Gogo .........................H..........................................................................
SAMD9_Poab .....K......................................................S............................G.R........
SAMD9_Nole .....K.....................................................D.............................G....K.....
SAMD9_Mamu .....K..................................................N................................G..........
SAMD9_Bota .P...IR......I..D.D....T.........Y...I.R....E.VQ......Q.N.N...M.TL..........E...V..KN..RGG.R........
SAMD9_Susc .P...TR......I..N.D....I.........Y...I..D...E..Q......Q.ND......T..........R....V..KN....G.RQ..S....
SAMD9_Eqca .P...DR.....RT....D..............YE..L...R.SEHIQ......Q.N.......TL..............V..KE....G.R...S....
SAMD9_Mylu .P...IR......I..D.D.....W......V.Y...I.R...F...Q......Q.I.......T..........E....K...K....G..........
SAMD9_Orcu .P...SL......N..G.GRC.M..........YE..I........VQ........N...D...................D..KM..EKG.N...K....
SAMD9_Rano .H...TL......R..N.S..K.VM........Y...I.....Y...Q...I..Q.N...R...................D..K...H.ATR..D.....
SAMD9_Crgr .HE..SA......R..N.S..E.VMN.......Y.M.I.....F...Q......Q.N.......................D..KD....ATR........
SAMD9_Capo .P...IL.........D.D..............Y...L.R...F...Q......E.S.Q.....T..................KQ..CQG.E..K.....
SAMD9_Soar .P...LR......L..Q...........R....Y.R....T..I..IQ....I.Q.N......ET..................KE..I.GVD..KQ....

                 1110      1120      1130      1140      1150      1160      1170      1180      1190      1200
           ....|....|....|....|....|....|....|....|....|....|....|....|....|....|....|....|....|....|....|....|
SAMD9_Hosa CQALARHFYIKKKDFGNALNWAKQAKIIEPDNSYISDTLGQVYKSKIRWWIEENGGNGNISVDDLIALLDLAEHASSAFKESQQQSEDREYEVKERLYPK
SAMD9_Patr ....................................................................................................
SAMD9_Gogo .........................................................RS..........N..............................
SAMD9_Poab ...............D.......E.................................R...........N............................L.
SAMD9_Nole ..............................................M.......ER.............N..............................
SAMD9_Mamu .......................................................R.R....N......N...Q.............Y..........Q.
SAMD9_Bota ....T......E...DS..H..N...K...............F.......MDD.ER.RS...GE.SD.....VQ..N...............G...F.Q.
SAMD9_Susc ....S......ER..NS..H..NE..K.........................-D.R.W...A...AD.....V...N......R............F.Q.
SAMD9_Eqca .........L.E...SS..K..N...K...H.....................D..K.R.....N.TD..E..VE.TN............Q..G...F.Q.
SAMD9_Mylu ...........ER..TS..H..N...N...N...................T.N.ER.R....A..TE.....V...E..........YK.D.AM..FNQ.
SAMD9_Orcu R.......---------------------------...........L.....D..RGNT..AN..TD..Q..V...AE.....R........G.....Q.
SAMD9_Rano .........L.E...ES..L.....RK.A.N...............L...V.D.IK..I....E.TE.....K...E...D....T......G..SV.Q.
SAMD9_Crgr .........L.E...K...F.....RT...N....................DD.IR..S..AE..TD...S.V...D...D..R.T......G....NQ.
SAMD9_Capo ..V....Y..RE...N...L...E..K...N.....................DTER.RD......ST.........N......R........G....GQ.
SAMD9_Soar ...........E...ER..E..NK..N...Y....................DD..R.R....A..TM..E..V...N......R...YK.R.A.GKFHQ.
```

```
                   1210      1220      1230      1240      1250      1260      1270      1280      1290      1300
            ....|....|....|....|....|....|....|....|....|....|....|....|....|....|....|....|....|....|....|....|
SAMD9_Hosa  SKRRYDTYNIAGYQGEIEVGLYTIQILQLIPFFDNKNELSKRYMVNFVSGSSDIPGDPNNEYKLALKNYIPYLTKLKFSLKKSFDFFDEYFVLLKPRNNI
SAMD9_Patr  ....................................................................................................
SAMD9_Gogo  ..........................................................D.........................................
SAMD9_Poab  ..........................................D....I....................................................
SAMD9_Nole  ..................................K.......D....I....................................................
SAMD9_Mamu  ..........................................D........G.....Q.D..Q.....................................
SAMD9_Bota  .........T...L..............F.............D.I..I...K....EA...F..V...F.S...N.........................
SAMD9_Susc  ..........................................D.I..I..TT.........F..V.N.F...................D...........
SAMD9_Eqca  ..................A...............S.......D.I..I.........SS..F......F.....N..C...RC.....D...........
SAMD9_Mylu  ......................A...................D.I..I..N..........F......F.......RS..........D........S..
SAMD9_Orcu  .........................................KD.......T......S...F.SV...F.....N.R...........D...........
SAMD9_Rano  ...........................K.L...NS.....RQV.I..I..NC.....L-..F.SV...F.S...N.QS..........D...........
SAMD9_Crgr  ........................K...........D.....D.I..I..NR.....L-..F.I....F.....N.QS...RA.....D...........
SAMD9_Capo  ..........S..H........A...........K.....R.D.I..I..N......L-..F......F.....T..S...DA.....D...........
SAMD9_Soar  ..................................S....F.KD.I..I..T......S...F...S.F.S...N.R....N......D..........M

                   1310      1320      1330      1340      1350      1360      1370      1380      1390      1400
            ....|....|....|....|....|....|....|....|....|....|....|....|....|....|....|....|....|....|....|....|
SAMD9_Hosa  KQNEEAKTRRKVAGYFKKYVDIFCLLEESQNNTGLGSKFSEPLQVERCRRNLVALKADKFSGLLEYLIKSQEDAISTMKCIVNEYTFLLEQCTVKIQSKE
SAMD9_Patr  ....................................................................................................
SAMD9_Gogo  .................................D.R..............S...........................EY..........R.........
SAMD9_Poab  ..............................K-.DP...........................................E...K.......R.A.......
SAMD9_Nole  ...................I....VS.....-KD...............SS....................A..V...EN..DK....S...........
SAMD9_Mamu  .............V.....T......G...D-I...........I....KS...........................ED...K.A..........E...
SAMD9_Bota  ...................A...GPS..L..-KDF...L.V.....LY..S.EV...............A....VH..ED.MDK....F.......LI..
SAMD9_Susc  .........K...V.....A...GPS.D..S-KY....L.L.....Q...S.EV...............N.K..A...ED.M.K.S..F.....RML...
SAMD9_Eqca  ......R.......H........GPSV....-KD..A.L.I.....LY.KS.EV...........N...N.....R..DD...K....F..G.....P..
SAMD9_Mylu  ....QS.............A...GPF..L..-RD...QL.L..R..LY..S.EV..................I..N..ED..KK....F...A.R..Q..
SAMD9_Orcu  .....T.......TC..R.L...GPSA.ILS-K.....I.LS...QE...S.E..............V....H.....EN..HK....F..S.E...L..
SAMD9_Rano  E.Y....S.....M......E..GP.A.L..-QNFR..L.L......T..S.E.................PK.TV.S.ED...K........AI...Q..
SAMD9_Crgr  ..Y..S.C.S..V....R.....S.......-QNFR..LTL......I..T.E.................PK..V.S.EG...Q..S.....VI.T.Q..
SAMD9_Capo  ..H.........VV.....I...GPSG..L.-RD.EL.L.L......NQ.S.E................T.-..V.S.EY...T.I..FD..I.RMHL..
SAMD9_Soar  ..SD--.............G...D.FNGT.S-KDF..NI.LS.KI.LF.SK.EV.............V.N.NSP.E..ES..EQ.S.I..H.AAS--T..

                   1410      1420      1430      1440      1450      1460      1470      1480      1490      1500
            ....|....|....|....|....|....|....|....|....|....|....|....|....|....|....|....|....|....|....|....|
SAMD9_Hosa  KLNFILANIILSCIQPTSRLVKPVEKLKDQLREVLQPIGLTYQFSEPYFLASLLFWPENQQLDQHSEQMKEYAQALKNSFKGQYKHMHRTKQPIAYFFLG
SAMD9_Patr  ..............K.....................................................................................
SAMD9_Gogo  ..............K...........................................Y.........................................
SAMD9_Poab  ..............K...........................R.......................K.....S...E.......................
SAMD9_Nole  ..............K...........................R.......................G.........E.......................
SAMD9_Mamu  ..............K...T......................HR.......................Q...A.....E.......................
SAMD9_Bota  .Q.........Y..K...KI...IK........L..QT.MS.RYP...................D.RL.EK..SS.E...R......Y............
SAMD9_Susc  .Q.........Y..K...KI.M.IK...E.......QAETICRQ....................D.K..EK..RS.E...R......C............
SAMD9_Eqca  .Q.........Y..K...KI...IR...........Q.....R.................H...D.K..EK..RS.E...R......Y............
SAMD9_Mylu  .Q.........Y..K...KI.M.TK........I..Q..PS...................K...D.K..EKH.RS.Q...R......Y............
SAMD9_Orcu  .Q............K...KS..TIK......Q.I..QV..NCR.....................D.K..EK...S.E...R......Y............
SAMD9_Rano  .Q.....S......N.I.KF...IK...E....I..............................D.K..EK.....EK..R....RLY............
SAMD9_Crgr  .Q............N.A.K.....K...E....I..............................D.K..EK..W..ET..R....R.Y...........E
SAMD9_Capo  .Q............K.A.K....IK...EH..DI..QV.P.HR.....................D.KE.EK.V.S.EK......................
SAMD9_Soar  .Q.......V.Y..K...KF.S..K...E.......-V.TN.R.P.............T.N...D.K..EK..RL.ET..L.K.R..Y............
```

```
                   1510      1520      1530      1540      1550      1560      1570      1580      1590      1600
            ....|....|....|....|....|....|....|....|....|....|....|....|....|....|....|....|....|....|....|....|
SAMD9_Hosa  KGKRLERLVHKGKIDQCFKKTP-DINSLWQSGDVWKEEKVQELLLRLQGRAENN-CLYIEYGINEKITIPITPAFLGQLRSGRSIEKVSFYLGFSIGGPL
SAMD9_Patr  ..................E...-................................-.............................................
SAMD9_Gogo  ......................-................................-.............................................
SAMD9_Poab  ..E..K............E...-................................-.............................................
SAMD9_Nole  .....K............E..L-................................-.............................................
SAMD9_Mamu  .....K............E...-..............A.................-..................T..........................
SAMD9_Bota  ..NSVN............E..A-...F..........K...K.....K..VG..-..H..........................................
SAMD9_Susc  ..NNMN..I.R......C...AA..K.F.........KE..K..F..K......-..............................D.........T....
SAMD9_Eqca  ..NNMT............RN.S-..............RN........K......-.......V.D..........F........................
SAMD9_Mylu  ..NNMN...........YG..L-...IW....E....K.........K......-..........R.........W........................
SAMD9_Orcu  ..NNMN............E..L-..............R................-.......VSD...L......W.........D..............
SAMD9_Rano  ..NSRN..I......EN.ENLS-....W.HT.K..E.K...D..V..P......-................K.THF.......................L.
SAMD9_Crgr  ..NNRN..I......EN.E.VS-....W.H..K..E.K...D............-N.............Q...TH........................L.
SAMD9_Capo  ..TNMN.........E..GEMS-...F.....K..E.K...D............EK.F.................F........................
SAMD9_Soar  .A.NIN............G..--.......N....R.K...D.....N......-.V..D...K...I.....T.F........................

            ....|..
SAMD9_Hosa  AYDIEIV
SAMD9_Patr  .......
SAMD9_Gogo  .......
SAMD9_Poab  .......
SAMD9_Nole  .......
SAMD9_Mamu  .......
SAMD9_Bota  ......I
SAMD9_Susc  .....VI
SAMD9_Eqca  ......I
SAMD9_Mylu  ......I
SAMD9_Orcu  ......I
SAMD9_Rano  ....K..
SAMD9_Crgr  ....K..
SAMD9_Capo  ....Q.L
SAMD9_Soar  ....QVI
```
