# Supplementary material for: Evolution and divergence of the mammalian SAMD9/SAMD9L gene family
Source: BMC Evol Biol. 2013 Jun 12;13:121. doi: 10.1186/1471-2148-13-121 (PMC3685527; doi:10.1186/1471-2148-13-121)
Supplement: Additional file 6: Figure S5 — Mammalian SAMD9L deduced protein sequences alignment. SAMD9L deduced protein sequences from eighteen species were aligned with ClustalW implemented in BioEdit. The abbreviations correspond to the following species common names: Hosa - Human; Patr - Common chimpanzee; Gogo - Western gorilla; Poab - Sumatran orangutan; Nole - Northern white-cheeked gibbon; Caja - Common marmoset; Mamu - Rhesus monkey; Loaf - African bush elephant; Eqca - Horse; Calu - Domestic dog; Aime - Giant panda; Ereu - West European hedgehog; Orcu - European rabbit; Mumu - House mouse; Crgr - Chinese hamster; Rano - Brown rat; Capo - Domestic Guinea pig; Soar - Common shrew. To access the species scientific names, the list of abbreviations should be consulted. Codons are numbered according to human SAMD9L protein. “?” represents undetermined codons; “.” represents identity with the reference sequence of human SAMD9L protein. [file 1471-2148-13-121-S6.pdf]

```
                     10        20        30        40        50        60        70        80        90       100
             ....|....|....|....|....|....|....|....|....|....|....|....|....|....|....|....|....|....|....|....|
SAMD9L_Hosa  MSKQVSLPEMIKDWTKEHVKKWVNEDLKINEQYGQILLSEEVTGLVLQELTEKDLVEMGLPWGPALLIKRSYNKLNSKSPESDNHDPGQLDNSKPSKTEH
SAMD9L_Patr  .......................T...............G...............I............................................
SAMD9L_Gogo  .............................S.......F.................I............................................
SAMD9L_Poab  ...........E...........T................................I.....R............FY........................
SAMD9L_Nole  .......................T................................I.....R......................R...............
SAMD9L_Caja  .....T.................T.....D.K......N.................I.....R..............NT.A..........SH..S.....
SAMD9L_Mamu  .........I.............TD...............................I.....R........A....................H.....R..
SAMD9L_Loaf  ..E..T....TQ...........T.....D.K......N.................TD....R........G.....NS....H.Q.FE...HT.T..K.Q
SAMD9L_Eqca  .NE.AN....T.........Q..TK....G.K......N.................I.....R...I....A..R..NS....N.Q.S....HT.S..E..
SAMD9L_Calu  .NEE.N...VVD........Q..TKH.NVD.K......R.................R.......S......K.....NS.S..N.Q.S....HT....K..
SAMD9L_Aime  ..E..N...I.N........Q..TK....D.K......T.............N..R.....R........A..R..NS.S..N.Q.S..V.HT....K..
SAMD9L_Ereu  .DE..N....TQ........Q..TN..Q.D.K......C.....R...V......I.....R........KFKS..NL.SKNS.QNS....HAE...K..
SAMD9L_Orcu  .NE..T...LV............T....VD.K......N........K....D..K.....R........AC...LNS......Q.S.K...I....KKQ
SAMD9L_Mumu  ..G..TQ.KL.........R...T...N.V.K.A...FK.....M.......E..R.....R........M....I-.....H.Q.SRE.NDK.L.TK.Q
SAMD9L_Crgr  .NE..TA.KLV......Q....IT...N.D.K.AE..FN.....M..........R.....R........A....SN-AT....Q.SK..H.K.L.IK..
SAMD9L_Rano  .DRH.TQ.KL.........R..IT.....D.K.A..VFE.....M..........R.....R........M....I-.....GH.Q.SR..N.KTL.IN.Q
SAMD9L_Capo  .NE.GT..........D.....IT.E...D.K.....FN.................K.....R........M.....TSF.....PNSR.VHDT.S..K..
SAMD9L_Soar  ..EHTN....T.A....D..Q..T.V.Q.D.E......N.K.S..A...I..E..R.....R........T.....NI....NSPNSRP..STNV..KKS

                    110       120       130       140       150       160       170       180       190       200
             ....|....|....|....|....|....|....|....|....|....|....|....|....|....|....|....|....|....|....|....|
SAMD9L_Hosa  QKNPKHTKKEEENSMSSNIDYDPREIRDIKQEESILMKENVLDEVANAKHKKKGKLKPEQLTCMPYPFDQFHDSHRYIEHYTLQ-PETGALNLIDPIHEF
SAMD9L_Patr  .................................................D..................................-...............
SAMD9L_Gogo  ..............T..................................D..................................-...............
SAMD9L_Poab  .....Q........................EQ.................D..................................-...............
SAMD9L_Nole  .....Q........K.............V..Q.................D..................................-...............
SAMD9L_Caja  H.K..Q...K.K.....S.......V....EQK...........AE...D...D.....R.............GQ.........-....S..........
SAMD9L_Mamu  ..D..Q........T..........V....ER..........E......D....E.............................-...............
SAMD9L_Loaf  -.KA.-----K.K.I..S..H.LT..G...ER........ATN...AT.D...N.V.T................Q..T...I..-....P..........
SAMD9L_Eqca  KEK.QQ....K.K.T.....HNL..T..T.EQ........A.N..V-T.D.Q.N..QA................Q......I..-....P..........
SAMD9L_Calu  P.K.QQM.....K.VL....H.L..A..T.EQ.......DA.N.G.T.EDQNED..GIK.............N..C...NSV..-....P..........
SAMD9L_Aime  P.K.QKM.....K.VL..N.H.L..M..T.EQ....V..DA.N.EVTTEDQNEDRTET..............N.Q.....SI..-....P..........
SAMD9L_Ereu  .RK--.---KT..T-L..N.H.V...QNA.AQ.LAATGK.AQ...GITEE.-.K.R.IV.S...........A..H.T...I..-....P..........
SAMD9L_Orcu  ..K..QKS...GT...L...H.L..TTE.EVQ...PL..KA...TV..AD.-ENAIQT.R..............Q......I..-....P..........
SAMD9L_Mumu  .TK---..N.....V...S.HGL..TGQNEEQ.PS.T...M.GD.V-T.DMEDN.P....MS.T.....S.C.VKQ....SI.RVA...P..........
SAMD9L_Crgr  P.K---.NN...KLI...SGH.L..MGLNTEQ.PS.L..KA.SD.L-T.DMEGNTA....MS.......S...DR....R.I..VA...P......V...
SAMD9L_Rano  P.K---SNS.....I...S.FGL..TGQNEEQ.PSI..V.T.GD.E-T.DM.DNMP.S..MS...H..NFA..AK.....SI.RVA...P......V...
SAMD9L_Capo  ..K.----NL.KEL.P.S..Q.LS.S.N..DQD..P.E..AAN..S.TID...N...T.N...P.....N...GQ......I..-....P..........
SAMD9L_Soar  K..SQ--NTK..KLV...S.H.LK.SVNT.EQ..VPIEKDS..YQGIPEDQ-.....K..S..V..........R..V..QI..-....PR.........
```

```
                     210       220       230       240       250       260       270       280       290       300
             ....|....|....|....|....|....|....|....|....|....|....|....|....|....|....|....|....|....|....|....|
SAMD9L_Hosa  KALTNTETATEVDIKMKFSNEVFRFASACMNSRTNGTIHFGVKDKPHGEIVGVKITSKAAFIDHFNVMIKKYFEESEINEAKKCIREPRFVEVLLQNNTP
SAMD9L_Patr  ....................................................................................................
SAMD9L_Gogo  ....................................................................................................
SAMD9L_Poab  ...........A..N.........................................N..S.........R..............................
SAMD9L_Nole  ...........A................................N.......................................................
SAMD9L_Caja  .......R...E..........L..............................N....DV......L.........D.......................
SAMD9L_Mamu  ...........A..............................................D.......L............Q..................M.
SAMD9L_Loaf  ......D....K..H.......................................VA..DD..N...L...Q...D.D.K..E....Q.......Q.....
SAMD9L_Eqca  ...........K.........A.....................N..........V...DS..........Q.......KV....................
SAMD9L_Calu  .......A...K...............................NR...Q.....VAN.D.......I..RQ.........V..................L
SAMD9L_Aime  .......A...K...............................N....Q.....V...D..........RQ...GN........................
SAMD9L_Ereu  ...........K..............................L...........VR..D.......L...Q.......K..................Y..
SAMD9L_Orcu  ......AA...E...........................................S..DVL.N...........D.D....................SM.
SAMD9L_Mumu  ..F...KK...E.........T....A..........................QV...DI.VN...T..T....D...S..RA................Q
SAMD9L_Crgr  ...........E.........I..........H......................P..DV.V....K.......D.D.K..RE.................
SAMD9L_Rano  ..F.......QEQM.......T....A....T.....................QV...DI..N......T....D.D.H..RA............L...Q
SAMD9L_Capo  .......N...E.L.......T................................V...D.......L...........S..Q....Q..ID........L
SAMD9L_Soar  .......SVE.K..............................L...Q.......V.R.DS......K...H...D...K..R.................S
```

```
                     310       320       330       340       350       360       370       380       390       400
             ....|....|....|....|....|....|....|....|....|....|....|....|....|....|....|....|....|....|....|....|
SAMD9L_Hosa  SDRFVIEVDTIPKHSICNDKYFYIQMQICKDKIWKQNQNLSLFVREGASSRDILANSKQRDVDFKAFLQNLKSLVASRKEAEEEYGMKAMKKESEGLKLV
SAMD9L_Patr  ....................................................................................................
SAMD9L_Gogo  ...........................................I........................................................
SAMD9L_Poab  .........I.......K..............T........................................................I..........
SAMD9L_Nole  .........IN......K.....................T............................................................
SAMD9L_Caja  .......I.I.....V.K.M..C.R..SYT..K....K.S...............DPNK............E.............R...K..........
SAMD9L_Mamu  .........I...Y...K..........F..........S................................................T...........
SAMD9L_Loaf  ..K......V..R..V.K......N......GS.R.DKDY.....Y....K.....VR...A.....TL.FE.V............V..N.........A
SAMD9L_Eqca  .........V.......KE.....K..N..NEK....EDH.....D....K.....G....T.....T.......T.........EV..N......Q...
SAMD9L_Calu  .........V.....V.EK...L.R..N..SET..P..D......D.P..K.....V...ELNL.......T.V...........E...DR.....Q...
SAMD9L_Aime  .........V...Y.V.GE...L.R..S..NET..P..D......D.P..K.....V....IAY.E.S..F..VAT...V.....EV..N......Q...
SAMD9L_Ereu  .........V.......KE....TN..SY.NDT.....ES.....D....V.....G....KN..T........AED..D..K.HE...N...C..Q..I
SAMD9L_Orcu  .........V.....V.KE.....K..S...NV.....EY...I............A....P.....T..................R..T..........
SAMD9L_Mumu  .N.......V..R....QE.....M..SSTG.T...SKDT..........KN..G.PN...RE..K..ED..MWT....A....LR.-VT.........S
SAMD9L_Crgr  .........V.......KE.....I..SYT..T...SKENC......T..KN....A....RE.RK..E...AWT........QQR.-VT.........A
SAMD9L_Rano  .N.......V.......QE....VML.T.TGTT...SKDT..............G.P....RE..K..E...MSI....A.-..CMV-VS..D......S
SAMD9L_Capo  .........I...Y.V.KE.....KL.SLT..T............D....K.....V.R.EIE....YE....CA.A..D....HQ..TT.R........
SAMD9L_Soar  .........V.......QE....TK..EL..G.....TE.T........TK.....ERT..AG......D....ITM......KDEV.VN.Q....Q..I
```

```
                     410       420       430       440       450       460       470       480       490       500
             ....|....|....|....|....|....|....|....|....|....|....|....|....|....|....|....|....|....|....|....|
SAMD9L_Hosa  KLLIGNRDSLDNSYYDWYILVTNKCHPNQIKHLDFLKEIKWFAVLEFDPESMINGVVKAYKESRVANLHFPNQYEDKTTNMWEKISTLNLYQQPSWIFCN
SAMD9L_Patr  ......................................M..........................................R..................
SAMD9L_Gogo  .................................................................................R..................
SAMD9L_Poab  .................................................................................R..................
SAMD9L_Nole  ..................V..........................................Q...................R..................
SAMD9L_Caja  ...T.................I.............................QS.......R.................M..R....A.............
SAMD9L_Mamu  ...................................................V.............................R....A.............
SAMD9L_Loaf  .....S.....D............S....T.....................VSK.................D...K.AATIR.M.F.....E........
SAMD9L_Eqca  ...............N.....I.......T.N...................QSK..A..F.K.........S...E.S.P.R....S.............
SAMD9L_Calu  ...............N.............T.N......M.L..........VSK.......K..I........F.E..N.IR....S......T......
SAMD9L_Aime  ...............S.............T.N......M............VSK.......K...........F.E.SN.IR....S....E........
SAMD9L_Ereu  ......Q........N.........N.S.R--...................ESK..A.T.R........-....AE...T.R....SXS.W---------
SAMD9L_Orcu  ...............S.............T.....................G...................S......SSLR..............V...
SAMD9L_Mumu  ...TRHQG...E...........T.A.T.LE..E.I..M.L....D...Y.H.K......R...I....L.SH..E.-.TIA......K..E........
SAMD9L_Crgr  ...TRHQG...............N.A....E.........L....D...Y.Q.K......R...I....L.S...E.-STIE......K.FE........
SAMD9L_Rano  ...TRHQG...K...........S.V.T.ME....I....L....D...Y.H.K......R........L.SH..E.-.TIE......K..E........
SAMD9L_Capo  ...............NS............MQ....................R....A...QK.....M.......E.K.T.RQ...S....E.T......
SAMD9L_Soar  ...........S...N.............T.Y.......S.....D.....VSK....VFQ..........S..VEGKSTQN.TVCS......T......
```

```
                     510       520       530       540       550       560       570       580       590       600
             ....|....|....|....|....|....|....|....|....|....|....|....|....|....|....|....|....|....|....|....|
SAMD9L_Hosa  GRSDLKSETYKPLEPHLWQRERASEVRKLILFLTDENIMTRGKFLVVFLLLSSVESPGDPLIETFWAFYQALKGMENMLCISVNSHIYQRWKDLLQTRMK
SAMD9L_Patr  ....................................................................................................
SAMD9L_Gogo  ..................H.................................................................................
SAMD9L_Poab  .........H..........................................................................................
SAMD9L_Nole  ...............Y....................................................................................
SAMD9L_Caja  ..............A..........................R.........FT....V..........................S...............N
SAMD9L_Mamu  ....................................................................................................
SAMD9L_Loaf  .....NN......D..........................K.........F..............C...........I...C.K.Q.........R..LT
SAMD9L_Eqca  ..T...D.RH...............I....Q......V....R.........A..........S.C...............C...Q.............LT
SAMD9L_Calu  ......N.S.........H.................................P.............C...........I...C.................LT
SAMD9L_Aime  ......N.S.........H...............................................C...........I...C...Q.............LT
SAMD9L_Ereu  -------------..RQ..E....GRA..NY.....................P..........P.CT.FKV.N..Q.IM..C.TPQ.............FV
SAMD9L_Orcu  .....T......F.................F........A..........................C...............C...Q.........K..LT
SAMD9L_Mumu  ..V..---SCQ.........D...G..R..S.......IVK..V........PI.NQK.......C....VFN..D.....C...A...Q.S....V.LE
SAMD9L_Crgr  ..L...NDSCQ.........D...G..R..S........V...V........P.NNQK.......C....VFN..D.....C..P......S....V.LE
SAMD9L_Rano  ..V..---SCQ.........D...G.....S....G..IA...V........P..NQK...L...C....VFN..D.....C..PS...Q.S....V.LE
SAMD9L_Capo  .....QN.......A.....D..C......S......L.AK...............L...FT...C......R.........C...K.............LT
SAMD9L_Soar  ......GAV.........NKN.....T.........S..A............P............CV..K..N....I...C..LD..KQ.....KA.LI
```

```
                     610       620       630       640       650       660       670       680       690       700
             ....|....|....|....|....|....|....|....|....|....|....|....|....|....|....|....|....|....|....|....|
SAMD9L_Hosa  MEDELTNHSISTLNIELVNSTILKLKSVTRSSRRFLPARGSSSVILEKKKEDVLTALEILCENECTETDIEKDKSKFLEFKKSKEEHFYRGGKVSWWNFY
SAMD9L_Patr  ....................................................................................................
SAMD9L_Gogo  ....................................................................................................
SAMD9L_Poab  .............................Q........H..........................KD......E....................C.....
SAMD9L_Nole  .............................Q.P......H..........................RD......E..........................
SAMD9L_Caja  I...................S........Q........H.......K.....A............RD......E......R...................
SAMD9L_Mamu  .............................Q....................................RD......E..........................
SAMD9L_Loaf  IA............L.QI..........IQ.......S...........E..LF............KD..............R...........R.......
SAMD9L_Eqca  VA...A........L..I........P..E.......SSL........E..I.............RD......EF..Q.......KY.............
SAMD9L_Calu  IA............L..I........P..Q.L.....SH.F..I.....E..I.............KD..........Q....L.................
SAMD9L_Aime  AA............L..I........P..Q.......SH.FA.......E..T.............KD......E...Q...T..................
SAMD9L_Ereu  .S...........SL..L...........Q..K....S...........E..F.............GD......EC..E..R...................
SAMD9L_Orcu  VAH..S........L......................S...............T.............RD......E..........................
SAMD9L_Mumu  IK.D.AK............N........IQ.......SC.........MD..IMS...........KD......E.Q.......R........R.......
SAMD9L_Crgr  IKG..AE........Q...N........IQ.......SC.........IE..T.............RD.....NE.E..K...LR........RA......
SAMD9L_Rano  IK.D.AK.....................IQ.......SC.........MD..IMS...........RD......E.Q......LR........R.......
SAMD9L_Capo  IG...SK..V.......I.........L.Q...K...SY..........E..L....Q..S....K..............R..R................
SAMD9L_Soar  E.S..ED.T.....L.FL..............K....SK.....T....E..TF.T.........KD.....NA...Q..I..N..Q....S........

                     710       720       730       740       750       760       770       780       790       800
             ....|....|....|....|....|....|....|....|....|....|....|....|....|....|....|....|....|....|....|....|
SAMD9L_Hosa  FSSENYSSDFVKRDSYEKLKDLIHCWAESPKPIFAKIINLYHHPGCGGTTLAMHVLWDLKKNFRCAVLKNKTTDFAEIAEQVINLVTYRAKSHQDYIPVL
SAMD9L_Patr  ....................................................................................................
SAMD9L_Gogo  ......................V.............................................................................
SAMD9L_Poab  .......................R............................................................................
SAMD9L_Nole  ........................................................................................K...........
SAMD9L_Caja  ......P................Q.L..................................EK.............V..G...TS....K...........
SAMD9L_Mamu  .......................Q..................................................G....G.........K...........
SAMD9L_Loaf  ........A........H.....Q........V........................N.............A...T..GD..T..I..K.T...E.....
SAMD9L_Eqca  ........A..............L.R.Q....P....V...............N.................A......G...TS.I..K.T.........
SAMD9L_Calu  ........A...........V..K........E....................N......................E..V...TK.I..K.T..E..F...
SAMD9L_Aime  ........A..............Q...D....V....................N......S...............G..V...TK.I..K.T.PE..F...
SAMD9L_Ereu  ........-.I...R..E.RS..ED.......V....................N....FR.K.........A......G...T..IA.K.S.Y.......
SAMD9L_Orcu  ........A.......KE..N..Q......R.V.......................................A......G...T..I..K.TC........
SAMD9L_Mumu  ........A......F.E.TT..QQC.D....V.V.V.......................QK.........A...V..G...SK.MS.K.T..E.F....
SAMD9L_Crgr  ........A.....G..E.TT..QQC.D..E.V...V........................K.........A...L..G...SK.IS.K.T.........
SAMD9L_Rano  ........A.....NF.E.TT..QQC.D....V...V.......................QK.........A...I..G...SK.IS.K.S.........
SAMD9L_Capo  ........A.....M.KN.....ERC..C...E.....................I.....QKY........AI..EVG...I.H....K.T.........
SAMD9L_Soar  .....HT.A.....R..N..K..ED.......L....................N...E.R.K........I.A.....G...TK.I..K.S.........
```

```
                      810       820       830       840       850       860       870       880       890       900
             ....|....|....|....|....|....|....|....|....|....|....|....|....|....|....|....|....|....|....|....|
SAMD9L_Hosa  LLVDDFEEQENVYFLQNAIHSVLAEKDLRYEKTLVIILNCMRSRNPDESAKLADSIALNYQLSSKEQRAFGAKLKEIEKQHKNCENFYSFMIMKSNFDET
SAMD9L_Patr  ....................................................................................................
SAMD9L_Gogo  ....................................................................................................
SAMD9L_Poab  .....................L...............................................................K.IP...........
SAMD9L_Nole  ....................T...............................................................................
SAMD9L_Caja  .............I...V.............N...........Q.......S................................................
SAMD9L_Mamu  ....................................................................................................
SAMD9L_Loaf  ........L..ICI..D..N.S..K.G................Q...R...S...V..K....P......E...E.......................DM
SAMD9L_Eqca  ........P...CV.....QTIF....................Q.......S.Y....MHR..P.......D...........................I
SAMD9L_Calu  .......D....CV.....D.I.G..G................Q....T......V..T...........E...E......................NKM
SAMD9L_Aime  .......D.....V.....D.I....G................Q....T......V..T....P......E...E.................L....N.I
SAMD9L_Ereu  ............FV.......I.....................Q........D...V.K....P..K...ST...........F...............M
SAMD9L_Orcu  .............I.....N.I.....................Q........I.....K....P..........E.......D.........L.R.....
SAMD9L_Mumu  ...........A.I.....NAFI...G................Q.........N..S.K....P......E...Q....E............L.G...T.
SAMD9L_Crgr  ...........T.I...T.N.FI...G................Q...A........S.K....P......E...Q....E...........ML.GS.ET.
SAMD9L_Rano  .........Q.T.I.....N.FI...GV...............Q............S.K....P..K...E...Q....EY.D.........L.D...T.
SAMD9L_Capo  ...........I.I..........KR..Q......V.......Q...Q........S.K.A..A......E...E...................G....K
SAMD9L_Soar  ........L....N..D....S.....V...............Q..E...........TN...L..R............E...F........V.....D.

                      910       920       930       940       950       960       970       980       990      1000
             ....|....|....|....|....|....|....|....|....|....|....|....|....|....|....|....|....|....|....|....|
SAMD9L_Hosa  YIENVVRNILKGQDVDSKEAQLISFLALLSSYVTDSTISVSQCEIFLGIIYTSTPWEPESLEDKMGTYSTLLIKTEVAEYGRYTGVRIIHPLIALYCLKE
SAMD9L_Patr  ....................................................................................................
SAMD9L_Gogo  ....................................................................................................
SAMD9L_Poab  ................R............N................................................................V.....
SAMD9L_Nole  .............................N.............................N........................................
SAMD9L_Caja  ...................V.........N...............................................S................V.....
SAMD9L_Mamu  .............................N.........................................................V............
SAMD9L_Loaf  .............N...............N...IN.........F....TSAR......T..................................IA....
SAMD9L_Eqca  ....L.........A....T.........N...I...............MC..I..V.....N...A......N...T..R.............IS....
SAMD9L_Calu  ...K.........N.....G.........N....E..................M............A......N...T................IS....
SAMD9L_Aime  .............N.....G.........N...............................D....A......N.....F..............IF....
SAMD9L_Ereu  ......G.......G....T.........N..........P........T.IK...KQ...............NADI....-FP..........IF....
SAMD9L_Orcu  ..K............Y.............NT.........................K................N...S................IH....
SAMD9L_Mumu  ..K...K.T..DL.AK.RR.....Y....N...................T..KKYGK..TV.KN.........R...SD......I........TH....
SAMD9L_Crgr  ......K.T..DL.IH.RR.....Y....N.........L............NKHGK...V..N.......V.R...LD......I........IH....
SAMD9L_Rano  ..K...K.T..DL.AH.RK..F..Y....NT..I...............T..IKRGK..TV..N.........R...SD....A.I........IH....
SAMD9L_Capo  ...........E.NIN.............N.........L.............V..K......R....Y....S....................IH....
SAMD9L_Soar  .........F...NA....T....Y....N..........P........RH.PS..V...I..........I.....K...-.K......S....C....
```

```
                     1010      1020      1030      1040      1050      1060      1070      1080      1090      1100
             ....|....|....|....|....|....|....|....|....|....|....|....|....|....|....|....|....|....|....|....|
SAMD9L_Hosa  LERSYHLDKCQIALNILEENLFYDSGIGRDKFQHDVQTLLLTRQRKVYGDETDTLFSPLMEALQNKD-IEKVLSAGSRRFPQNAFICQALARHFYIKEKD
SAMD9L_Patr  ..............................................E....................-................................
SAMD9L_Gogo  ..............................................E....................-................................
SAMD9L_Poab  ..............................................E....................-................................
SAMD9L_Nole  ..............................................E....................-................................
SAMD9L_Caja  ..............K.........................V....RE....................-..M.............................
SAMD9L_Mamu  .................D............................E....................-................................
SAMD9L_Loaf  ..K......S.....L.R.K...V.....E..L............RE............I...P.SK-..N..T...IQ.............Y.......
SAMD9L_Eqca  ..K..D....K...K..K...............Q............EH...........I.....EE-.....VE..I......................K
SAMD9L_Calu  ..E..D.N......K..N.....V.....E...............RE.........A..I.....E.-.....I..AT..............Y......N
SAMD9L_Aime  ..N..N.N......K..K.D...V.....E................E.........A..I...E.EE-.....M..TL..............Y......N
SAMD9L_Ereu  .KK......SR...KL.S.....N....K.................G..G...-M....ID-..XXX-XXXXXXXX.N..............-L..H.RN
SAMD9L_Orcu  ..EKH..N.HH...KL.N......F..................R..E.............I.D....E-..E..V..CS......................N
SAMD9L_Mumu  ..M..RM........M....VL....L.....KY............EH.A.........I.E...EE-T....I...D.......................N
SAMD9L_Crgr  ..M..GMG....T......KV...........K.............EH.A....R....I.E.K.EE-T..I.TV..D.......................N
SAMD9L_Rano  ..M.HGM........M....I...........K.............EH.A.I......FI.E...EQ-T....T...D..................L...N
SAMD9L_Capo  ......M..........N.TV.........................E..A.........I.T.K.EE-VK...TR..S...E..................N
SAMD9L_Soar  ..K..Q..MSR....M.R.K........K..............H..EH...........I.D.K.E.IV.N..T...N...K...............K.N

                     1110      1120      1130      1140      1150      1160      1170      1180      1190      1200
             ....|....|....|....|....|....|....|....|....|....|....|....|....|....|....|....|....|....|....|....|
SAMD9L_Hosa  FNTALDWARQAKMKAPKNSYISDTLGQVYKSEIKWWLDGNKNCRSITVNDLTHLLEAAEKASRAFKESQRQTDSKNYET--ENWSPQKSQRRYDMYNTAC
SAMD9L_Patr  ...............................................................................--...................
SAMD9L_Gogo  ...............................................................................--...................
SAMD9L_Poab  ...............................................................................--.......Y...........
SAMD9L_Nole  ........H......................................................................--...................
SAMD9L_Caja  ........C.............................E.....N...SQ..........................C..--.T..........GT.....
SAMD9L_Mamu  ...............................................................................--.E.................
SAMD9L_Loaf  .....H..N...KI..........V......K......E..T.EV...K....F..T.....G........N...D..P--.V.PL..Y..K.......G
SAMD9L_Eqca  ..I..E..E..QKR..............H..K.....GE....KD........F............K..E...R.....--.A.L......K.V.....G
SAMD9L_Calu  .G...E..N...K..................Q......E...STD......IC......N..K...K..E..ER.G...WADT.AK.TL..K..T....G
SAMD9L_Aime  .S......NE..K..................R...R..EM..AKD......IC......N..K...K..E.AER.AC..--.P.G...L..K..T....G
SAMD9L_Ereu  ........Y...R.................N.V.....E.RS.KE..ID..M.C.D..........T..E...K.DS..--.A..T...LK..E.....G
SAMD9L_Orcu  .E...E..NL..TR.................K......E...SKV...K.............G......Q.........--.A..A........T....G
SAMD9L_Mumu  .S...V..NL..R...................L.Q..GK..T.GN.S.D..AYF..V.....K......N.S...D.G.--.A....N......TF...G
SAMD9L_Crgr  .S...V..NL..R.......................Q..GK.E...L.S.D......I....TK......E...N.D...--.A...R.......T....G
SAMD9L_Rano  .S...V..N...R......................RQ..------------------KV.....K......E.....D..I--.V...........T....G
SAMD9L_Capo  ..D..T..NL..ER......................C.-------------------.T.G..A...R...Q.....D...--.A.....P.....T....G
SAMD9L_Soar  .D...F..T...T...N....................EK....KI.SAS...YF........K...M..E...K.DC.R-GV.LQ.RFR.K.......G
```

```
                  1210      1220      1230      1240      1250      1260      1270      1280      1290      1300
            ....|....|....|....|....|....|....|....|....|....|....|....|....|....|....|....|....|....|....|....|
SAMD9L_Hosa FLGEIEVGLYTIQILQLTPFFHKENELSKKHMVQFLSGKWTIPPDPRNECYLALSKFTSHLKNLQSDLKRCFDFFIDYMVLLKMRYTQKEIAEIMLSKKV
SAMD9L_Patr ....................................................................................................
SAMD9L_Gogo ....................................................................................................
SAMD9L_Poab ..............................................................................................T.G...
SAMD9L_Nole ...........................................................................................T........
SAMD9L_Caja .....................Q......ENP...............K..Y............H.......................A....V.V......
SAMD9L_Mamu ............................I.T.................Y............T............................V..T.....
SAMD9L_Loaf ..........S.E......L.......AQ.PL......IGD.LA..E..YH.T.KN...Y.Q..RL...K.....V.......T.N.H...V..SI....
SAMD9L_Eqca ..........A........C..........S..E.....GN.LTNSKS.Y..V.R....Y.Q..........E..A......E.KN....T..LS.N..I
SAMD9L_Calu .F........A........C.L.Q......A..E.....GM..TN.KC.Y....N....Y.E.............D..L.....KN....TG..S....I
SAMD9L_Aime .V........A........C.L....F...A.AD.....GMT.TN.KC.Y.........Y.E.............N..I...Q..N....TV..S.N..I
SAMD9L_Ereu ..........A........C.....K..QR.T.E.....GN..EHSKGKY...I.......R.............A....F..T.N....T...I.....
SAMD9L_Orcu .F.....................D.VSFL.P........GD..F.A...Y.........L.Q....N..................N....M...L.....
SAMD9L_Mumu .F.......D...L.....L......I..ES.AE.....G..LS..KG.YCVV......L.Q..H...E...H..G...GF..P.N.P..LT.LL.....
SAMD9L_Crgr .F.......D.........P....S.IAIEA........G..S-..KD.YGAV....A...Q....E........L..IG...P.I.P..TT.LS.I...
SAMD9L_Rano .F.......D...L.....L......M..ES.A......G......KG.Y.VV.N..K.F.Q.............L...G...P.N.P..LT.LS.....
SAMD9L_Capo .......S.................D.S.LES........EN.S...K..Y........A..QS.....IK..H..S.......T.NF...MT.LK.....
SAMD9L_Soar .........FA......I.C.....D..E.R..K..L..VNV.T..K.DY....RQ...Y.Q...P.........A....F..T.TVH...T..S.R..I

                  1310      1320      1330      1340      1350      1360      1370      1380      1390      1400
            ....|....|....|....|....|....|....|....|....|....|....|....|....|....|....|....|....|....|....|....|
SAMD9L_Hosa SRCFRKYTELFCHLDPCLLQSKESQLLQEENCRKKLEALRADRFAGLLEYLNPNYKDA-TTMESIVNEYAFLLQQNSKKP----MTNEKQNSILANIILS
SAMD9L_Patr ........................H.................................-.....................----................
SAMD9L_Gogo ..........................................................-.....................----................
SAMD9L_Poab ..........................................................-A.........T.........H----................
SAMD9L_Nole ..............E...........................................-A...................R----................
SAMD9L_Caja ...............SDQ..N.G..............................K...SA.I............H.....C----................
SAMD9L_Mamu ...................R......F...........................F.N.A....I..D......K...N.R----...............N
SAMD9L_Loaf ..YY.....I....GLDP.....--......Y..S.........S.........H...AN.V.R...Q..L.F...LN.K----LIK....F........
SAMD9L_Eqca .......M.......LGV...R.....K......A.........S.......S.H.EVA....NV..K.T......PN.Q----L.R....F.......N
SAMD9L_Calu T...K..V.......SGP.HR-............A.........S.........HRE.A.N..N...K.N......P..Q----..K....F.......N
SAMD9L_Aime T......G....C..LG........FF...SF..G.........S.........HRE.A.N..N...K.S...R..PN.Q----L.K..L.F.......N
SAMD9L_Ereu ..Y..Q.I.....S.VGI..N.G.......YY..S...K.....S........SHQETGNI..N..E.........PN.R----.IK....F........
SAMD9L_Orcu ...YK..RD...I...SP.H.......L..........W.....S......SL.H.E.TS...N..D......K..PN.R----L.K....F........
SAMD9L_Mumu ....K..V.......TN.V.G..DL...K.....RIQ.W...T.S.........H.E.-NNI.N..GN.T....DILN.QLSKVL.KDI..F........
SAMD9L_Crgr A.Y.K..AG...RMNTN...G..NV.........RIV.W...T.S.........H.EV-DN.....KD.T....HSLS.RVTKGL.K.T..F........
SAMD9L_Rano ....K..AD...Q-E---..G..DL.........RIK.W...T.S.........H.E.-NN..N..EH.T....HTLN.QLSKALIKDT..F........
SAMD9L_Capo IF.YKT..K......STP..G.............R...W.....S.......S.H...A.I..N............PSIR----SIK....F........
SAMD9L_Soar GH..MS.ISI.N..ELGTT.AQ.NL.........R..KC.....SL............A..I....KD.T......LQN.----.RK....F........
```

```
                    1410      1420      1430      1440      1450      1460      1470      1480      1490      1500
             ....|....|....|....|....|....|....|....|....|....|....|....|....|....|....|....|....|....|....|....|
SAMD9L_Hosa  CLKPNSKLIQPLTTLKKQLREVLQFVGLSHQYPGPYFLACLLFWPENQELDQDSKLIEKYVSSLNRSFRGQYKRMCRSKQASTLFYLGKRKGLNSIVHKA
SAMD9L_Patr  ....................................................................................................
SAMD9L_Gogo  ....................................................................................................
SAMD9L_Poab  .......F...................P........................................................................
SAMD9L_Nole  ....K...................C...........................................................................
SAMD9L_Caja  .......S....N....................N.......................................H...........F..............
SAMD9L_Mamu  ....S.RF.....M...................D........................................................R.........
SAMD9L_Loaf  ....S..Y.R.FNI.........EL.EPT....D......................M...........GR..RH................S...H......
SAMD9L_Eqca  ....T..S....SK.....Q...HI..PR....D..........L.....E..Q.M...........KR..SN....R....V.....K...H.L....
SAMD9L_Calu  ....H..F....PI.........LSI.P.....N...........Q.....E...FM.........N.KR..RS...............Q...H.L.C..
SAMD9L_Aime  ....H..S....SI...L...A.LSI.P.Y...D.................E....M.........T.KR..RS...............K...H.L.C..
SAMD9L_Ereu  .......F....D.............L.EINR...D...........D..........M.......KK..NRH.RL..............N......L....
SAMD9L_Orcu  ....T..S....NM..........L...H...SD......................M.........T.....................F........F.GL.L..
SAMD9L_Mumu  ....S..Y.L.FS....K......I...T.S..D.............K...E..T...............R...H....R.P.......QK.....L....
SAMD9L_Crgr  ....S..Y.L.FN...TK.....EL...G.P..D.............K......T...............R...H.....KP.......QK...H.L....
SAMD9L_Rano  ....S..H.L.FS....K......I...T.S..D.............K......S...............R...H....R.P.......QK.....L....
SAMD9L_Capo  .V.....F....SI..Q.......L...NY...D..............K.......M....V......................F....TK.....L....
SAMD9L_Soar  ..N...VF.E..D...EL......LAK.D.S..D.F...........EV.....Q.L.......KK..NR..R.................NG...KGL...T

                    1510      1520      1530      1540      1550      1560      1570      1580      1590
             ....|....|....|....|....|....|....|....|....|....|....|....|....|....|....|....|....|....|...
SAMD9L_Hosa  KIEQYFDKAQNTNSLWHSGDVWKKNEVKDLLRRLTGQAEGKLISVEYGTEEKIKIPVISVYSGPLRSGRNIERVSFYLGFSIEGPLAYDIEVI
SAMD9L_Patr  E...........................................I................................................
SAMD9L_Gogo  ............................................I................................................
SAMD9L_Poab  ............................................I................................................
SAMD9L_Nole  E...........................................I............T................................K..
SAMD9L_Caja  E.....G.VH......Q.................I.........I....K.......................................Q..
SAMD9L_Mamu  E.......V...............H...H..C..I.........I................................................
SAMD9L_Loaf  E.....S.E.......Q.....................V.....I........R..............G.........................
SAMD9L_Eqca  ......G.V.......QN.....EK......C.........R..I....K...............Q...............M...........
SAMD9L_Calu  E.....N.....S..SQ.A..C..-K......C...........M.....K.V.....P.........G............M..LQ..E..I.
SAMD9L_Aime  E.....N....V...SP.......K......C............M.....K.......P.........G.........A......Q.....I.
SAMD9L_Ereu  E....IS.VK....F.Q..H....E...G.....N.R......LM..........................Q.........M...........
SAMD9L_Orcu  E.....S....I....Q.......K......H..V.........I............T..............K...................V
SAMD9L_Mumu  E..R..SEV.DS..F....V...ER.......L.D.........L.....A......T....A.........................G.K..
SAMD9L_Crgr  E..R.VSEV..S..F.Q..V...ER.......L.D...............A......T................V.............G.K..
SAMD9L_Rano  E..R..SEV.DS..F.Q..V...EG....I..L.D.........L.....A......T..............................G.K..
SAMD9L_Capo  EL.KHIS.TK....F.Q...I..T-...H..H............I...IN...T..ITPAFF.Q.....S..K.........G.......QIL
SAMD9L_Soar  E.....S.VP.SI...QN......K...S..C............IQ.......N...............H.QS........M.........L.
```
