# Supplementary material for: Evolution and divergence of the mammalian SAMD9/SAMD9L gene family
Source: BMC Evol Biol. 2013 Jun 12;13:121. doi: 10.1186/1471-2148-13-121 (PMC3685527; doi:10.1186/1471-2148-13-121)
Supplement: Additional file 9: Table S2 — SAMD9 and SAMD9L likelihood ratio test (LRT) for PARRIS analysis from HyPhy software. Only SAMD9L was found to be under selection when using this specific method. [file 1471-2148-13-121-S9.pdf]

Table S2: *SAMD9* and *SAMD9L* likelihood ratio test (LRT) for PARRIS analysis from HyPhy software

Hypothesis		LRT		
Null Hypothesis	Alternative Hypothesis	-2ΔlnL	df	p Value
PARRIS				
SAMD9				
M1: no selection	M2: selection	0.2	2	n.s.
SAMD9L				
M1: no selection	M2: selection	8.4	2	**< 0.05***

*, significant; n.s., not significant
