# Supplementary material for: Evolution and divergence of the mammalian SAMD9/SAMD9L gene family
Source: BMC Evol Biol. 2013 Jun 12;13:121. doi: 10.1186/1471-2148-13-121 (PMC3685527; doi:10.1186/1471-2148-13-121)
Supplement: Additional file 10: Table S3 — Positively-selected codon positions in SAMD9 and SAMD9L determined by six different Maximum Likelihood methods. The six methods correspond to PAML M8, SLAC, FEL, REL, MEME and FUBAR. Codons positions are numbered according to human SAMD9 and SAMD9L proteins (Additional file 5 Figure S4 and Additional file 6 Figure S5). [file 1471-2148-13-121-S10.pdf]

Table S3: Positively-selected codon positions in *SAMD9* and *SAMD9L* determined by six different Maximum Likelihood methods

	SAMD9	***SAMD9L***
PAML M8[a]	21*, **48***, 52, **88**, **491**, 736, **1006***, 1085**, 1088, **1116***, 1120*, **1258***, 1348, 1380, **1398***, 1440	71**, 126**, 146*, **156**, **260**, **267***, 329*, **340****, **357***, 368*, **452****, **586***, 639, **653***, 1145*, 1183, **1276***, 1295**, 1318*, 1339, 1380*, 1408*
SLAC[b]	**352**, **383**, **491***, **513**, **731**, **1006***, **1116**, **1320***, **1329**, **1398***	**156**, **267***, **340****, **362***, **586***, **606**, **776***, **978***, 1018, **1186**, **1229**, **1429**, **1474**
FEL[b]	38, 45*, 75*, **88***, 151, 170*, 176, **279***, **331**, **352***, **383***, 395*, **491***, **513***, 596*, 610, 618*, **731**, 735, 783*, **872**, 875*, **993***, **1006****, 1095, **1116****, 1127, **1258***, **1320***, **1329***, 1333, 1351, 1376, 1393, **1398****, 1424, 1503, 1528	5, **39***, 56, 76, 83, 88, 92, **156***, **260**, **267****, 282, **340****, **357***, **362****, **452****, 519, 540, 585, **586***, 602, **606***, **653***, **776***, 783, **978***, 1006, 1154*, **1186**, 1189*, **1229***, 1255, **1276**, **1308**, 1360*, 1364, 1374, 1413, **1429***, **1474***
REL[c]	**48**, 52, 69, 106, 116, 193, **279**, 312, **331**, 372, 376, 397, **491**, 594, 611, **731**, 736, 827, **872**, 956, **993**, **1006**, 1017, 1085, 1088, 1092, **1116**, 1120, 1167, 1177, **1258**, 1314, **1320**, 1327, **1329**, 1348, 1380, **1398**, 1519, 1576	**39**, 126, **156**, **260**, **267**, 279, 325, 328, **340**, **357**, **362**, **452**, **586**, 601, 639, **653**, 721, **776**, 822, 953, **978**, 996, 1018, 1078, 1106, 1109, 1183, **1229**, 1231, **1276**, 1295, **1308**, 1316, 1335, 1367, 1380, 1413, **1429**, 1535
MEME[b]	21, 38**, 45*, **48**, 61*, 75*, 77*, 79**, 85**, **88***, 96, 109, 121, 143, 145, 170, 173, 176, 187, 191, 193, 201**, 218*, **279**, **331***, **352****, 353, 365, 369, **383**, 387, 395, **491***, **513***, 596, 610, 618, 623*, 642*, **731**, 735**, 744, 783, 859, **872**, 875*, 969**, **993***, 1003*, **1006****, 1007, 1041, 1063, **1116***, 1174*, 1199, 1252, **1258***, **1320***, **1329***, 1341*, 1347*, 1351*, 1376*, 1393, **1398****, 1415, 1422, 1444**, 1467*, 1528, 1556**, 1566*	5, 24*, **39***, 76*, 92, 142*, 149*, **156***, 170, 181*, 212*, 213*, **267****, 282, 299, 318, 323*, **340****, 341**, 350, 351, **357***, 359, **362***, 366, 373*, 377*, 426*, **452****, 468*, 509, 519*, 540, 555*, 585, **586***, 602, **606**, 632**, **653**, 723, 725*, 754, **776***, 789*, 791*, 848, 856, 859, 861*, 888**, 889*, 945*, 963, **978***, 1004*, 1006*, 1109*, 1150*, 1154, 1170, **1186***, 1189, **1229***, 1250, 1252*, **1276***, 1284, 1287, **1308**, 1335**, 1339*, 1343, 1346, 1360*, 1374*, 1385, 1390, **1429***, **1474***, 1535
FUBAR[d]	**1006**, **1398**	**156**, **267***, **340****, **357**, **452**

[a]Codons with posterior probabilities >90% in the BEB analyses (*: P>95%; **: P>99%)

[b]Codons with significance level <0.1 (*: p<0.05; **: p<0.01)

[c]Codons with Bayes Factor >50

[d]Codons with posterior probabilities >0.90 (*: P>0.95; **: P>0.99)

Codons identified by three or more than three methods are underlined and in bold.
