# Supplementary material for: Evolution and divergence of the mammalian SAMD9/SAMD9L gene family
Source: BMC Evol Biol. 2013 Jun 12;13:121. doi: 10.1186/1471-2148-13-121 (PMC3685527; doi:10.1186/1471-2148-13-121)
Supplement: Additional file 11: Table S4 — SAMD9 and SAMD9L amino acid properties under positive selection determined in TreeSAAP. SAMD9 exhibited three and SAMD9L evidenced seven amino acid properties under positive selection. [file 1471-2148-13-121-S11.pdf]

Table S4: SAMD9 and SAMD9L amino acid properties under positive selection determined in TreeSAAP

Protein	Property	Category[a]	z-Score[b]
**SAMD9**	Isoelectric point	8	8.218***
	Equilibrium constant (ionization of COOH)	8	5.001***
	Power to be at the C-terminal	7	4.032***
	Alpha-helical tendencies	6	5.581***
**SAMD9L**	Isoelectric point	8	6.922***
	Equilibrium constant (ionization of COOH)	8	4.797***
	Solvent accessible reduction ratio	7	4.366***
	Power to be at the middle of alpha-helix	7	3.554***
	Power to be at the C-terminal	6	10.442***
	Alpha-helical tendencies	6	5.228***
	Surrounding hydrophobicity	6	3.921***

[a]Between 6 and 8 (the most radical values denoting positive destabilizing selection).
[b]z-Score > 3.09, level of significance p<0.001 (***).
